# Stereodivergent Chirality Transfer by Noncovalent Control of Disulfide Bonds

**DOI:** 10.1021/jacs.1c10000

**Published:** 2022-02-04

**Authors:** Qi Zhang, Stefano Crespi, Ryojun Toyoda, Romain Costil, Wesley R. Browne, Da-Hui Qu, He Tian, Ben L. Feringa

**Affiliations:** † Key Laboratory for Advanced Materials and Joint International Research Laboratory of Precision Chemistry and Molecular Engineering, Feringa Nobel Prize Scientist Joint Research Center, Frontiers Science Center for Materiobiology and Dynamic Chemistry, Institute of Fine Chemicals, School of Chemistry and Molecular Engineering, East China University of Science and Technology, Shanghai 200237, China; ‡ Stratingh Institute for Chemistry and Zernike Institute for Advanced Materials, University of Groningen, Nijenborgh 4, 9747 AG Groningen, The Netherlands

## Abstract

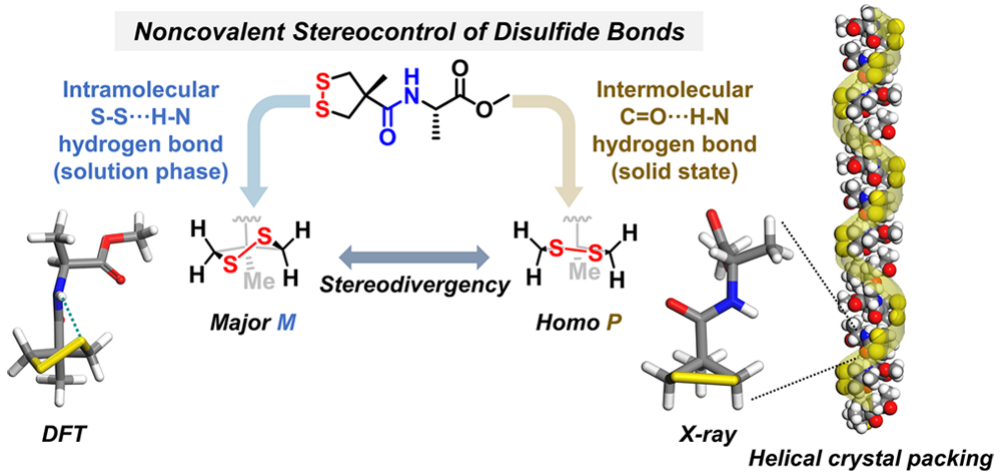

Controlling dynamic stereochemistry is an important challenge, as it is not only inherent to protein structure and function but often governs supramolecular systems and self-assembly. Typically, disulfide bonds exhibit stereodivergent behavior in proteins; however, how chiral information is transmitted to disulfide bonds remains unclear. Here, we report that hydrogen bonds are essential in the control of disulfide chirality and enable stereodivergent chirality transfer. The formation of S–S···H–N hydrogen bonds in solution can drive conformational adaption to allow intramolecular chirality transfer, while the formation of C=O···H–N hydrogen bonds results in supramolecular chirality transfer to form antiparallel helically self-assembled solid-state architectures. The dependence on the structural information encoded in the homochiral amino acid building blocks reveals the remarkable dynamic stereochemical space accessible through noncovalent chirality transmission.

## Introduction

**Figure 1:**
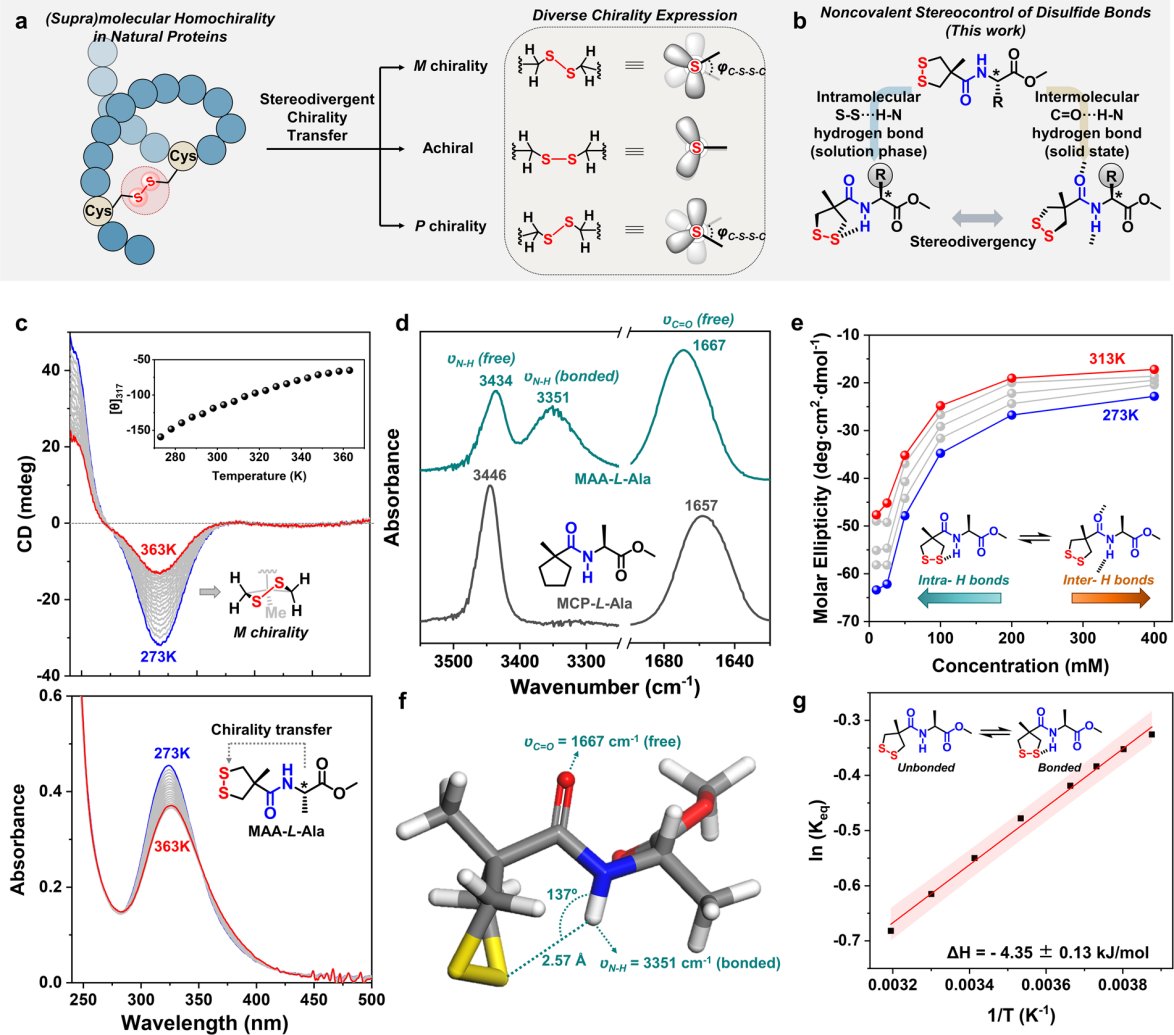
Conceptual illustration of and experimental data for intramolecular chirality transfer mediated by S–S···H–N hydrogen bonds. (a) Stereodivergent chirality transfer of disulfide bonds in natural proteins. (b) Hydrogen-bond-controlled chirality transfer pathway enables the stereodivergency of disulfide bonds in this study. (c) Temperature-dependent CD and UV–vis absorption spectra of MAA–l-Ala in MCH (2 mM). Inset curve shows the molar ellipticity ([θ], unit: deg·cm^2^·dmol^–1^) at 317 nm as a quasilinear function of temperature. (d) Partial FTIR spectra of MAA–l-Ala and MCP–l-Ala in CDCl_3_ solution (10 mM). (e) Concentration-dependent molar ellipticity of MAA–l-Ala at the temperature region from 273 to 313 K in CHCl_3_. (f) Energy-minimized molecular conformation of MAA–l-Ala simulated by DFT (ωB97X-D/def2-TZVP). (g) Van’t Hoff fitting plot of MAA–l-Ala in CDCl_3_ according to the combined spectroscopic information on temperature-varied ^1^H NMR spectra and solution-phase FTIR spectra. The entropy change (Δ*S*) was estimated to be around 20 J·mol^–1^·K^–1^. The error analysis was obtained by the linear fitting of eight data points. The red band indicates a 95% confidence interval.

Homochirality, being a “signature of life”, is a unique feature enabling nature to transfer molecularly encoded information and control geometry and structure along length scales from the molecular and supramolecular level all the way up to macroscopic scales.^1^ Beyond the intrinsic (static) chirality in homochiral building blocks such as amino acids, the control of dynamic or adaptive chiral structures, e.g., conformational, supramolecular, or macromolecular chirality, plays an essential role to sustain key functions of life.^2−4^ The underlying mechanisms for the transmission of chirality and the multifaceted pathways to chirality transfer are clearly of fundamental importance. Typically, disulfide bonds, which commonly bridge peptide chains,^5^ show inherent dynamic stereoisomerism (Figure 1a) and are key in determining the structures and functions of numerous disulfide-containing proteins in nature^6−9^ and synthetic materials.^10^ The stereochemistry of disulfide bonds, including dihedral angles and inherent chirality,^11,12^ is a distinctive feature defining the optical, chemical, and biochemical properties, especially for the cyclic disulfides found in many biological small molecules and enzymes.^13−18^ The dihedral angle of disulfide bonds can be controlled by modulating the ring strain of cyclic disulfides.^11,14,15^ However, how chiral information is transmitted from amino acid units to disulfide bonds and how a diversity of chirality is expressed at the molecular and supramolecular level based on similar homochiral constituents remains unclear. Here, we report the discovery that disulfide bonds can receive chiral information from amino acids via two distinct noncovalent pathways, that is, (i) intramolecular chirality transfer by forming S–S···H–N hydrogen bonds or (ii) supramolecular chirality transfer in helical assemblies (Figure 1b). Furthermore, we observed S–S···H–N hydrogen-bond-controlled stereodivergent central-to-axial chirality transfer from amino acid to disulfide units.

## Results and Discussion

While exploring 1,2-dithiolanes,^19^ we envisioned that this simple structural unit could serve as an ideal model for investigating disulfide stereochemistry, because the cyclic, yet conformationally flexible and stereodynamic, 1,2-dithiolanes exhibit red-shifted electronic absorption spectra compared to linear disulfides,^20^ enabling the unambiguous spectroscopic characterization of induced chirality by circular dichroism (CD).^21^ Cyclic disulfides are also widely present in natural proteins and small molecules.^14,15,17^ Coupling a symmetrical 1,2-dithiolane, methyl asparagusic acid (MAA), with enantiopure l-alanine methyl ester, provided MAA–l-Ala, which surprisingly exhibited a strong negative CD band at 317 nm in apolar solvents, such as methyl cyclohexane (MCH), suggesting the predominant *M*-helicity of the disulfide bonds (Figure 1c).^15^ Inverting the chirality of the amino acid led to typical mirror-symmetric CD spectra of MAA–d-Ala (Supplementary Figure S1), indicating the central-to-axial chirality transfer from the amino acid to the disulfide bond. The molar ellipticities of MAA–l-Ala decreased with an increase in temperature between 273 to 363 K (Figure 1c, inset) and an increase in solvent polarity (Supplementary Figure S2), indicating a relation between chirality transfer and hydrogen bond formation.

Considering the fact that in some X-ray crystal structures of natural proteins^22^ sulfur atoms have been shown to participate in the formation of hydrogen bonds (Supplementary Figure S3), we propose that the sulfur atoms in MAA–l-Ala act as hydrogen-bonding acceptors for the amide protons, and the resulting intramolecular S–S···H–N hydrogen bonds enable an effective “long-range” chirality transfer across four atoms in the molecular skeleton. To verify this, various spectroscopic measurements were used to probe the amide bonds in dilute solutions of MAA–l-Ala (Supplementary Figures S4–S12). Fourier transform infrared (FTIR) spectroscopy in solution showed the coexistence of bonded and free, i.e. solvated, amide bonds (ν_N–H_ = 3351 and 3434 cm^–1^), and the free carbonyl group (ν_C=O_ = 1667 cm^–1^) of the amide (Figure 1d) indicating the formation of intramolecular S–S···H–N hydrogen bonds instead of intermolecular C=O···H–N hydrogen bonds. Variable-temperature nuclear magnetic resonance (VT-NMR) spectroscopy revealed a concentration-independent shift in the resonance of the amide proton (Δδ_N–H_/Δ*T* = −1.6 ppb/K) with temperature at low concentrations (2–20 mM CDCl_3_; Supplementary Figures S5–S11), indicating it is present in a molecularly dissolved (nonaggregated) state. Moreover, the observation of dilution-enhanced molar ellipticity (Figure 1e and Supplementary Figure S4) further confirmed the intramolecular chirality transfer mechanism.

Density functional theory (DFT) was used to search the globally energy-minimized geometry to understand the S–S···H–N hydrogen bonds (Supplementary Figure S13). A thermodynamically stable conformation (46% of the population based on calculation in vacuo) was obtained that is highly consistent with our experimental observations (Figure 1f): (i) The amide proton points to one of the two sulfur atoms with a distance of 2.57 Å and favorable bond angle (φ_S···H–N_ = 137°); (ii) the disulfide bond exhibits a predominant conformation with *M*-helicity with a dihedral angle of 42°. Furthermore, the simulated CD spectrum shows excellent correspondence with the experimental spectrum (Supplementary Figure S14). Analysis based on two-dimensional nuclear Overhauser effect NMR spectroscopy (2D NOESY) also supported the preference for the simulated conformation (Supplementary Figure S15). Based on the cumulative data, it is clear that the formation of intramolecular S–S···H–N hydrogen bonds is responsible for the observed chirality transfer to the cyclic disulfide unit.

The reference molecule, MCP–l-Ala (Figure 1d), was prepared to gain quantitative insight into this unique S–S···H–N hydrogen bond. Its cyclopentane ring enables a close approximation of the spectroscopic information (FTIR and VT-NMR in diluted solutions, Figure 1d and Supplementary Figures S16–S20);^23^ the van’t Hoff plot indicates that the intramolecular hydrogen bonded state of MAA–l-Ala was 4.35 kJ·mol^–1^ enthalpically more favorable and around 20 J·mol^–1^ K^–1^ entropically less favorable than the unbonded state (Figure 1g and Supplementary Figure S21). This is the first time, to the best of our knowledge, to thermodynamically characterize S–S···H–N hydrogen bonds, indicating a moderate strength compared with common C=O···H–N hydrogen bonds (5–7 kJ·mol^–1^).^23^

A series of analogues of MAA–l-Ala was prepared (Figure 2a) and characterized by CD (Supplementary Figures S22–S34), FTIR (Supplementary Figure S35), VT-NMR (Supplementary Figures S36–S47), and 2D NOESY spectroscopy (Supplementary Figures S48–S56) to explore the general nature and (stereo-) chemical space. These combined data revealed the general nature of the intramolecular chirality transfer mediated by S–S···H–N hydrogen bonds as present in all MAA–amide analogues. The values of the dissymmetry *g*-factor were used to quantitatively compare the chirality transfer efficiency of these analogues (Figure 2a).^24^ Notably, the presence of a carboxylic methyl ester at the stereocenter can enhance the *g*-factor values 2- to 3-fold compared to substitution with phenyl and cyclohexane groups, which may be attributed to carbonyl–carbonyl interactions^25^ favoring energy-minimized rotamers.

**Figure 2:**
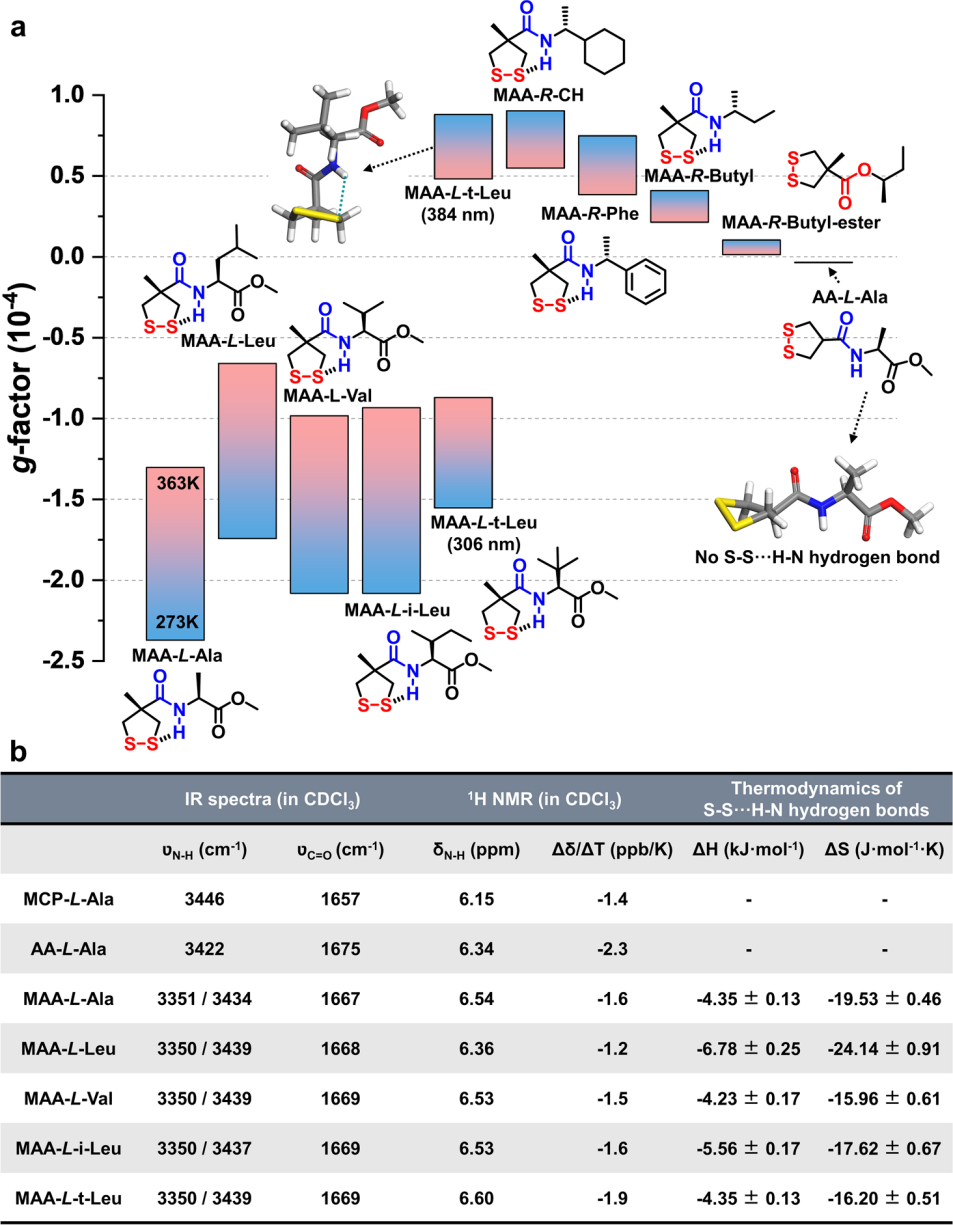
Structural factors that determine the pathway of intramolecular chirality transfer. (a) Temperature-dependent dissymmetry *g*-factors of a series of chiral amide analogues. Except AA-l-Ala using CHCl_3_ as the solvent due to the solubility, all other compounds were measured in molecularly dissolved MCH solutions at varying temperatures from 273 to 363 K. The insert molecular geometry shows the energy-minimized conformers of MAA–l-t-Leu and AA-l-Ala by DFT (ωB97X-D/def2-TZVP). (b) Spectroscopic and thermodynamic data of the 1,2-dithiolane compounds with different substituents (see experimental details in the Supporting Information).

One of the signature features of amino acids comes from the diversity of the substituent at the stereogenic center, which contributes to the complexity of natural protein architectures. Exploring the effect of the substituent (Figure 2 and Supplementary Figures S57–S60), it was observed that introducing a substituent with increased steric hindrance, i.e., MAA–l-t-Leu, shifts the CD band toward 370 nm with a positive Cotton effect (*P*-helicity) (Supplementary Figure S34). Going from MAA–l-Ala to MAA–l-t-Leu, the predominant disulfide helicity changes from *M* to *P* (Supplementary Figures S61 and S62). DFT simulation showed the existence of an energy-minimized conformer with a twisted S–S···H–N hydrogen bond (Supplementary Figure S61) as well as chirality inversion of the disulfide bond (i.e., *P*-helicity with a small dihedral angle of 13°), which is responsible for the positive CD band observed at 370 nm (Supplementary Figure S62). Considering the spectroscopic and thermodynamic data (Figure 2b), it can be inferred that, in this system, the substituent groups affect the stereoisomerism of disulfide bonds by steric hindrance, leading to preferred rotamers, and, as a consequence, by geometry change of the S–S···H–N hydrogen bonds lead to the reversal of helical S–S chirality.

The methyl substitution at the 1,2-dithiolane ring also acts as a crucial structural factor. In the absence of a methyl substituent, i.e., AA-l-Ala, neither S–S···H–N hydrogen bond nor efficient intramolecular chirality transfer was observed: (i) The ν_N–H_ band in IR spectra (ν_N–H_ = 3422 cm^–1^) showed a nearly completely free state (Supplementary Figure S35); (ii) the chemical shifts of the four methylene protons overlap instead of showing clear coupling patterns in their ^1^H NMR spectra (Supplementary Figures S44–S46); (iii) the *g*-factor of AA-l-Ala was only 1.7% of that of MAA–l-Ala (Figure 2a), meaning inefficient chirality transfer. DFT simulation of AA-l-Ala (and comparison with MAA–l-Ala) revealed the inner mechanism of the angle compression by introducing a methyl substituent, i.e., the so-called Thorpe-Ingold effect,^26^ facilitating the intramolecular cyclization by forming S–S···H–N hydrogen bonds (Supplementary Figures S63 and S64).

**Figure 3:**
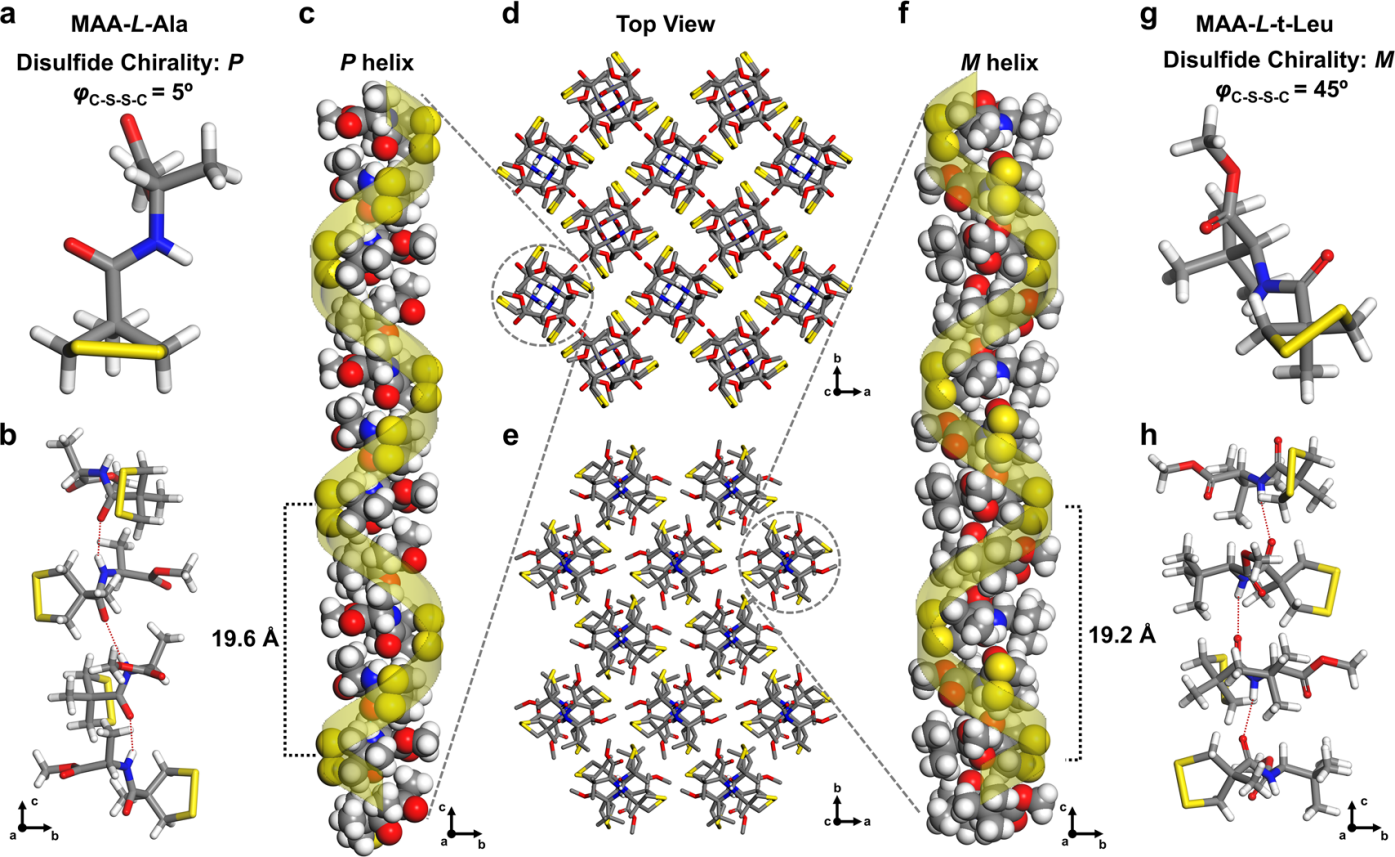
X-ray single-crystal structures of MAA–l-Ala and MAA–l-t-Leu. (a) Single-molecular structure of MAA–l-Ala in the asymmetric unit. (b) A tetramer unit of MAA–l-Ala formed by intermolecular hydrogen bonds (indicated as red dot lines) along the *c*-axis, resulting in one pitch of the helical strand. (c) A representative right-handed helical strand formed by the self-assembly of the 12-mer MAA–l-Ala. (d) View along the *c*-axis showing the self-organized pattern consisting of 12 homochiral helical strands, in which every two neighboring strands are side-by-side stacked in an antiparalleled manner by van der Waals interactions. (e–h) Corresponding supramolecular packing architectures (e), helical self-assembly (f,h), and molecular unit structure (g) of MAA–l-t-Leu in the solid state. For clarity, the helical strand (in yellow) is superimposed over an ideal helical model. Carbon, gray; hydrogen, light gray; nitrogen, blue; oxygen, red; sulfur, yellow.

The subtle interplay of hydrogen bonding, conformational effects, and chirality transfer prompts the question: How will this translate to self-assembled architectures in the solid state? Upon crystallization by slow evaporating of mixtures of diethyl ether and heptane, X-ray single-crystal structural analysis (Supplementary Figures S65–S69, Table S1–S4) revealed much to our surprise that the structurally simple building block of MAA–l-Ala self-assembled into a supramolecular architecture with high complexity in the solid state (Figure 3a–d), without inclusion of solvent molecules. Notably, the disulfide stereoisomerism of MAA–l-Ala in the crystal structure was remarkably different from that in solution (Figure 3d); the disulfide bonds exhibited a nearly planar conformation with a dihedral angle of only 5°, which is unusual because of the high energy usually associated with planar disulfide bonds (Supplementary Figure S3b). Such nearly planar disulfides were only observed so far in a few cases of highly strained cyclic disulfides.^15,27^ The disulfide bonds exhibited homochirality (*P*) in the solid state, further confirming that the *M*-preferred disulfide bonds in diluted solution were due to intramolecular, instead of intermolecular, hydrogen bonds.

In the crystal structure, the amide protons, instead of binding to the sulfur atoms, formed intermolecular hydrogen bonds with carbonyl groups (Figure 3b), connecting the building blocks of MAA–l-Ala into one-dimensional assemblies along the *c* axle. An intriguing feature is that the orientation of the molecules shows a twisted arrangement around the *c* axle by 90° per two molecules, thus forming helical strands with every four units as one repeating sequence (Figure 3c). All the helical strands bear *P*-type supramolecular helicity and furthermore assemble into three-dimensional architectures in an antiparallel packing (Figure 3d). The unique features of helical chirality and antiparallel self-assembly are reminiscent of biological architectures like the DNA double helix, and the antiparallel *β-*sheet in peptides.

The solid-state structures of analogues were examined to explore the structure-assembly relations, and not unexpectedly, the crystal structure of MAA–d-Ala showed a mirror-image solid state architecture compared to MAA–l-Ala (Supplementary Figures S66 and S67). Interestingly, the absence of a methyl substitution at the 1,2-dithiolane ring, i.e., AA-l-Ala, inhibited the supramolecular helical assembly and preferred chirality transfer in the crystal structure of AA-l-Ala (Supplementary Figure S68), with a β-sheet-like packing instead devoid of helical supramolecular organization. The disulfide bonds are present with a 1:1 ratio of *P*- and *M*-chirality. The absence of the ring-methyl substituent resulted in a nonhelical arrangement, and the distinct self-assembly can be attributed to the diminished steric hindrance and the more planar molecular conformation favoring the β-sheet like packing. The simultaneous disappearance of supramolecular helicity and disulfide homochirality suggests the mutual dependence of molecular and supramolecular chirality transfer.

In contrast, the crystal structure of MAA–l-t-Leu (Figure 3e–h) exhibited a similar architecture as MAA–l-Ala featuring helical geometry and antiparallel helical strand orientation (Figure 3e,f). Remarkably, the disulfide chirality and supramolecular helicity were synchronously reversed into *M*-type (Figure 3f–h), instead of the *P*-type shown in MAA–l-Ala. Transmission from central chirality, with identical handedness, to helical chirality with the opposite configuration in MAA–l-t-Leu and MAA–l-Ala, is observed, which is surprising, as the origin of chirality is both from left-handed amino acids. The key factor that leads to the difference of molecular self-assembly may stem from the bond compression effect, i.e., the Thorpe-Ingold effect, of the bulky t-Leu residue group, which decreases the angle of N–C_6_–C_7_ from 112.3 to 107.6° (Figure S70), thus being subtly amplified by the H-bonding self-assembly in crystal architecture. This inversion and reversed transmission of chirality both at the molecular and supramolecular level brought by the change of residue groups are very unusual when realizing the fact that all the α-helixes in natural proteins are homochiral due to the homochirality of natural amino acids.

**Figure 4:**
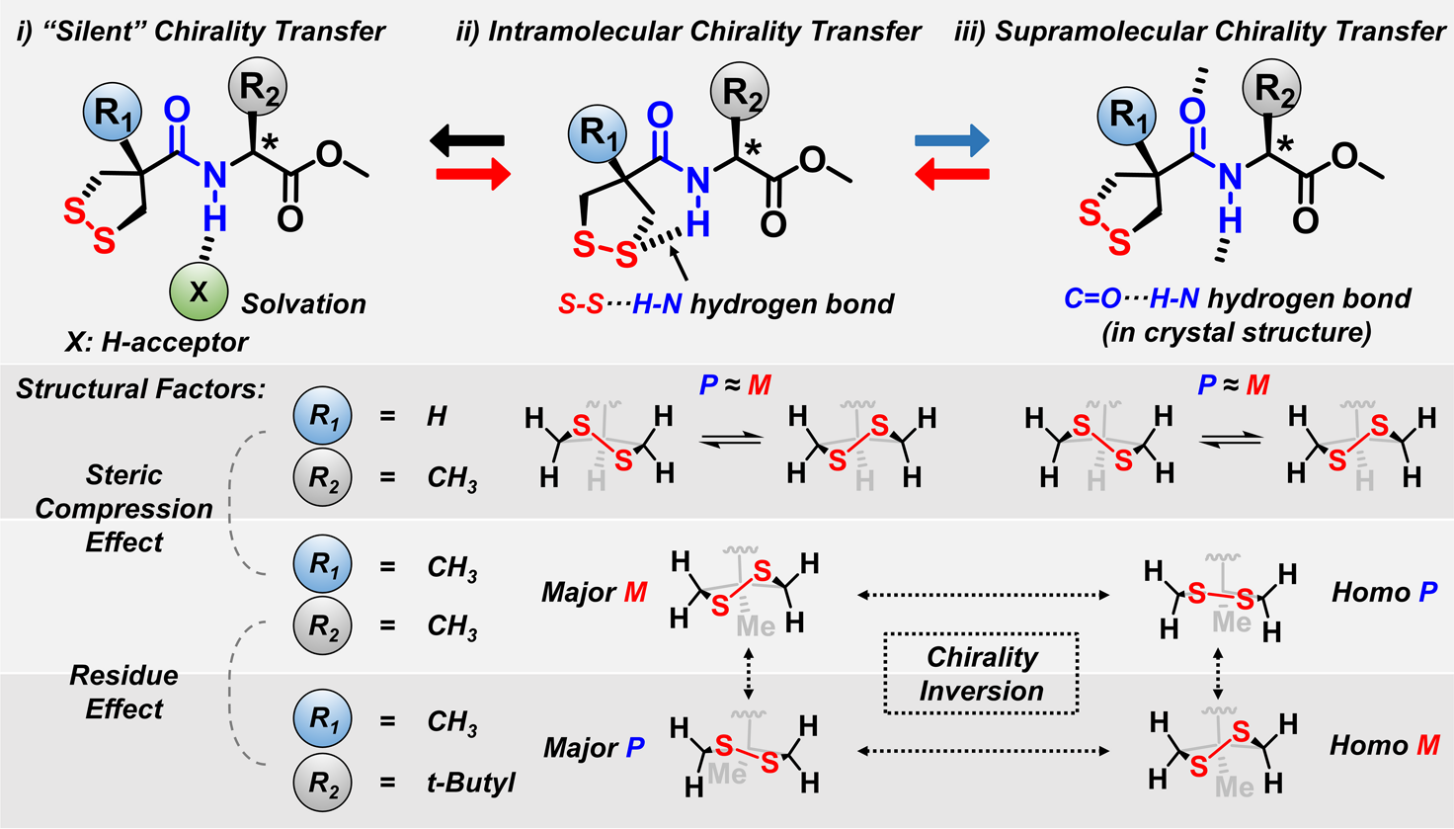
Stereodivergent chirality transfer enabled by noncovalent control of disulfide bonds. In the model system, the central-to-axial chirality transfer process can be diversified by subtly controlling the hydrogen bonds of the amide group. The solvation of amide protons inhibits efficient chirality transfer. The amide protons can form S–S···H–N hydrogen bonds with sulfur atoms in diluted solutions to enable intramolecular chirality transfer, while the chiral information is delivered at the supramolecular scale in the solid state supported by intermolecular C=O···H–N hydrogen bonds. The disulfide stereochemistry of the same molecules in solution or solid states is opposite, indicating the stereodivergency. Multifaceted chirality transfer can be encoded by molecular information, i.e., R_1_ and R_2_ groups, to show the stereodivergency based on similar homochiral units.

The multifaceted chirality transfer in the noncovalent stereocontrol of disulfide bonds that we observed is summarized in Figure 4. In the amino-acid-functionalized cyclic disulfides, the central-to-axial chirality transfer can be controlled by the subtle changes in hydrogen bonding and steric (substituent) parameters. Both in solution and in the solid state, stereodivergent chirality depends on the delicate interplay of at least three elements, that is, (i) the nature of the hydrogen bonding including solvation, intramolecular S–S···H–N and intermolecular C=O···H–N hydrogen bonds; (ii) the Thorpe-Ingold effect of ring R_1_-substituent and; (iii) the amino acid substituent R_2_ at the stereogenic center. Notably using the same natural homochirality, it is evident that subtle structural factors and the change in nature of the hydrogen bonding allow remarkable stereodiversity in chirality transfer and the formation of both *P*- or *M*-helicity at the molecular as well as the supramolecular level. Minor changes in the chiral molecular structures and distinct hydrogen bond geometries have major effects on the transmission of chiral information and can lead to either elimination of chirality in other dynamic chiral elements or result in the complete reversal of helicity. These fundamental insights will guide the exploration of chirality-encoded molecular information and in particular dynamic stereochemistry at S–S bonds highly relevant for disulfide-containing supramolecular architectures.

## Supporting Information Available

Details on the synthesis and structural characterizations of compounds, NMR spectra, CD spectra, FTIR spectra, DFT simulations, thermodynamic characterizations, crystallographic data, including Figures S1–S109 and Tables S1–S4 (PDF)
Conformer files of the simulated molecular structures (ZIP)

## Accession Codes

CCDC 2099415–2099418 contain the supplementary crystallographic data for this paper. These data can be obtained free of charge via www.ccdc.cam.ac.uk/data_request/cif, or by emailing data_request@ccdc.cam.ac.uk, or by contacting The Cambridge Crystallographic Data Centre, 12 Union Road, Cambridge CB2 1EZ, UK; fax: +44 1223 336033.
