# Supplementary material for: Stereodivergent Chirality Transfer by Noncovalent Control of Disulfide Bonds
Source: J Am Chem Soc. 2022 Feb 4;144(10):4376–82. doi: 10.1021/jacs.1c10000 (PMC8931715; doi:10.1021/jacs.1c10000)
Supplement: Supplementary file 1 — ja1c10000_si_001.pdf [file ja1c10000_si_001.pdf]

Supplementary Information for

Stereodivergent Chirality Transfer by Noncovalent Control of Disulfide Bonds

Qi Zhang,[1, 2] Stefano Crespi,[2] Ryojun Toyoda,[2] Romain Costil,[2] Wesley R. Browne,[2] Da-Hui Qu[1]*, He Tian,[1] Ben L. Feringa[1, 2]*

[1] *Key Laboratory for Advanced Materials and Joint International Research Laboratory of Precision Chemistry and Molecular Engineering, Feringa Nobel Prize Scientist Joint Research Center, Frontiers Science Center for Materiobiology and Dynamic Chemistry, School of Chemistry and Molecular Engineering, East China University of Science and Technology; 130 Meilong Road, Shanghai 200237, China.*

[2] *Stratingh Institute for Chemistry and Zernike Institute for Advanced Materials, Faculty of Science and Engineering, University of Groningen; Nijenborgh 4, 9747 AG Groningen, The Netherlands.*

*Corresponding author. Email: dahui_qu@ecust.edu.cn; b.l.feringa@rug.nl

Table of Contents:

Materials and Methods

Figs. S1 to S109

Tables S1 to S4

References (*S1*-*S5*)

Additional Materials

Data S1. Conformer files of the simulated molecular structures.

Materials and Methods

Materials

All the reagents were obtained from commercial sources (Sigma-Aldrich and TCI) and used as received without further purification. Solvents used for reactions were HPLC grade. Solvents used for spectroscopic experiments were spectroscopic grade (TCI) or NMR grade (Sigma-Aldrich). Solvents for NMR spectroscopy were used as received from Sigma-Aldrich. TLC plates were used to trace the reactions and visualized by iodine silica bath. Column chromatography was performed using Silica 90 Å as the stationary phase.

Instruments and Methods

VT-CD/UV-Vis spectra

All the CD/UV-Vis spectra were collected on a JASCO-810 CD spectrometer. The temperature was controlled by a temperature-controlling accessory. Quartz cuvettes were used to support the sample solutions. If no external indication, the optical path of the quartz cuvettes was 10 mm. An automatic optical shutter was used to inhibit the UV-light-triggered disulfide scission before the start of the measurement. Some very weak signals at around 500 nm were attributed to the absorption of the minimal sulfur radicals, which didn't affect the main experimental results. The temperature-varied rate was set as 5 ^oC/min. Measurement was collected after the real-time temperature was fixed around the target temperature (± 0.1^{o}C) for 30 s. The spectra were collected with a data pitch of 0.1 nm, scanning rate (100 nm/min), and a standard sensitivity mode.

g factor analysis

On the basis of above obtained VT-CD/UV-Vis spectra, the *g* factor can be calculated by equation:

$g = \Delta\varepsilon / \varepsilon$

in which $\Delta\varepsilon = [\theta] / 3298$; $[\theta]$ refers to molar ellipticity; and ε refers to molar extinction coefficient.

VT-NMR spectra

Temperature-varied NMR spectra were recorded on a Varian Unity Plus 500 spectrometer (500 MHz). Chemical shifts were denoted in δ values (*ppm*) relative to $CDCl_3$ (^{1}H: δ = 7.26; ^{13}C: δ = 77.00). The temperature of the sample was controlled by a low-temperature cooler connected with a real-time programmed sensor. Measurement was collected after the real-time temperature was fixed around the target temperature (± 1°C) at least 2 min.

ATR IR spectra

All the ATR IR spectra were collected on a Perkin-Elmer FT-IR Spectrometer 400. A few drops of sample solution was added onto the surface of sample platform to evaporate the solvents at ambient conditions. The background of the sample platform was corrected. The temperature was room temperature (25°C) if no special indication.

Liquid-phase IR spectra:

All the liquid-phase IR spectra were collected on a Perkin-Elmer FT-IR Spectrometer 400. A demountable liquid cell (Specac Company, UK) with KBr windows and PTFE spacers (thickness = 1 mm) was used to support the solution samples. The background of solvents ($CDCl_3$ and MCH) was corrected. The temperature was room temperature (25°C) if no special indication.

DFT calculations

Computational analysis was employed to optimize the lower energy conformer structures of the ground state minima of AA-L-Ala, MAA-L-Ala and MAA-L-*t*-Leu**.** Due to the dynamic nature of the molecules involved, all the structures were pre-screened using the CREST driver in the xTB software[1] using the semiempirical GFN2-xTB level. The conformers thus obtained were then reoptimized with DFT at ωB97X-D/def2-TZVP level. All DFT optimizations were conducted with the Gaussian 16, Rev B.01 software package.[2] All minima were confirmed to be such due to the absence of imaginary frequencies and the Gibbs free energies obtained after calculating the Hessian were used to sort the conformers. The electronic circular dichroism was calculated at the TD- ωB97X-D/def2-TZVP level over the first 30 singlet transitions. All xyz coordinates of the most stable conformers considered in this work are provided as separate additional file.

Single crystal preparation

The single crystals were prepared by slow evaporation of the solvent at low temperature (0 ~ 4°C). The sample powders were dissolved in diethyl ether to obtain homogeneous yellow solution. Then 1 vol. equivalent amount of heptane was added and mixed. The resulting homogeneous solution was filtrated two times by cotton filter and then transferred into glass vials, which were sealed by para film with a few small holes. Then the vials were placed into a ventilated fridge to evaporate the solvents slowly. The crystals can be collected after 2 ~ 3 days and then stored in dark for measurement.

X-ray single crystal analysis

A single crystal sample was mounted on top of a cryoloop and transferred into the cold nitrogen stream (100 K) of a Bruker-AXS D8 Venture diffractometer. Data collection and reduction was performed using the Bruker software suite APEX3.[3] The final unit cell was obtained from the xyz centroids of 9824 reflections after integration. A multiscan absorption correction was applied, based on the intensities of symmetry-related reflections measured at different angular settings (SADABS). The structures were solved by direct methods using SHELXT.[4] and refinement of the structure was performed using SHLELXL.[5] The hydrogen atoms were generated by geometrical considerations, constrained to idealized geometries and allowed to ride on their carrier atoms with an isotropic displacement parameter related to the equivalent displacement parameter of their carrier atoms.

Synthesis methods

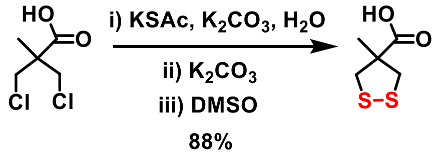

In a typical experiment, 3-chloro-2-(chloromethyl)-2-methylpropanoic acid (6.8 g, 40 mmol) was dissolved in the 80 mL aqueous solution of K_2CO_3 (5.5 g, 40 mmol). Then potassium thioacetate (9.1 g, 80 mmol) dissolved in 40 mL water was added. The mixture was stirred under reflux overnight. Then K_2CO_3 (16.5 g, 120 mmol) powder was slowly added into the reaction mixture and further stirred under reflux overnight. The final oxidation step was performed by adding 3 mL dimethylsulfoxide into the mixture and heated at 85°C under air flow for 2 h. The reaction mixture was then cooled down by ice-water bath and acidified by HCl (aq) (pH = 3). The yellow powder

of MAA was precipitated, separated by filtration, washed by water, and dried in vacuum. The crude powder was used for the next amidation step without further purification.

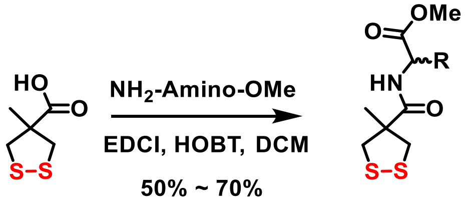

General method: The amidation reaction of MAA was performed by a typical procedure. In a typical experiment, MAA (1 eq) was dissolved in CH_2Cl_2 (50 mL) in flask, forming a homogeneous bright yellow solution. The solution was cooled in ice bath and then EDCI (1.2 eq) and HOBT (1.2 eq) were added and completely dissolved under continuous stirring. After stirring for 15 min, amino acid methyl ester hydrochloride salt (1.2 eq) was deprotonated by DIPEA (1.2 eq) in CH_2Cl_2, which was dropwise added into the mixture solution. Then the reaction solution was stirred at room temperature for 2 ~ 5 h. After confirming the full conversion of reactants by TLC tracing, the mixture solution was washed by 1 M HCl (aq) (50 mL × 3), H_2O (50 mL × 1), saturated $NaHCO_3$ (50 mL × 3), and brine (50 mL × 1). The organic phase was dried by anhydrous Na_2SO_4, and then purified by flash chromatography (SiO_2, pentane/ethyl acetate = 10 : 1 to 4 : 1). The products were collected by evaporating the solvents under vacuum, affording yellow oil or powders (yield = 50% ~ 70%), which can be stored in dark for several months.

MAA-L-Ala: Yellow powder.
^{1}H NMR ($CDCl_3$, 500 MHz, 233 K, ppm): δ = 6.64 (d, J = 7.0 Hz, 1H), 4.53 (m, 1H), 3.76 (s, 3H), 3.55 (dd, J_1 = 12.0 Hz, J_2 = 12.5 Hz, 2H), 3.03 (dd, J_1 = 6 Hz, J_2 = 5.5 Hz, 2H), 1.43 (s, 3H), 1.41 (s, 3H).
^{13}C NMR ($CDCl_3$, 101 MHz, 298 K, ppm) δ = 174.4, 173.4, 77.32 77.0, 76.7, 56.4, 52.5, 50.2, 50.1, 48.4, 23.8, 18.1.
HR-MS (ESI) (m/z): [M + H^+] found: 250.0567; Calculated: 250.0566.

MAA-L-Leu: Yellow powder.
^{1}H NMR ($CDCl_3$, 500 MHz, 298 K, ppm) δ 6.33 (d, J = 8.1 Hz, 1H), 4.59 (s, 1H), 3.74 (s, 3H), 3.63 – 3.43 (m, 2H), 3.00 (dd, J_1 = 12.0 Hz, J_2 = 5.1 Hz, 2H), 1.66 (s, 2H), 1.57 (s, 2H), 1.45 (s, 3H), 0.95 (dd, J_1 = 6.3 Hz, J_2 = 2.6 Hz, 7H).
^{13}C NMR ($CDCl_3$, 101 MHz, 298 K, ppm): δ = 174.7, 173.4, 77.3, 77.0, 76.7, 56.4, 52.3, 51.1, 50.4, 50.1, 41.2, 25.01, 23.8, 22.8, 21.9.
HR-MS (ESI) (m/z): [M + Na^+] found: 314.0859; Calculated: 314.0855.

MAA-L-Val: Yellow oil.
^{1}H NMR ($CDCl_3$, 400 MHz, 298 K, ppm): δ = 6.48 (d, J = 8.4 Hz, 1H), 4.53 (dd, J_1 = 4.8 Hz, J_2 = 4.8 Hz, 1H), 3.72 (s, 3H), 3.56 (dd, J_1 = 12.0 Hz, J_2 = 11.6 Hz, 2H), 3.02 (dd, J_1 = 2.0 Hz, J_2 = 2.0 Hz, 2H), 3.04~ 3.00 (dd, J_1 = 12 Hz, J_1 = 7.5 Hz, 2H), 2.18 (m, 1H), 1.44 (s, 3H), 0.94 (dd, J_1 = 6.8 Hz, J_2 = 6.8 Hz, 6H).
^{13}C NMR ($CDCl_3$, 101 MHz, 298 K, ppm): δ = 174.7, 172.3, 77.3, 77.0, 76.7, 57.3, 56.6, 52.1, 50.3, 50.0, 31.0, 23.7, 19.1, 17.7.
HR-MS (ESI) (m/z): [M + Na^+] found: 300.0702; Calculated: 300.0699.

MAA-L-i-Leu: Yellow oil.
^{1}H NMR ($CDCl_3$, 500 MHz, 293 K, ppm) δ 6.52 (d, J = 7.7 Hz, 1H), 4.58 (s, 1H), 3.74 (s, 3H), 3.63 – 3.42 (m, 2H), 3.01 (d, J = 11.7 Hz, 2H), 1.94 (s, 1H), 1.46 (s, 4H), 1.17 (s, 1H), 0.92 (d, J = 7.1 Hz, 6H).
^{13}C NMR ($CDCl_3$, 101 MHz, 298 K, ppm) δ = 174.6, 172.3, 77.3, 77.0, 76.7, 56.7, 56.5, 52.1, 50.4, 50.0, 37.6, 25.28, 23.7, 15.6, 11.5.
HR-MS (ESI) (m/z): [M + Na^+] found: 314.0861; Calculated: 314.0855.

MAA-L-t-Leu: Yellow powder.
^{1}H NMR ($CDCl_3$, 400 MHz, 293 K, ppm): δ = 6.57 (d, J = 7.2 Hz, 1H), 4.37 (d, J = 7.2 Hz, 1H), 3.69 (s, 3H), 3.49 (dd, J_1 = 10.0 Hz, J_2 = 9.6 Hz, 2H), 3.00 (dd, J_1 = 9.6 Hz, J_2 = 10 Hz, 2H), 1.42 (s, 3H), 0.95 (s, 3H)
^{13}C NMR ($CDCl_3$, 101 MHz, 298 K, ppm): δ = 174.4, 171.9, 77.3, 77.0, 76.7, 60.4, 56.6, 51.8, 50.4, 50.0, 34.6, 26.7, 23.7.
HR-MS (ESI) (m/z): [M + H^+] found: 292.1040; Calculated: 292.1036.

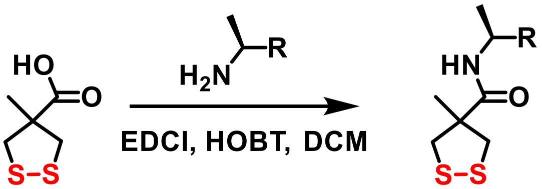

General method: In a typical experiment, MAA (1 eq) was dissolved in CH_2Cl_2 (50 mL) in flask, forming a homogeneous bright yellow solution. The solution was cooled in ice bath and then EDCI (1.2 eq) and HOBT (1.2 eq) were added and completely dissolved under continuous stirring. After stirring for 15 min, the chiral amines (1.5 eq) were dissolved in CH_2Cl_2, which was dropwise added into the mixture solution. Then the reaction solution was stirred at room temperature for 2 ~ 5 h. After confirming the full conversion of reactants by TLC tracing, the mixture solution was washed by 1 M HCl (aq) (50 mL × 3), H_2O (50 mL × 1), saturated $NaHCO_3$ (50 mL × 3), and brine (50 mL × 1). The organic phase was dried by anhydrous Na_2SO_4, and then purified by flash chromatography (SiO_2, pentane/ethyl acetate = 20 : 1 to 5 : 1). The products were collected by evaporating the solvents under vacuum, affording yellow solids (yield = 60% ~ 80%), which can be stored in dark for several months.

MAA-R-Phe: Yellow powder.
^{1}H NMR (d_6-DMSO, 400 MHz, 298 K, ppm): δ = 8.14 (d, J = 7.6 Hz, 1H), 7.28 (d, J = 4.4 Hz, 4H), 7.18 (m, 1H), 4.93 (m, 1H), 3.59 (dd, J_1 = 7.6 Hz, J_2 = 7.2 Hz, 2H), 3.01 (dd, J_1 = 5.2 Hz, J_2 = 5.6 Hz, 2H), 1.36 (s, 3H), 1.34 (d, J = 7.6 Hz, 3H).
^{13}C NMR (d_6-DMSO, 101 MHz, 298 K, ppm): δ = 172.2, 144.8, 128.3, 126.6, 125.9, 57.5, 48.4, 47.1, 47.0, 23.7, 22.2.
HR-MS (ESI) (m/z): [M + Na^+] found: 290.0647; Calculated: 290.0644.

MAA-R-CH: Yellow powder.
^{1}H NMR ($CDCl_3$, 500 MHz, 293 K, ppm): δ = 5.83 (s, 1H), 3.86 (m, 1H), 3.49 (dd, J_1 = 11.5 Hz, J_2 = 8.5 Hz, 2H), 3.01 (d, J = 11.5 Hz, 2H), 1.77 ~ 1.71 (m, 3H), 1.68 (d, J = 12 Hz, 2H), 1.45 (s, 3H), 1.34 (m, 1H), 1.21 (m, 2H), 1.13 ~ 1.08 (m, 4H), 0.99 (m, 2H).

^{13}C NMR ($CDCl_3$, 101 MHz, 298 K, ppm): δ = 173.7, 77.3, 77.0, 76.7, 56.6, 50.1, 49.8, 43.0, 29.2, 28.9, 26.4, 26.16, 26.15, 23.8, 17.8.
HR-MS (ESI) (m/z): [M + H^+] found: 274.1295; Calculated: 274.1294.

MAA-R-Butyl: Yellow powder.
^1H NMR ($CDCl_3$, 500 MHz, 298 K, ppm) δ 5.76 (s, 1H), 3.92 (s, 1H), 3.47 (dd, J_1 = 11.8 Hz, J_2 = 5.1 Hz, 2H), 2.99 (d, J = 11.8 Hz, 2H), 1.44 (s, 5H), 1.13 (d, J = 6.6 Hz, 3H), 0.91 (t, J = 7.4 Hz, 3H).
^{13}C NMR ($CDCl_3$, 101 MHz, 298 K, ppm): δ = 173.6, 56.6, 49.5, 49.4, 46.9, 29.3, 23.7, 20.8, 10.2.
HR-MS (ESI) (m/z): [M + H^+] found: 220.0827; Calculated: 220.0824.

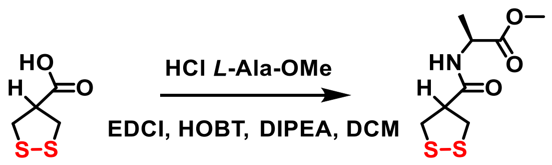

AA-L-Ala: Asparagusic acid (AA) (1.06 g; 10 mmol) was dissolved in CH_2Cl_2 (50 mL) in flask, forming a homogeneous bright yellow solution. The solution was cooled in ice bath and then EDCI (1.5 eq) and HOBT (1.5 eq) were added and completely dissolved under continuous stirring. After stirring for 15 min, L-Alanine methyl ester hydrochloride salt (2.06 g; 15 mmol) was deprotonated by DIPEA (2.6 mL; 15 mmol) in CH_2Cl_2, which was dropwise added into the mixture solution. Then the reaction solution was stirred at room temperature overnight. After confirming the full conversion of reactants by TLC tracing, the mixture solution was washed by 1 M HCl (aq) (30 mL × 3), H_2O (30 mL × 1), saturated $NaHCO_3$ (30 mL × 3), and brine (30 mL × 1). The organic phase was dried by anhydrous Na_2SO_4, and then purified by flash chromatography (SiO_2, CH_2Cl_2/methanol = 200 : 0.25 to 200 : 1). The products were collected by evaporating the solvents under vacuum, affording colorless needle crystals (1.32 g; yield = 56%), which can be stored in dark for several months.
^1H NMR ($CDCl_3$, 500 MHz, 293 K, ppm): δ = 6.30 (s, 1H), 4.59 (m, 1H), 3.76 (s, 3H), 3.39 (m, 4H), 3.33 (m, 1H), 1.43 (d, J = 7 Hz, 3H).
^{13}C NMR ($CDCl_3$, 101 MHz, 298 K, ppm) δ = 173.3, 171.3, 77.3, 77.0, 76.7, 52.6, 52.1, 48.3, 42.8, 42.7, 18.3.
HR-MS (ESI) (m/z): [M + H^+] found: 236.0411; Calculated: 236.0410.

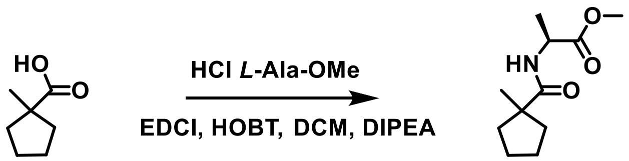

MCP-L-Ala: Methyl cyclopentane acid (MCP) (1.28 g; 10 mmol) was dissolved in CH_2Cl_2 (50 mL) in flask, forming a homogeneous solution. The solution was cooled in ice bath and then EDCI (2.33 g; 15 mmol) and HOBT (2.03 g; 15 mmol) were added and completely dissolved under continuous stirring. After stirring for 15 min, *L*-Alanine methyl ester hydrochloride salt (2.06 g; 15 mmol) was deprotonated by DIPEA (2.6 mL; 15 mmol) in CH_2Cl_2, which was dropwise added into the mixture solution. Then the reaction solution was stirred at room temperature overnight. After confirming the full conversion of reactants by TLC tracing, the mixture solution was washed

by 1 M HCl (aq) (30 mL × 3), H_2O (30 mL × 1), saturated $NaHCO_3$ (30 mL × 3), and brine (30 mL × 1). The organic phase was dried by anhydrous Na_2SO_4, and then purified by flash chromatography (SiO_2, pure CH_2Cl_2 to CH_2Cl_2/methanol = 200 : 1). The products were collected by evaporating the solvents under vacuum, affording colorless oil (1.32 g; yield = 60%).
^{1}H NMR ($CDCl_3$, 500 MHz, 293 K, ppm): δ = 6.13 (s, 1H), 4.56 (m, 1H), 3.73 (s, 1H), 2.01 (m, 2H), 1.68 (s, 4H), 1.48 (m, 2H), 1.38 (d, J = 7 Hz, 3H), 1.22 (s, 3H).
^{13}C NMR ($CDCl_3$, 101 MHz, 298 K, ppm): δ = 178.2, 173.9, 77.3, 77.0, 76.7, 52.4, 49.7, 47.9, 37.68, 37.65, 25.3, 24.8, 18.44, 18.43.
HR-MS (ESI) (m/z): [M + Na^+] found: 236.1259; Calculated: 236.1257.

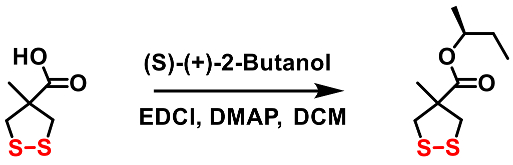

MAA-R-Butyl-ester: MAA (1.64 g; 10 mmol) was dissolved in CH_2Cl_2 (100 mL) in flask, forming a homogeneous bright yellow solution. The solution was cooled in ice bath and then EDCI (2.33 g; 15 mmol) and DMAP (122 mg; 1 mmol) were added and completely dissolved under continuous stirring. After stirring for 5 min, the (S)-(+)-2-butanol (1.0 g; 14 mmol) were dissolved in CH_2Cl_2, which was dropwise added into the mixture solution. Then the reaction solution was stirred at room temperature overnight. After confirming the full conversion of reactants by TLC tracing, the mixture solution was washed by H_2O (50 mL × 3), saturated $NaHCO_3$ (50 mL × 2), and brine (50 mL × 1). The organic phase was dried by anhydrous Na_2SO_4, and then purified by flash chromatography (SiO_2, pentane/CH_2Cl_2 = 10 : 1 to 5 : 1). The products were collected by evaporating the solvents under vacuum, affording yellow oil (0.68 g; yield = 32%), which can be stored in diluted solutions in dark.

Yellow oil
^{1}H NMR ($CDCl_3$, 400 MHz, 298 K, ppm): δ = 4.87 (m, 1H), 3.68 (dd, J_1 = 11.2 Hz, J_2 = 11.2 Hz, 2H), 2.94 (dd, J_1 = 11.6 Hz, J_2 = 11.6 Hz, 2H), 1.606 (m, 2H), 1.47 (s, 3H), 1.23 (d, J = 6.4 Hz, 2H), 0.93 (t, J = 7.2 Hz, 3H).
^{13}C NMR ($CDCl_3$, 101 MHz, 298 K, ppm): δ = 173.9, 77.3, 77.0, 76.7, 73.5, 57.8, 47.8, 47.6, 28.67, 24.1, 19.3, 9.7.
HR-MS (ESI) (m/z): [M + H^+] found: 220.0587; Calculated: 220.0586.

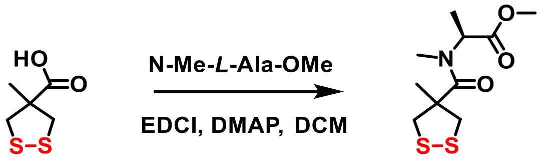

MAA-N-Me-L-Ala: MAA (1.64 g; 10 mmol) was dissolved in CH_2Cl_2 (100 mL) in flask, forming a homogeneous bright yellow solution. The solution was cooled in ice bath and then EDCI (2.33 g; 15 mmol) and DMAP (122 mg; 1 mmol) were added and completely dissolved under continuous stirring. After stirring for 5 min, methyl (2*S*)-2-(methylamino)propanoate (1.0 g; 8.5 mmol) were dissolved in CH_2Cl_2, which was dropwise added into the mixture solution. Then the reaction solution was stirred at room temperature for four days. The conversion of the reaction is very slow possibly due to the steric hinderance. After that the mixture solution was washed by diluted HCl

aqueous solution (0.5 M; 50 mL × 2), saturated $NaHCO_3$ (50 mL × 2), and brine (50 mL × 1). The organic phase was dried by anhydrous Na_2SO_4, and then purified by flash chromatography (SiO_2, CH_2Cl_2/CH_3OH = 400 : 1 to 200 : 1). The products were collected by evaporating the solvents under vacuum, affording yellow oil (~15 mg; yield = ~1%). The freshly prepared compounds are immediately used for spectroscopic characterization.

Yellow oil
^1H NMR ($CDCl_3$, 300 MHz, 298 K, ppm): δ = 4.93 (t, J = 7.2 Hz, 1H), 3.70 (s, 3H), 3.62 (dd, J_1 = 12.0 Hz, J_2 = 11.5 Hz, 3H), 3.05 (d, J = 11.7 Hz, 2H), 1.48 (s, 3H), 1.41 (d, J = 7.5 Hz, 2H).

^{13}C NMR ($CDCl_3$, 151 MHz, 298 K, ppm): δ = 174.23, 171.94, 56.97, 54.53, 52.15, 49.85, 49.18, 33.19, 30.77, 25.30, 14.08, 13.95.

HR-MS (ESI) (m/z): [M + Na^+] found: 286.0546; Calculated: 286.0542.

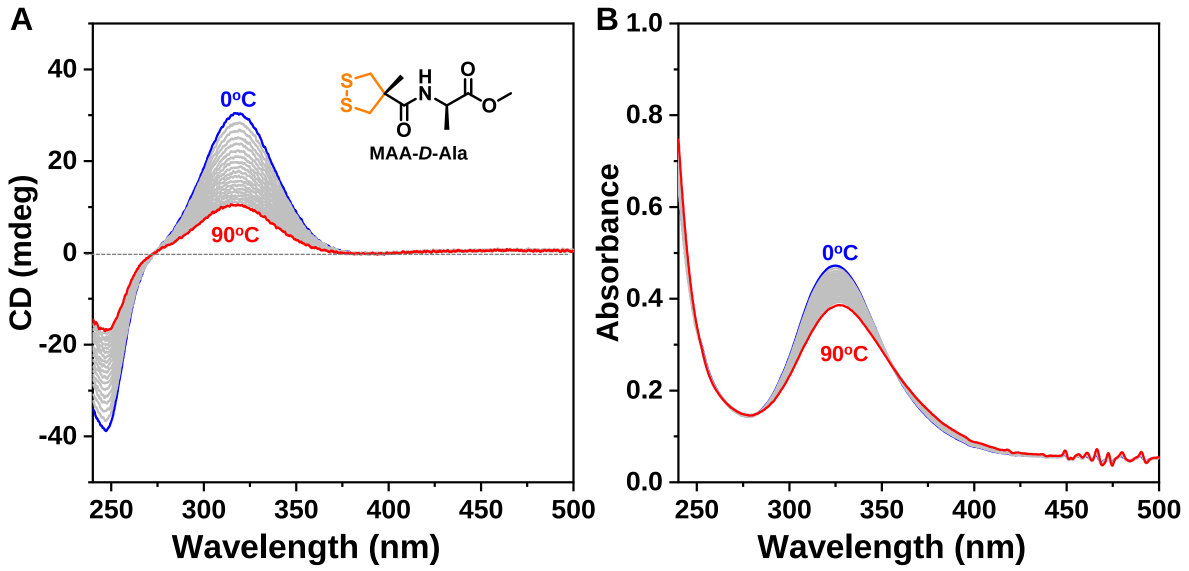

Fig. S1.
Temperature-Varied CD (A) and UV-Vis absorption spectra (B) of MAA-*D*-Ala in MCH (2 mM). Optical path = 10 mm; Temperature region was set up from 273K (blue) to 363K (red).

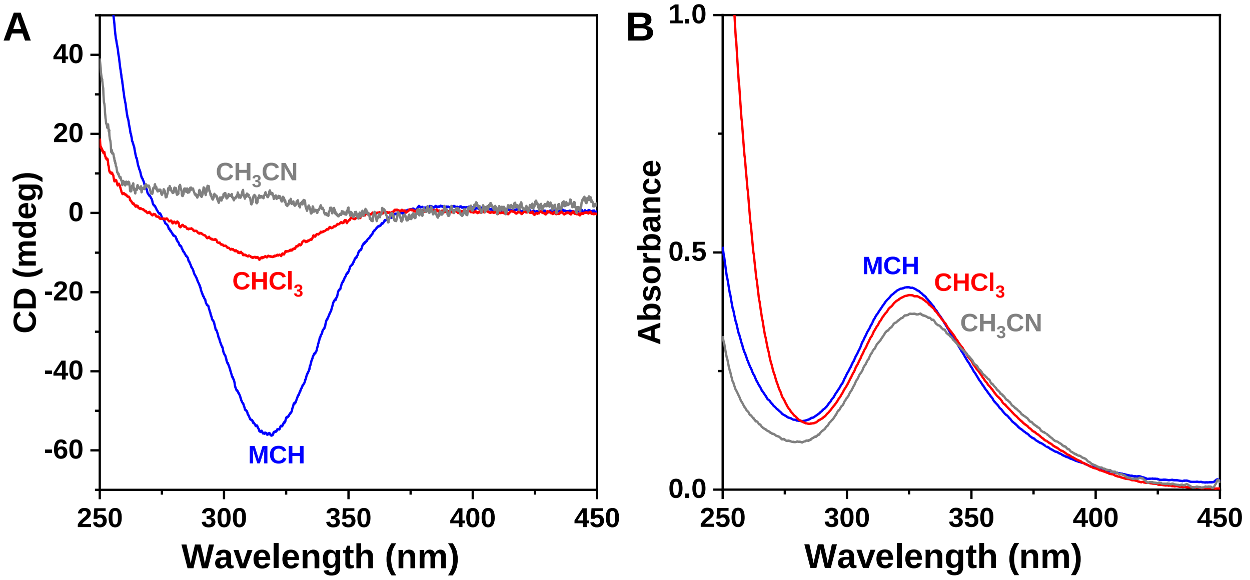

Fig. S2.
Polarity-dependency of the CD spectra (A) and UV-Vis spectra (B) of MAA-*L*-Ala. Optical path = 10 mm. Acetonitrile solution exhibited very low ellipticity in CD spectra, and meanwhile slightly red-shifted absorption band in UV-Vis spectra, indicating the higher conformational freedom of the disulfide five-membered ring in acetonitrile, which enables more metastable and more planar conformers with red-shifted absorption bands.

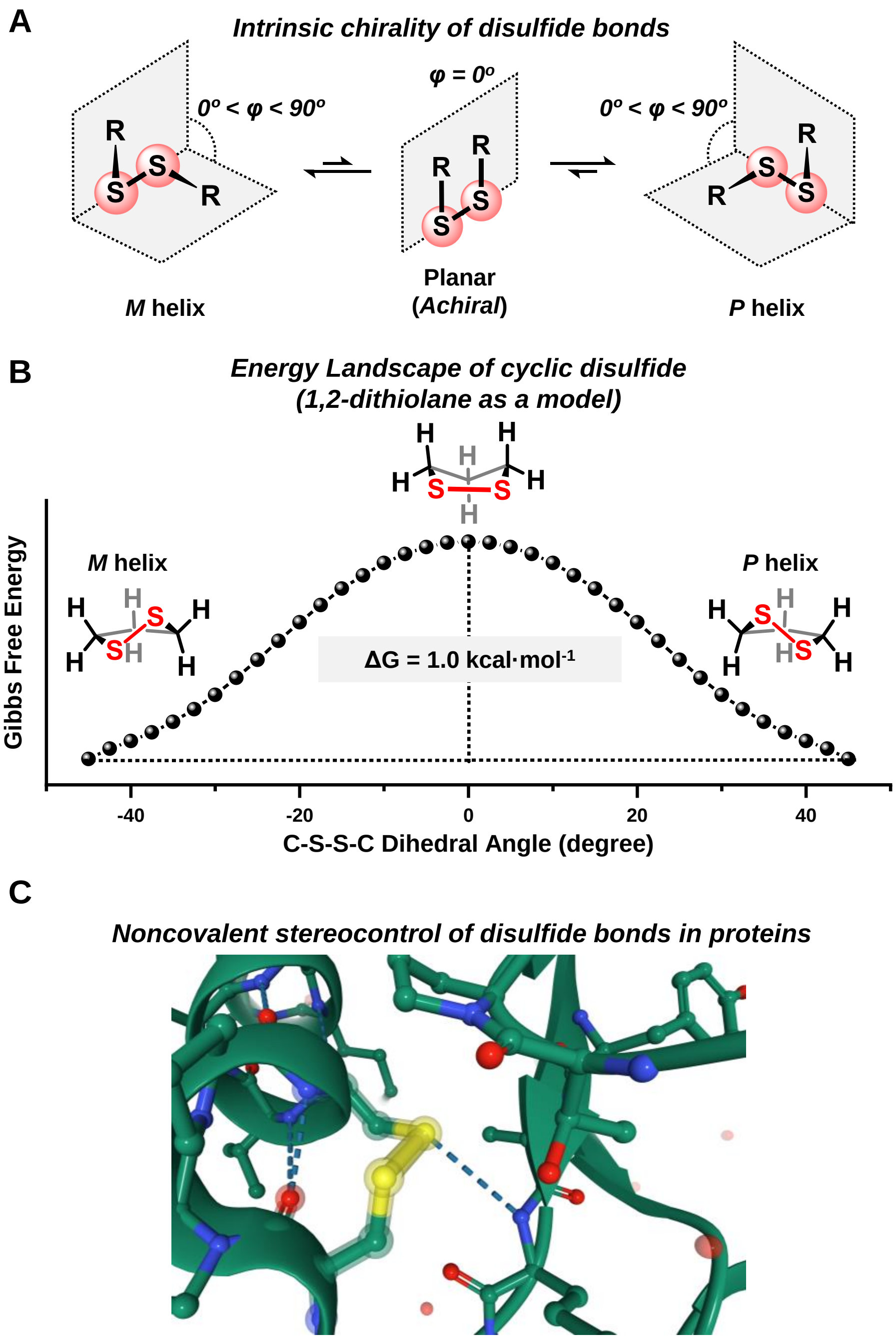

Fig. S3. (**A**) Schematic representation of the intrinsic helicity of disulfide bonds; (**B**) Gibbs free energy analysis of disulfide bonds with different dihedral angles; (**C**) An example of the noncovalent stereocontrol of disulfide bonds in a protein structure obtained by Protein Data Bank (5NYK). The crystal structure of oxidoreductase protein contains a disulfide bond with *P* helicity, in which one of the sulfur atoms forms S-S···H-N hydrogen bonds (H-bond length estimated as 2.77 Å).

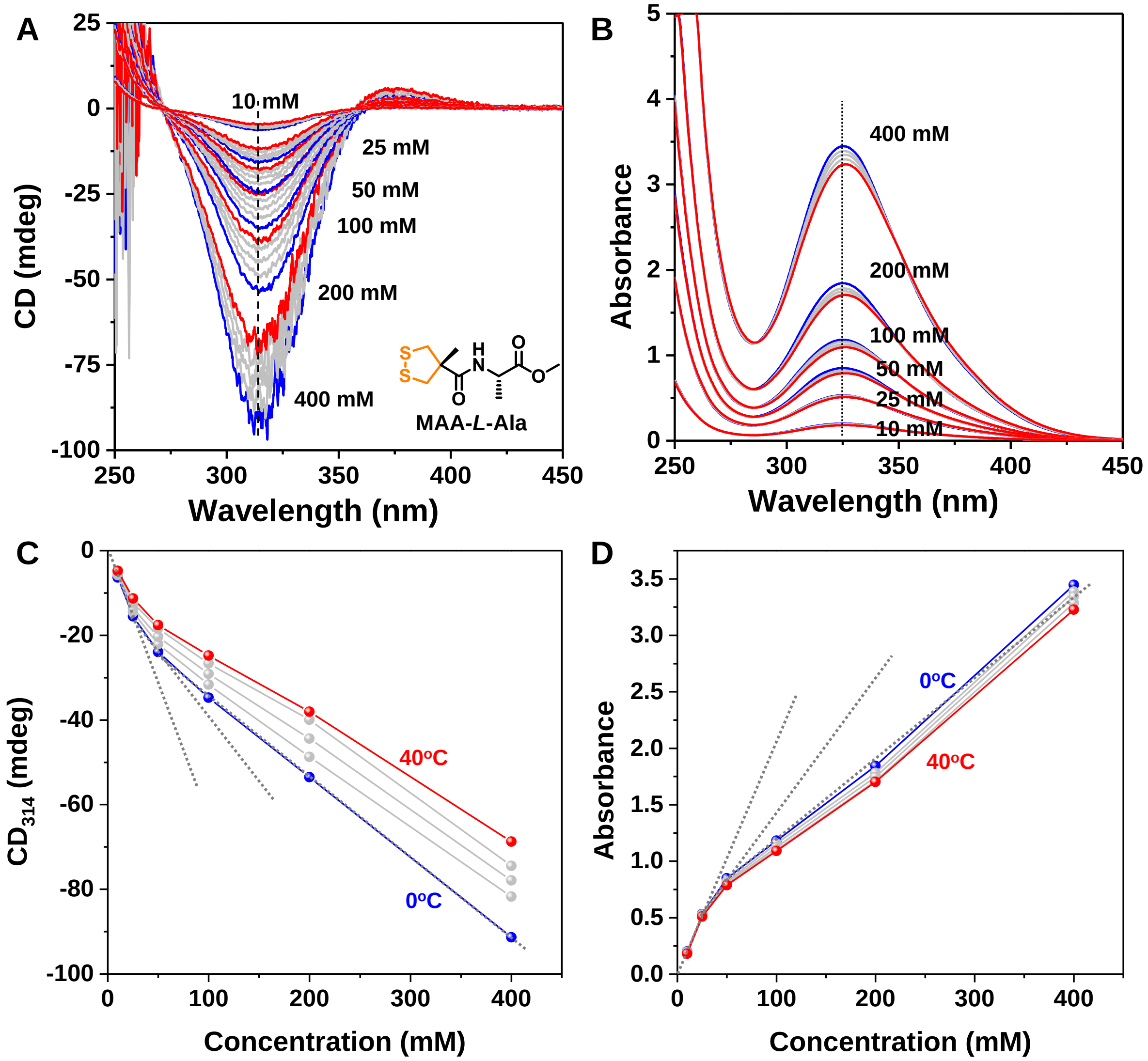

Fig. S4.

Concentration-dependency of the CD spectra of MAA-*L*-Ala. Temperature-Varied CD (A, C) and UV-Vis absorption spectra (B, D) of MAA-*L*-Ala in $CHCl_3$ at varied concentrations (10 mM ~ 400 mM). Optical path = 0.5 mm; Temperature region was set up from 273K (blue) to 313K (red).

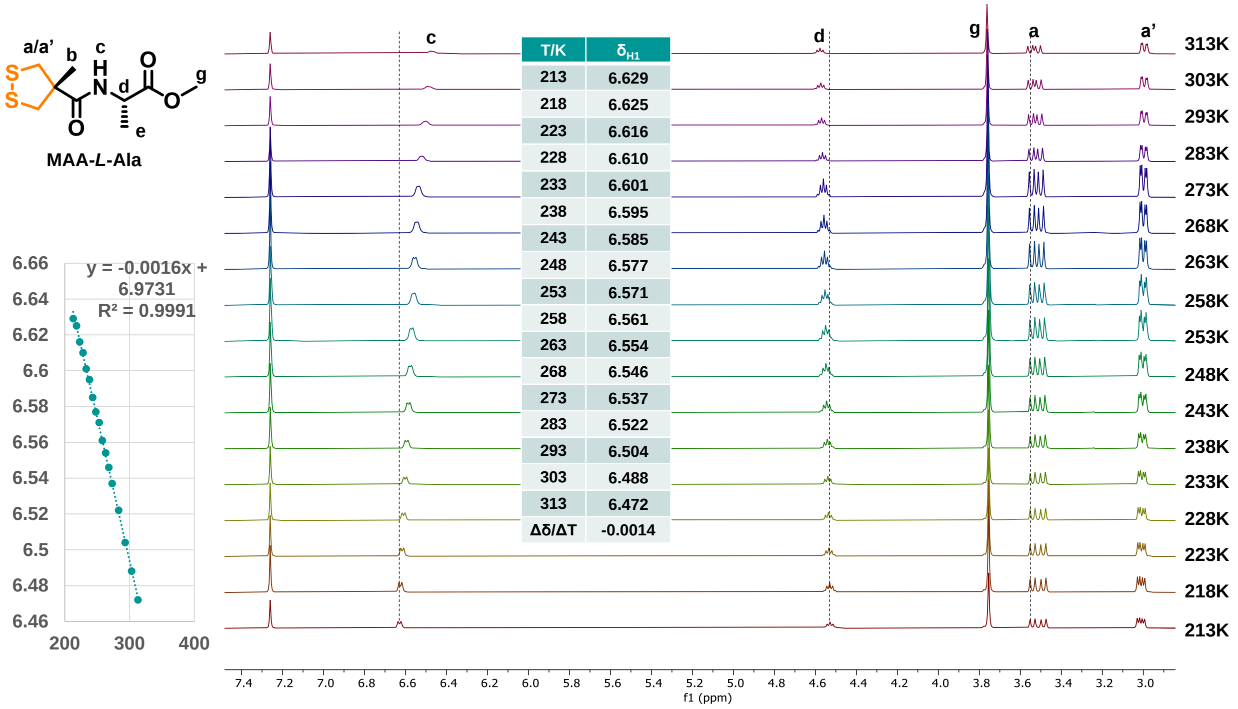

T/K	δ_{H1}
213	6.629
218	6.625
223	6.616
228	6.610
233	6.601
238	6.595
243	6.585
248	6.577
253	6.571
258	6.561
263	6.554
268	6.546
273	6.537
283	6.522
293	6.504
303	6.488
313	6.472
Δδ/ΔT	-0.0014

Fig. S5.

Temperature-varied ^{1}H NMR spectra of MAA-*L*-Ala in $CDCl_3$ (500 MHz, 10 mM). See detailed structural characterization in Fig. S72-S74.

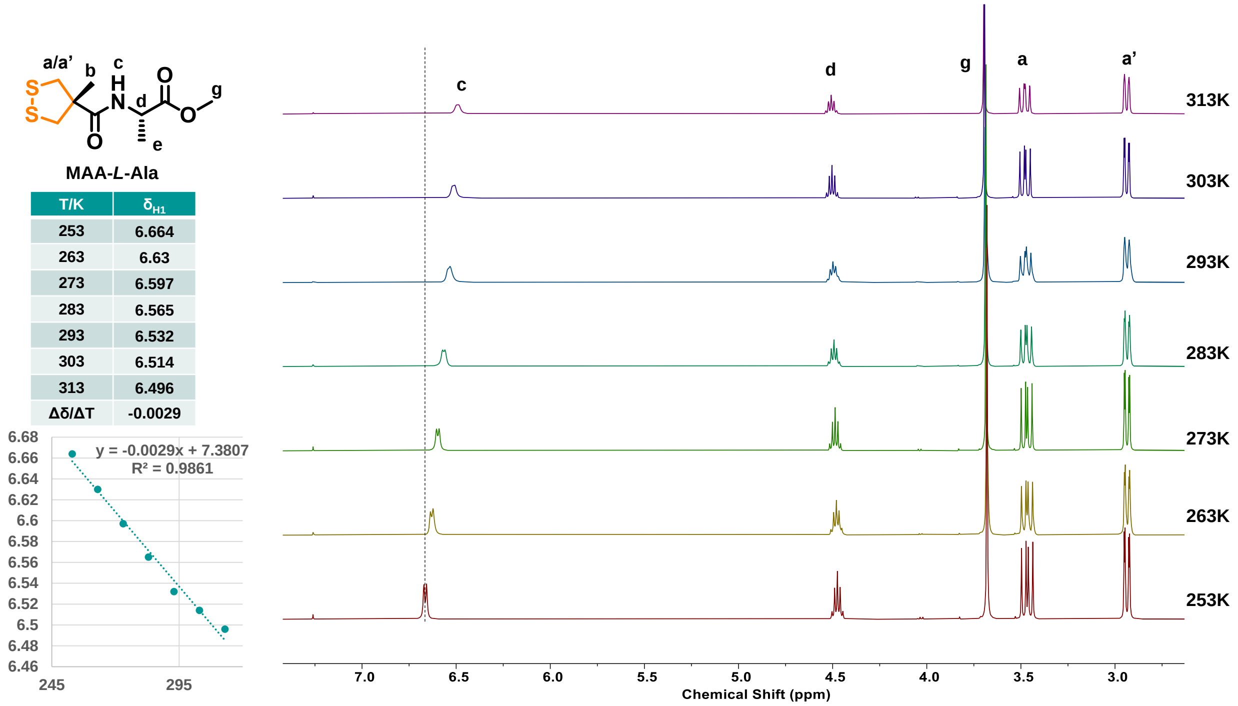

T/K	δ_{H1}
253	6.664
263	6.63
273	6.597
283	6.565
293	6.532
303	6.514
313	6.496
Δδ/ΔT	-0.0029

Fig. S6.

Temperature-varied ^{1}H NMR spectra of MAA-*L*-Ala in $CDCl_3$ (500 MHz, 200 mM).

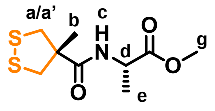

MAA-*L*-Ala

T/K	δ_{H1}
253	6.569
263	6.553
273	6.535
283	6.519
293	6.505
303	6.488
313	6.471
Δδ/ΔT	-0.0016

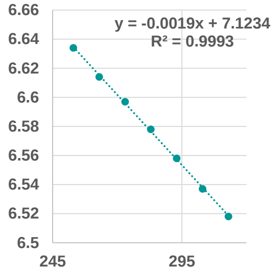

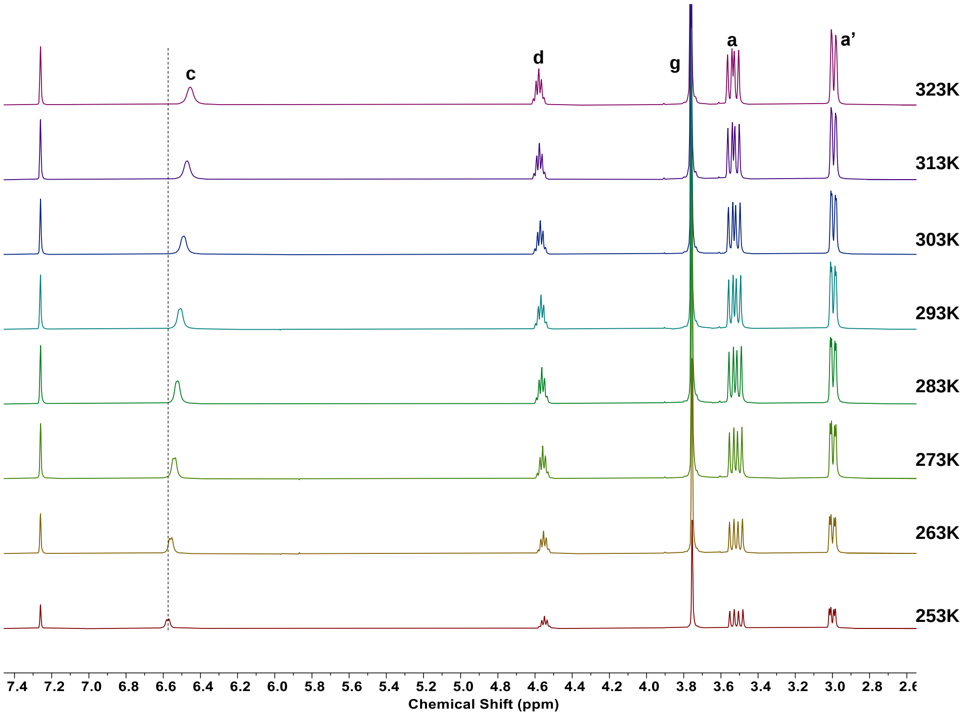

Fig. S7.

Temperature-varied ^{1}H NMR spectra of MAA-*L*-Ala in $CDCl_3$ (500 MHz, 20 mM).

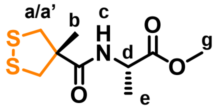

MAA-*L*-Ala

T/K	δ_{H1}
253	6.571
263	6.554
273	6.537
283	6.514
293	6.5
303	6.489
313	6.471
Δδ/ΔT	-0.0017

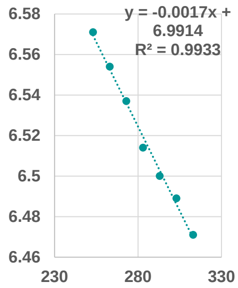

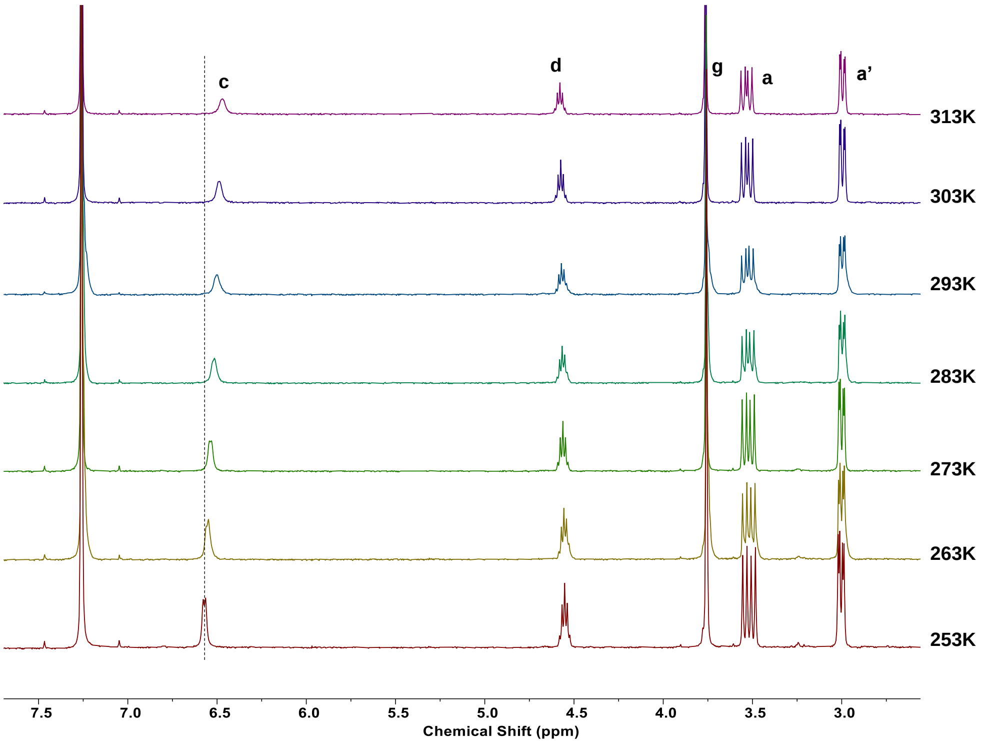

Fig. S8.

Temperature-varied ^{1}H NMR spectra of MAA-*L*-Ala in $CDCl_3$ (500 MHz, 2 mM).

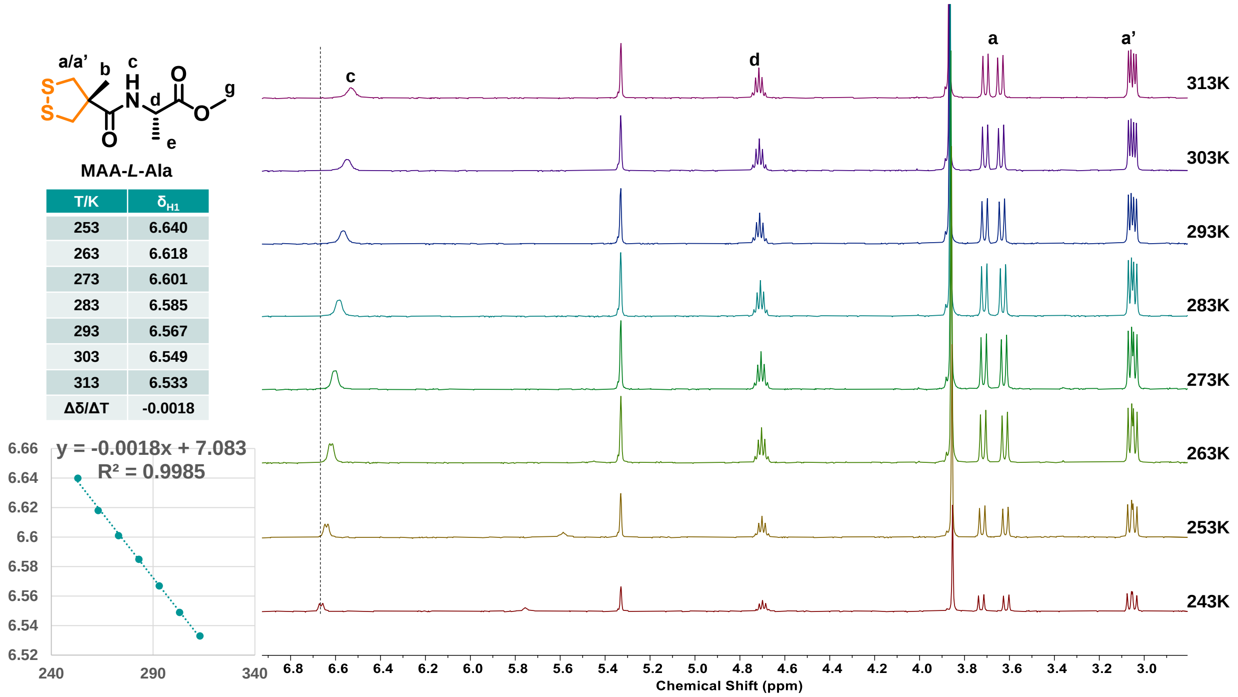

T/K	δ_{H1}
253	6.640
263	6.618
273	6.601
283	6.585
293	6.567
303	6.549
313	6.533
Δδ/ΔT	-0.0018

Fig. S9.

Temperature-varied ^{1}H NMR spectra of MAA-*L*-Ala in d14-MCH (500 MHz, 2 mM).

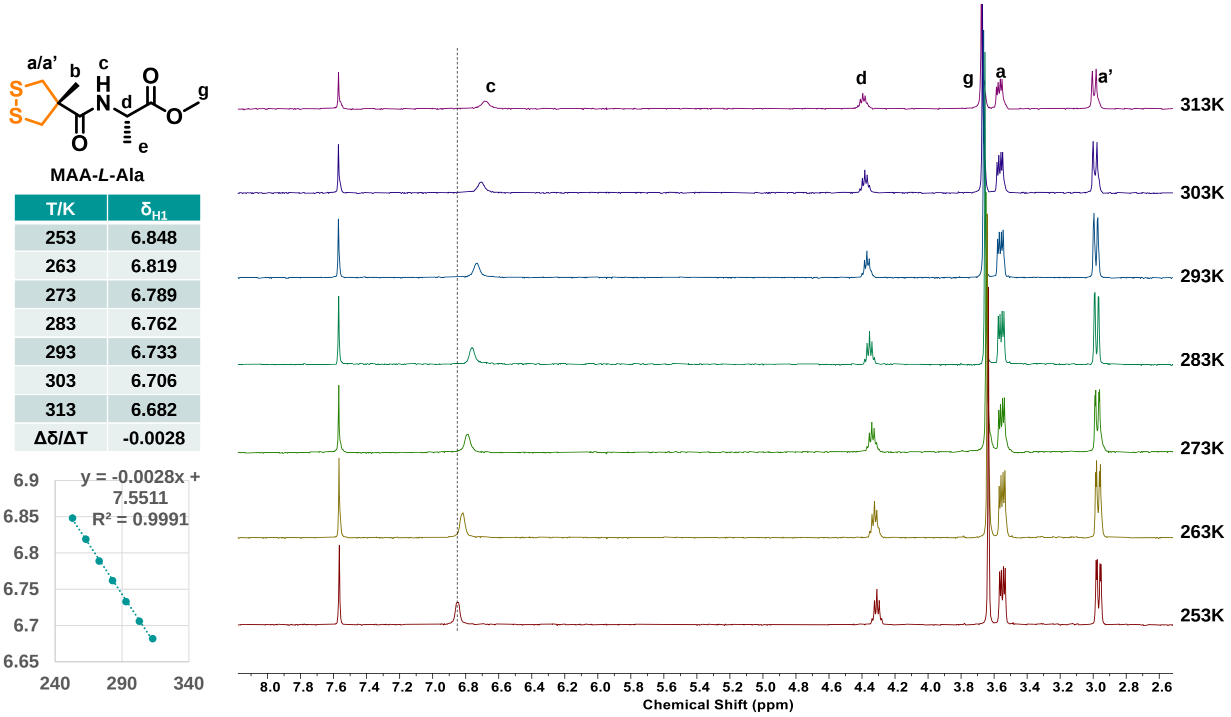

T/K	δ_{H1}
253	6.848
263	6.819
273	6.789
283	6.762
293	6.733
303	6.706
313	6.682
Δδ/ΔT	-0.0028

Fig. S10.

Temperature-varied ^{1}H NMR spectra of MAA-*L*-Ala in CD_3CN (500 MHz, 10 mM).

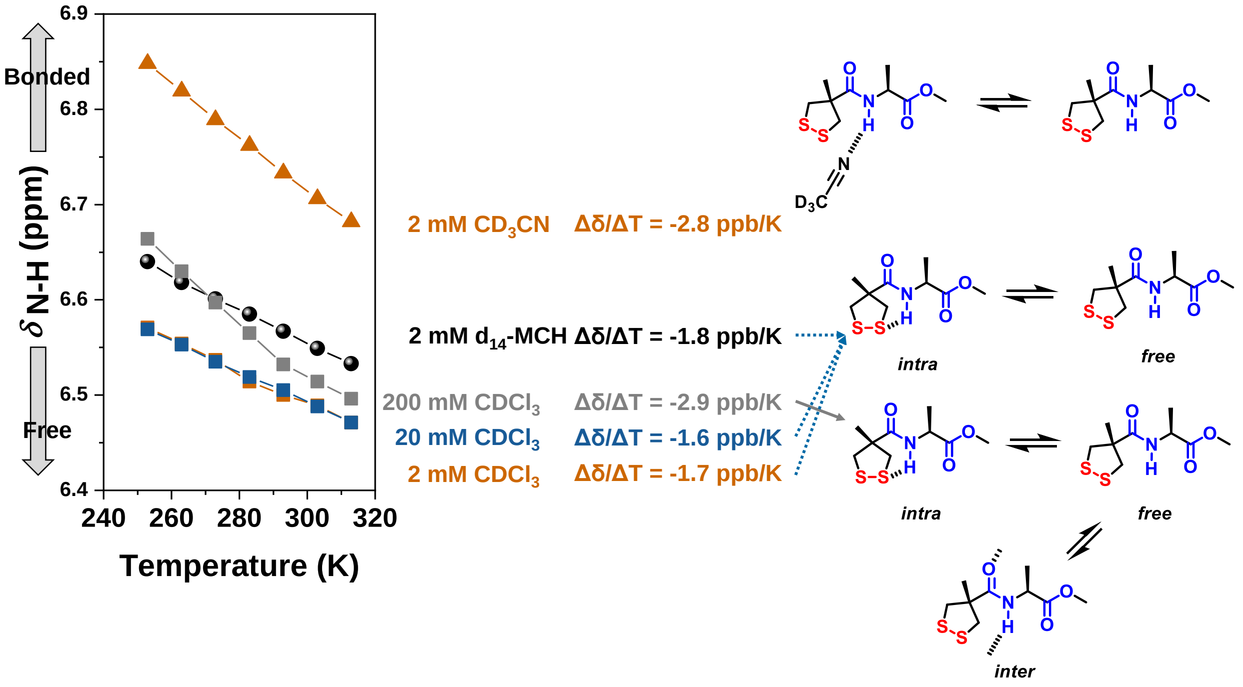

Fig. S11.

Temperature-varied chemical shift ($\delta_{N\text{-}H}$) of MAA-*L*-Ala in different conditions. In $CDCl_3$, the values of $\delta_{N\text{-}H}$ and $\Delta\delta/\Delta T$ remain almost unchanged between 2 mM and 20 mM, indicating the molecularly dissolved status, that is the dynamic equilibrium between intramolecular hydrogen bonded state and unbonded state. When the concentration increases to 200 mM, the values of $\delta_{N\text{-}H}$ and $\Delta\delta/\Delta T$ increase notably, suggesting that intermolecular hydrogen bonds contribute to the temperature-dependent dynamic equilibrium, because the self-assembly process is highly sensitive to temperature change due to the high entropy. With fixed concentration (2 mM), three different solvents are also compared, including CD_3CN, $CDCl_3$, and d_{14}-MCH. Both of the cases in $CDCl_3$, and d_{14}-MCH are similarly dominated by the intramolecular hydrogen bonds, while the CD_3CN solution exhibits most downfield chemical shift and highest value of $\Delta\delta/\Delta T$, which is due to the formation of solvated CN···H-N hydrogen bonds.

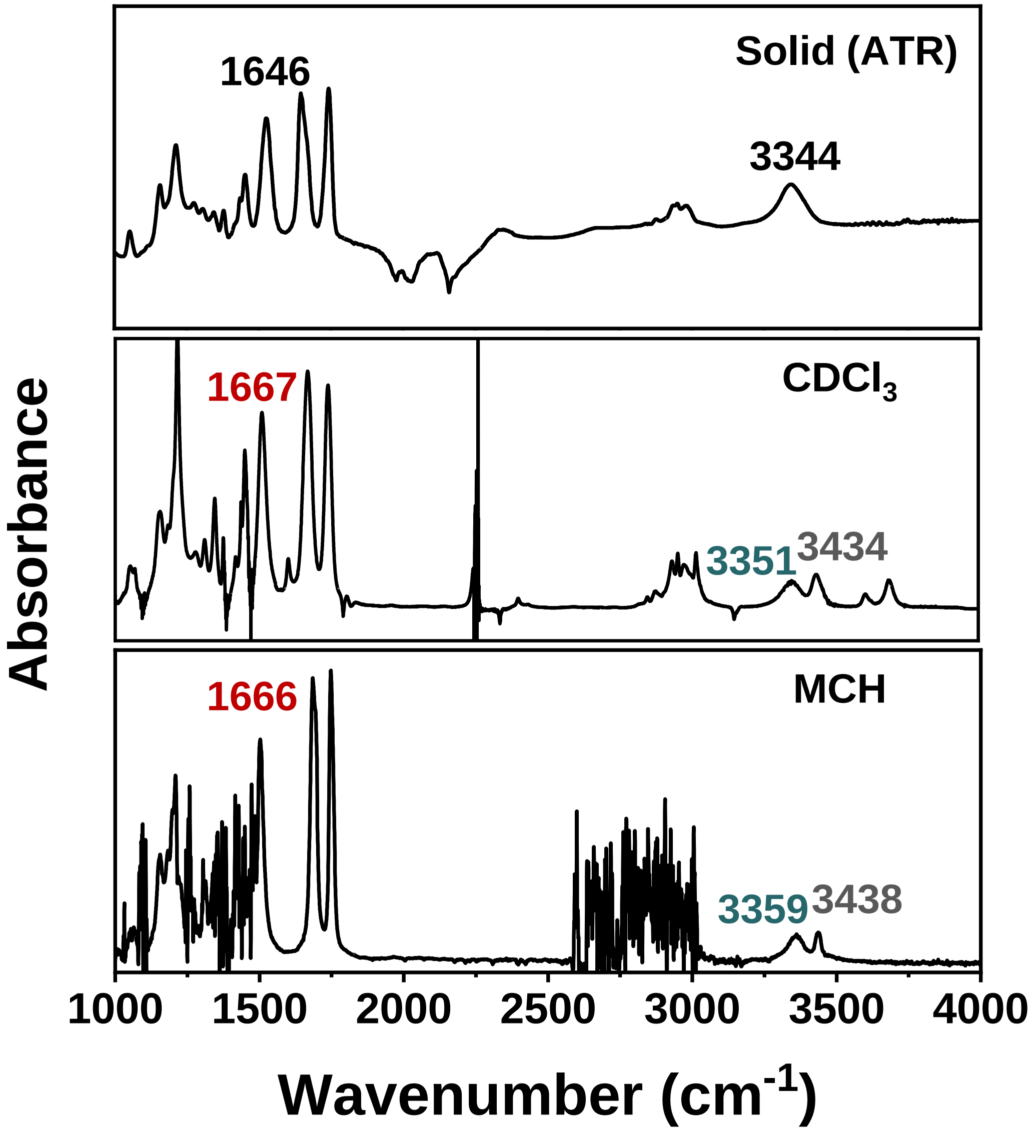

Fig. S12.

IR spectra of MAA-*L*-Ala. (A) ATR-IR spectra of MAA-*L*-Ala solid by evaporating a drop of sample solution; (B) Transmission IR spectra of MAA-*L*-Ala in diluted $CDCl_3$ solution (10 mM); (C) Transmission IR spectra of MAA-*L*-Ala in diluted MCH solution (2 mM).

	View A	View B	Population Percentage	S-S chirality/φ	S-S···H-N H-bond
1	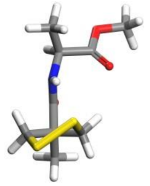	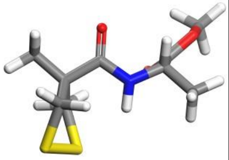	46%	***M*** **chirality** $\varphi_{C\text{-}S\text{-}S\text{-}C}$ = 42°	l = 2.567 Å $\theta_{N\text{-}H\text{-}S}$ = 137°
2	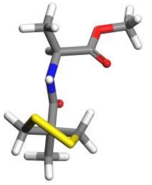	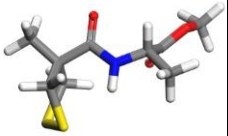	28%	***P*** **chirality** $\varphi_{C\text{-}S\text{-}S\text{-}C}$ = 44°	l = 2.567 Å $\theta_{N\text{-}H\text{-}S}$ = 135°
3	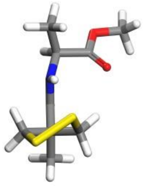	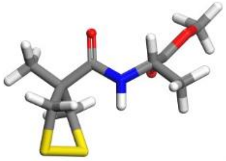	7%	***M*** **chirality** $\varphi_{C\text{-}S\text{-}S\text{-}C}$ = 43°	l = 2.567 Å $\theta_{N\text{-}H\text{-}S}$ = 137°
4	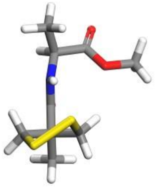	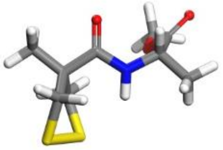	5%	***M*** **chirality** $\varphi_{C\text{-}S\text{-}S\text{-}C}$ = 45°	l = 2.578 Å $\theta_{N\text{-}H\text{-}S}$ = 136°
5	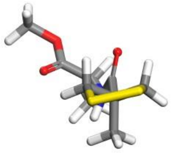	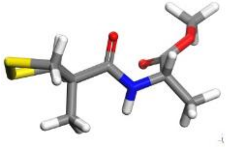	4%	***M*** **chirality** $\varphi_{C\text{-}S\text{-}S\text{-}C}$ = 20°	Not Observed
6	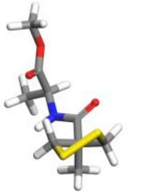	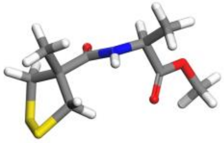	3%	***M*** **chirality** $\varphi_{C\text{-}S\text{-}S\text{-}C}$ = 46°	Not Observed
7	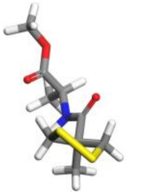	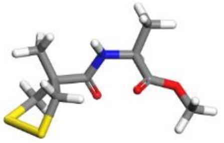	1%	***P*** **chirality** $\varphi_{C\text{-}S\text{-}S\text{-}C}$ = 45°	Not Observed

Fig. S13.

DFT-optimized geometries in vacuo of the low-energy conformers of MAA-*L*-Ala, with their population percentage derived from Boltzmann averaging.

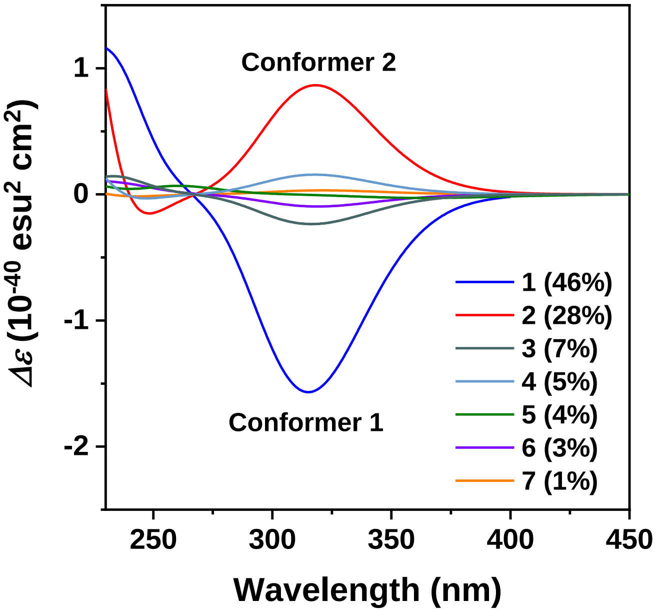

Fig. S14.

Simulated CD spectra of the different conformations of MAA-*L*-Ala as shown in Figure S13 with the intensities scaled using the Boltzmann averaged population.

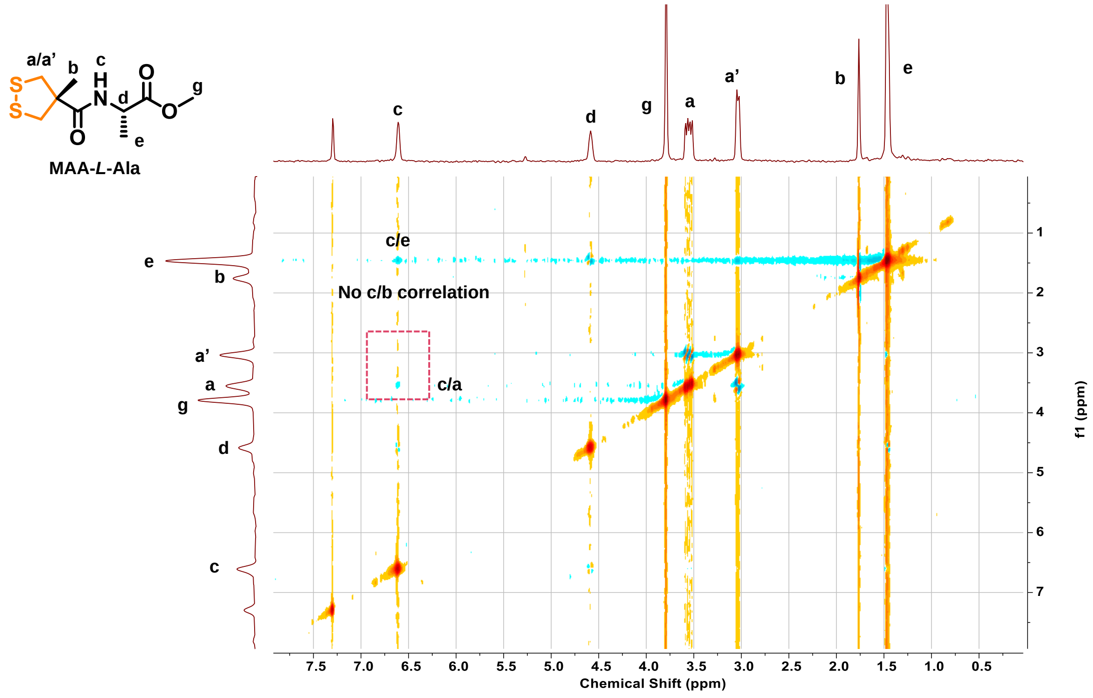

Fig. S15.

2D NOESY analysis of MAA-*L*-Ala. (500 MHz, 253K, 10 mM $CDCl_3$).

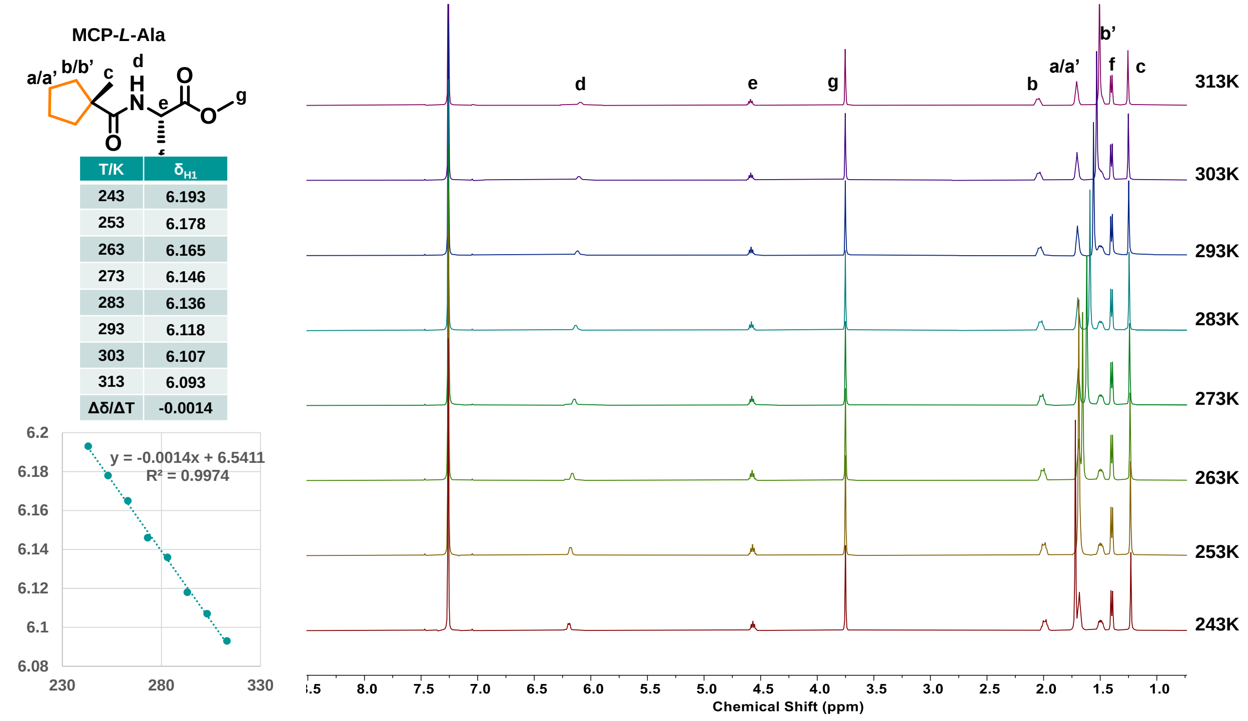

T/K	δ_{H1}
243	6.193
253	6.178
263	6.165
273	6.146
283	6.136
293	6.118
303	6.107
313	6.093
Δδ/ΔT	-0.0014

Fig. S16.

Temperature-varied ^{1}H NMR spectra of MCP-*L*-Ala in $CDCl_3$ (500 MHz, 2 mM). See detailed structural characterization in Fig. S99-S102.

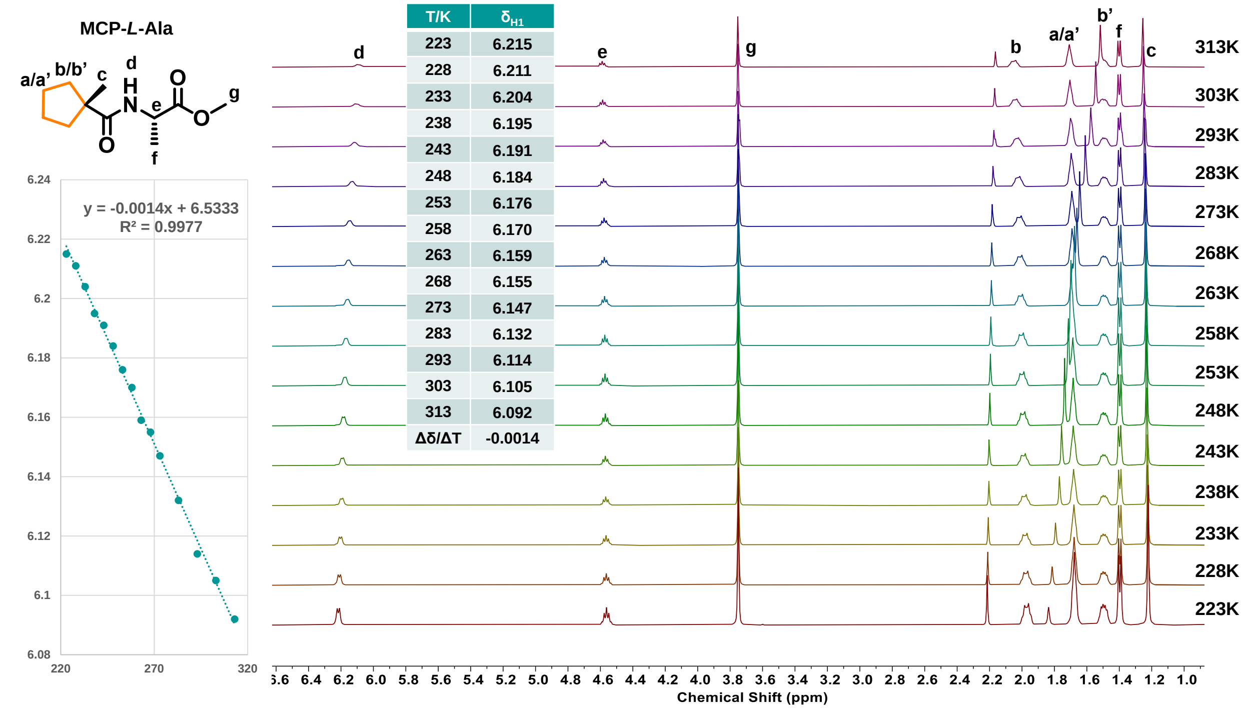

T/K	δ_{H1}
223	6.215
228	6.211
233	6.204
238	6.195
243	6.191
248	6.184
253	6.176
258	6.170
263	6.159
268	6.155
273	6.147
283	6.132
293	6.114
303	6.105
313	6.092
Δδ/ΔT	-0.0014

Fig. S17.

Temperature-varied ^{1}H NMR spectra of MCP-*L*-Ala in $CDCl_3$ (500 MHz, 10 mM).

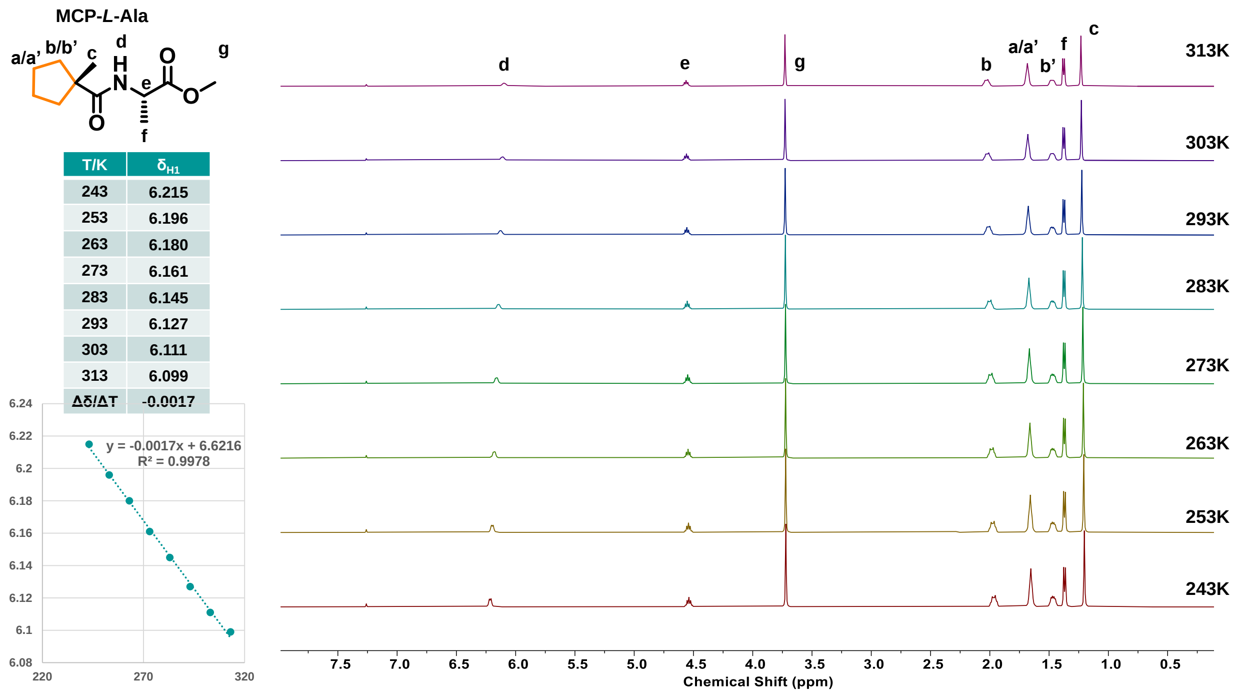

T/K	δ_{H1}
243	6.215
253	6.196
263	6.180
273	6.161
283	6.145
293	6.127
303	6.111
313	6.099
Δδ/ΔT	-0.0017

Fig. S18.

Temperature-varied ^{1}H NMR spectra of MCP-*L*-Ala in $CDCl_3$ (500 MHz, 200 mM).

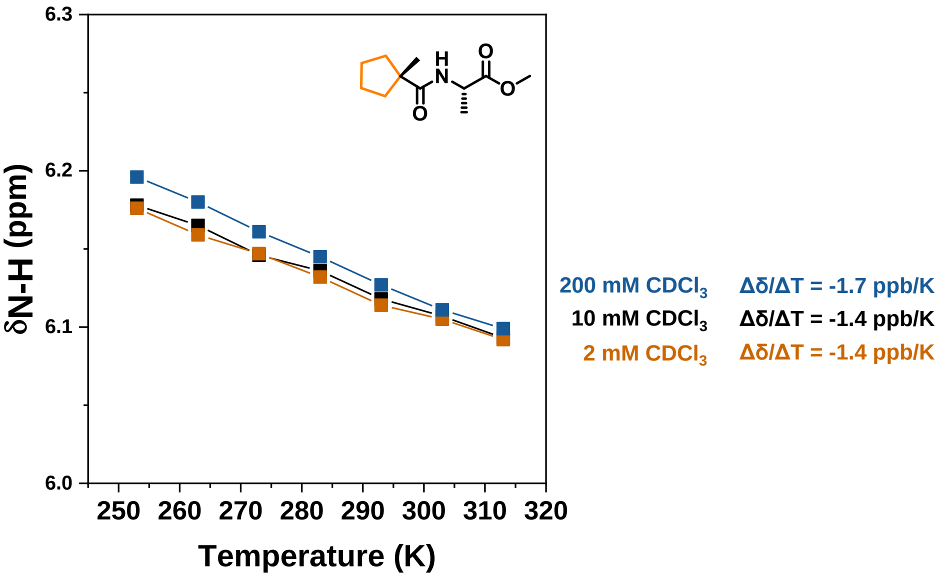

Fig. S19.

Temperature-varied chemical shift ($\delta_{N\text{-}H}$) of MCP-*L*-Ala in different concentrations. The very adjacent $\delta_{N\text{-}H}$ and similar values of Δδ/ΔT at different concentrations indicated the molecularly dissolved nature of MCP-*L*-Ala in diluted $CDCl_3$ solution (10 mM). Meanwhile, compared with MAA-*L*-Ala at same conditions, the $\delta_{N\text{-}H}$ of MCP-*L*-Ala showed more upfield ($|\Delta\delta|_{293K,\ 10\ mM}$ = 0.450 ppm), revealing the more bonded status of MAA-*L*-Ala due to the presence of intramolecular S-S···H-N hydrogen bonds.

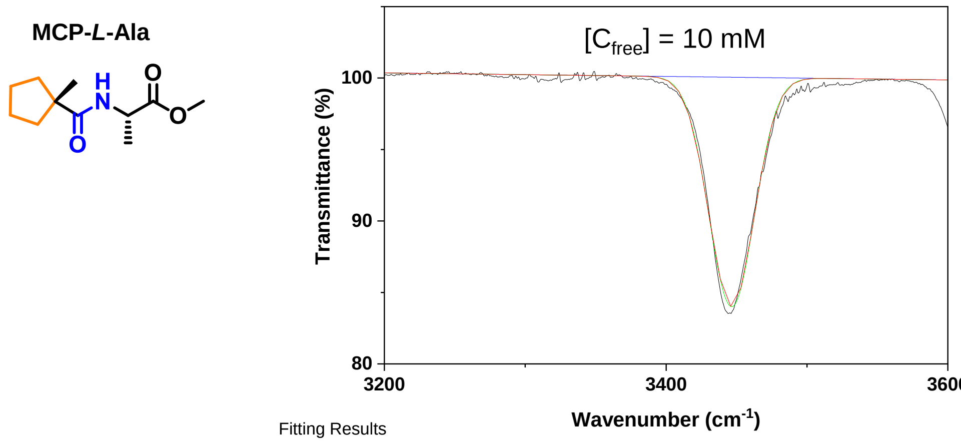

Fitting Results

Peak Index	Peak Type	Area Intg	FWHM	Max Height	Center Grvty	Area IntgP
1	Gaussian	-644.98111	37.75047	-16.05064	3446.54938	-100

Fig. S20.

IR spectra of the amide vibration band (ν_{NH}) of MCP-*L*-Ala in 10 mM $CDCl_3$ solutions at 293K. The integration area was used as a reference light-extinction coefficient to quantify the molar concentrations of free amide protons in MAA-*L*-Ala.

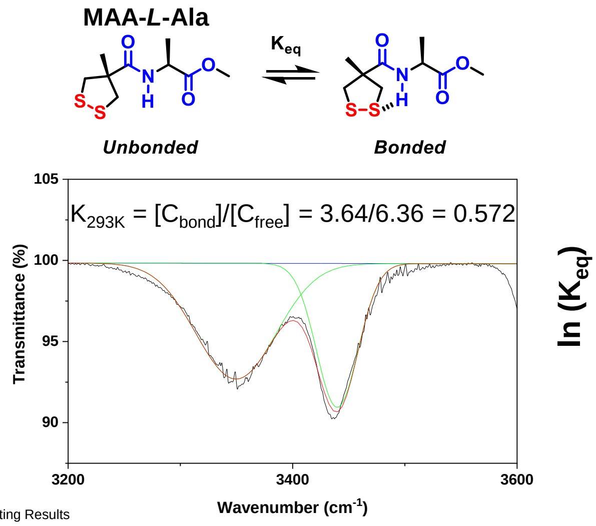

Fitting Results

Peak Index	Peak Type	Area Intg	FWHM	Max Height	Center Grvty	Area IntgP
1	Gaussian	-410.06577	43.47812	-8.86034	3439.75104	-39.07704
2	Gaussian	-639.31191	84.14899	-7.13726	3349.57332	-60.92296

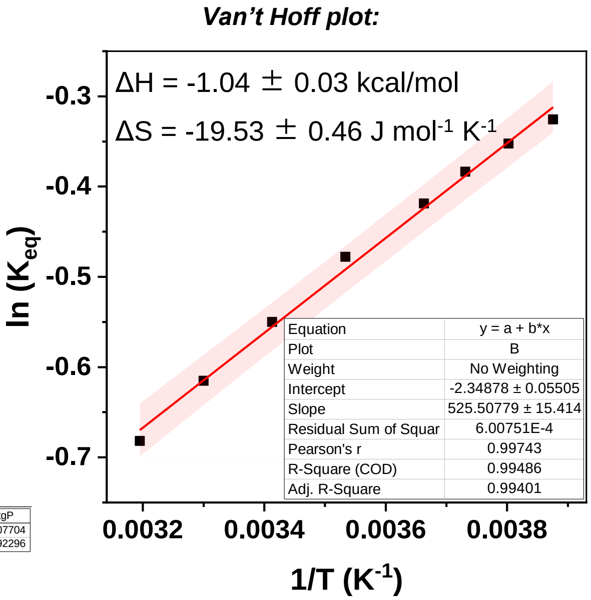

Fig. S21.

IR spectra of the amide vibration band (ν_{NH}) of MAA-*L*-Ala in 10 mM $CDCl_3$ solutions at 293K and the van't Hoff fitting plot according to VT-NMR spectra. The peaks were separated into two major peaks (the H-bonded and the unbonded), whose integration area was further used to calculate the equilibrium constant at 293K (K_{293K}). To evaluate the thermodynamic parameters, van't Hoff plots were fit by combining the IR data and temperature-varied ^1H NMR spectra. The detailed fitting method has been described in Method section. Red band indicates 95% confidence intervals.

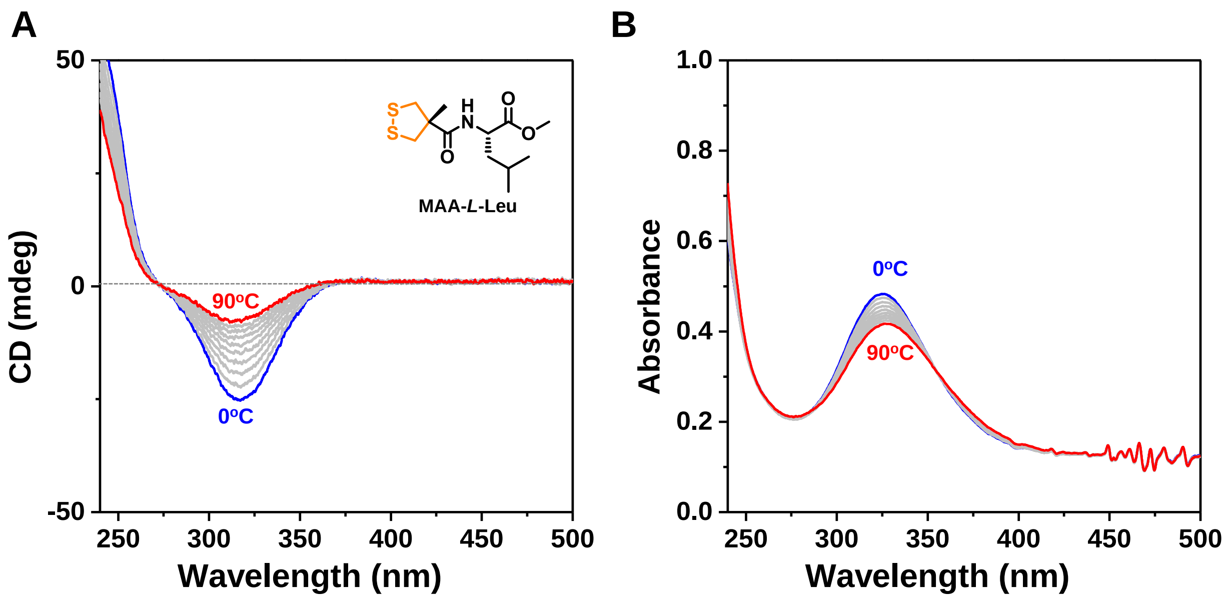

Fig. S22.

Temperature-Varied CD (a) and UV-Vis absorption spectra (b) of MAA-*L*-Leu in MCH (2.1 mM). Optical path = 10 mm; Temperature region was set up from 273K (blue) to 363K (red) with 10K increments.

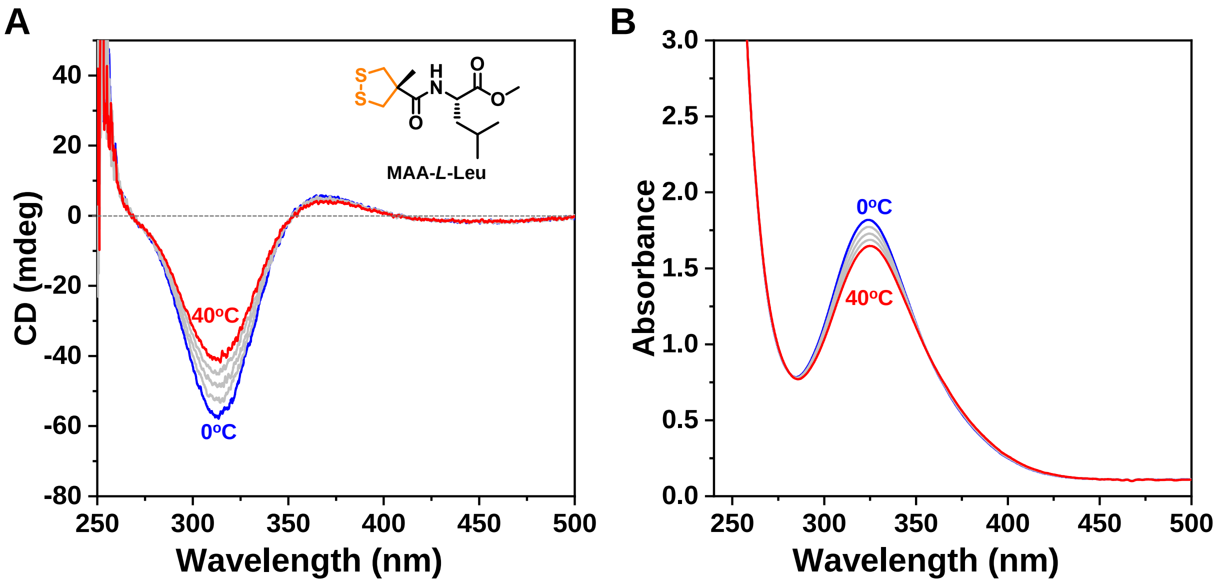

Fig. S23.

Temperature-Varied CD (A) and UV-Vis absorption spectra (B) of MAA-*L*-Leu in $CHCl_3$ (10 mM). Optical path = 10 mm; Temperature region was set up from 273K (blue) to 313K (red) with 10K increments.

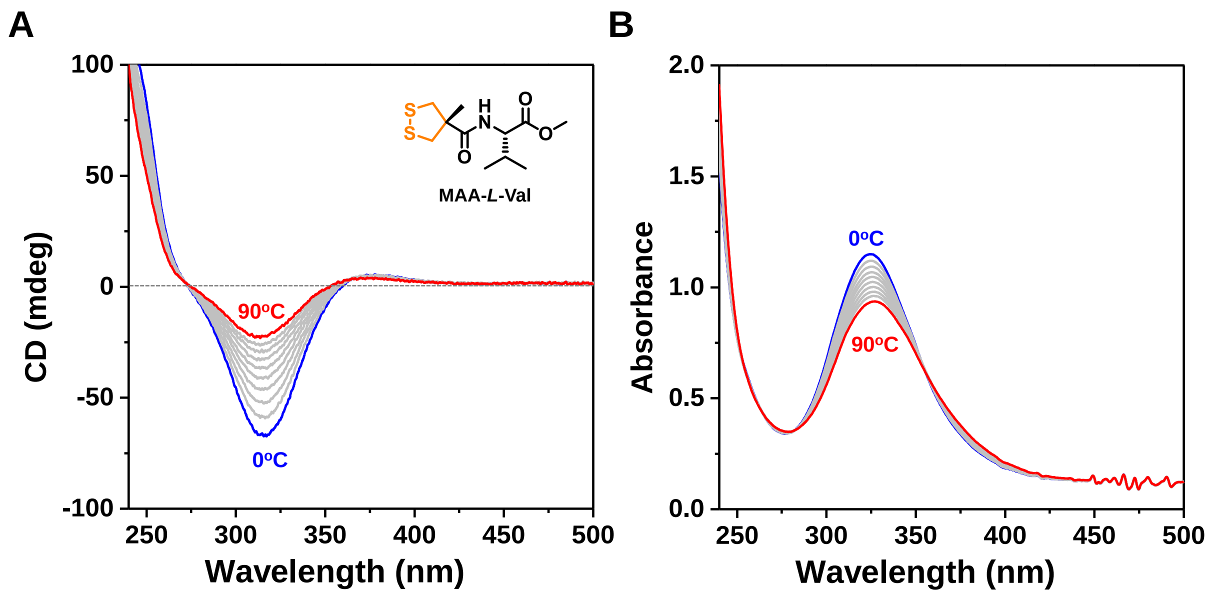

Fig. S24.

Temperature-Varied CD (A) and UV-Vis absorption spectra (B) of MAA-*L*-Val in MCH (5 mM). Optical path = 10 mm; Temperature region was set up from 273K (blue) to 363K (red) with 10K increments.

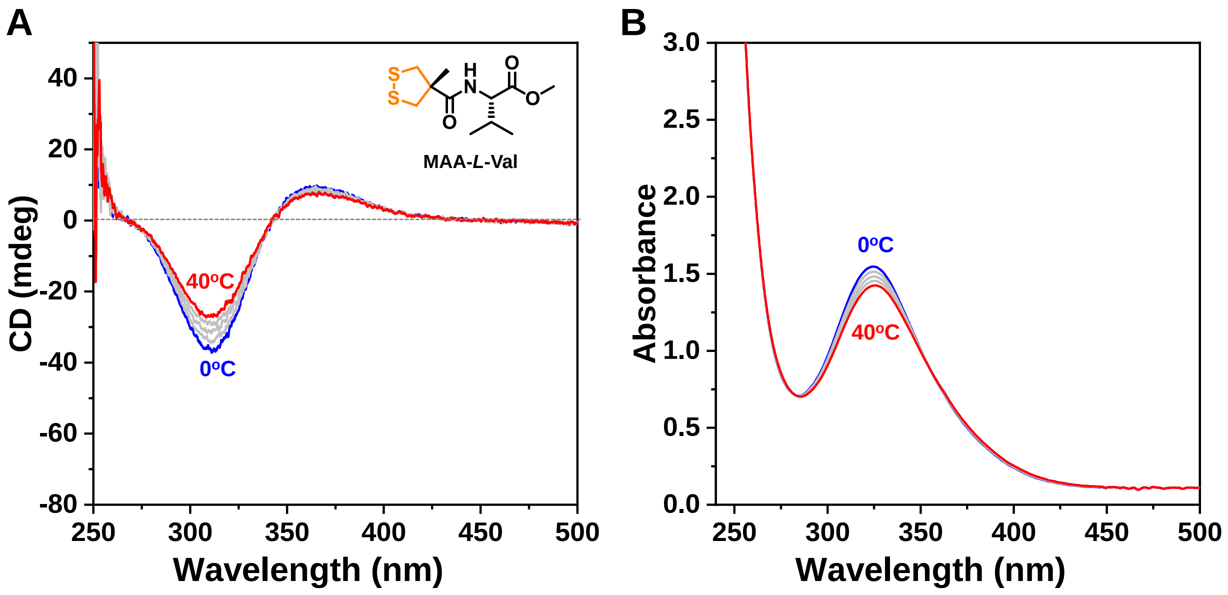

Fig. S25.

Temperature-Varied CD (A) and UV-Vis absorption spectra (B) of MAA-*L*-Val in $CHCl_3$ (**7.5** mM). Optical path = 10 mm; Temperature region was set up from 273K (blue) to 313K (red) with 10K increments.

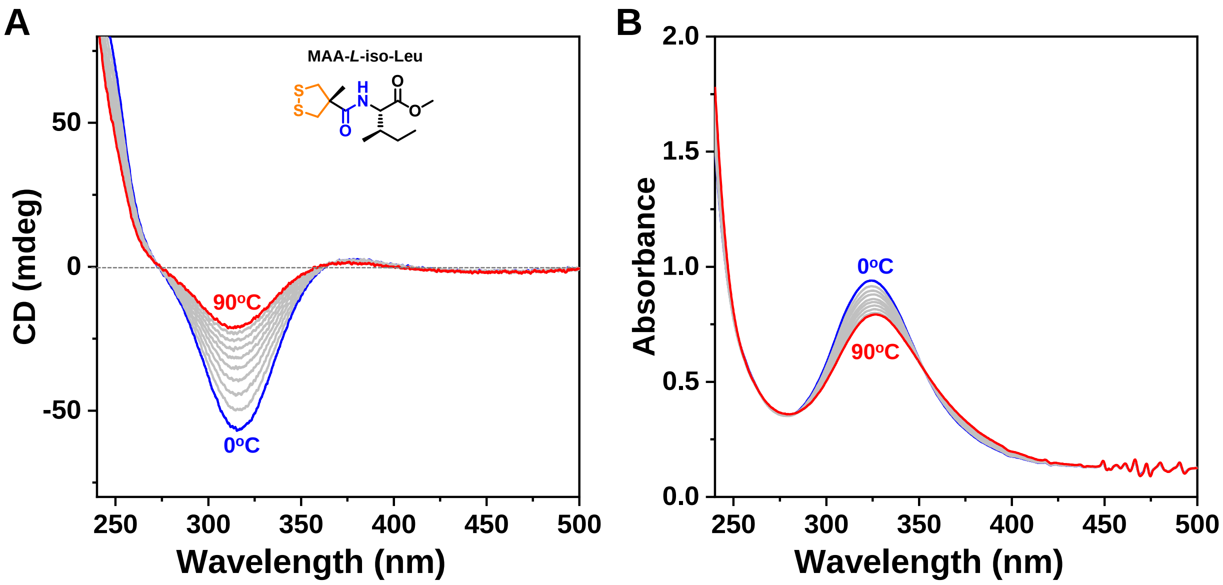

Fig. S26.

Temperature-Varied CD (A) and UV-Vis absorption spectra (B) of MAA-*L*-i-Leu in MCH (4 mM). Optical path = 10 mm; Temperature region was set up from 273K (blue) to 363K (red) with 10K increments.

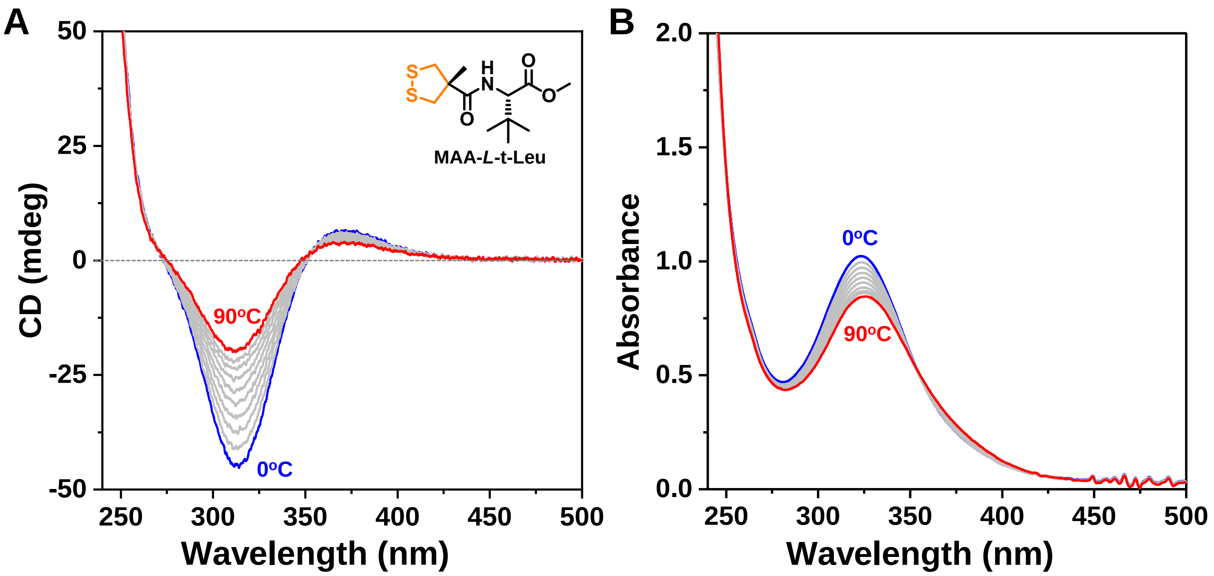

Fig. S27.

Temperature-Varied CD (A) and UV-Vis absorption spectra (B) of MAA-*L*-t-Leu in MCH (4.48 mM). Optical path = 10 mm; Temperature region was set up from 273K (blue) to 363K (red) with 10K increments.

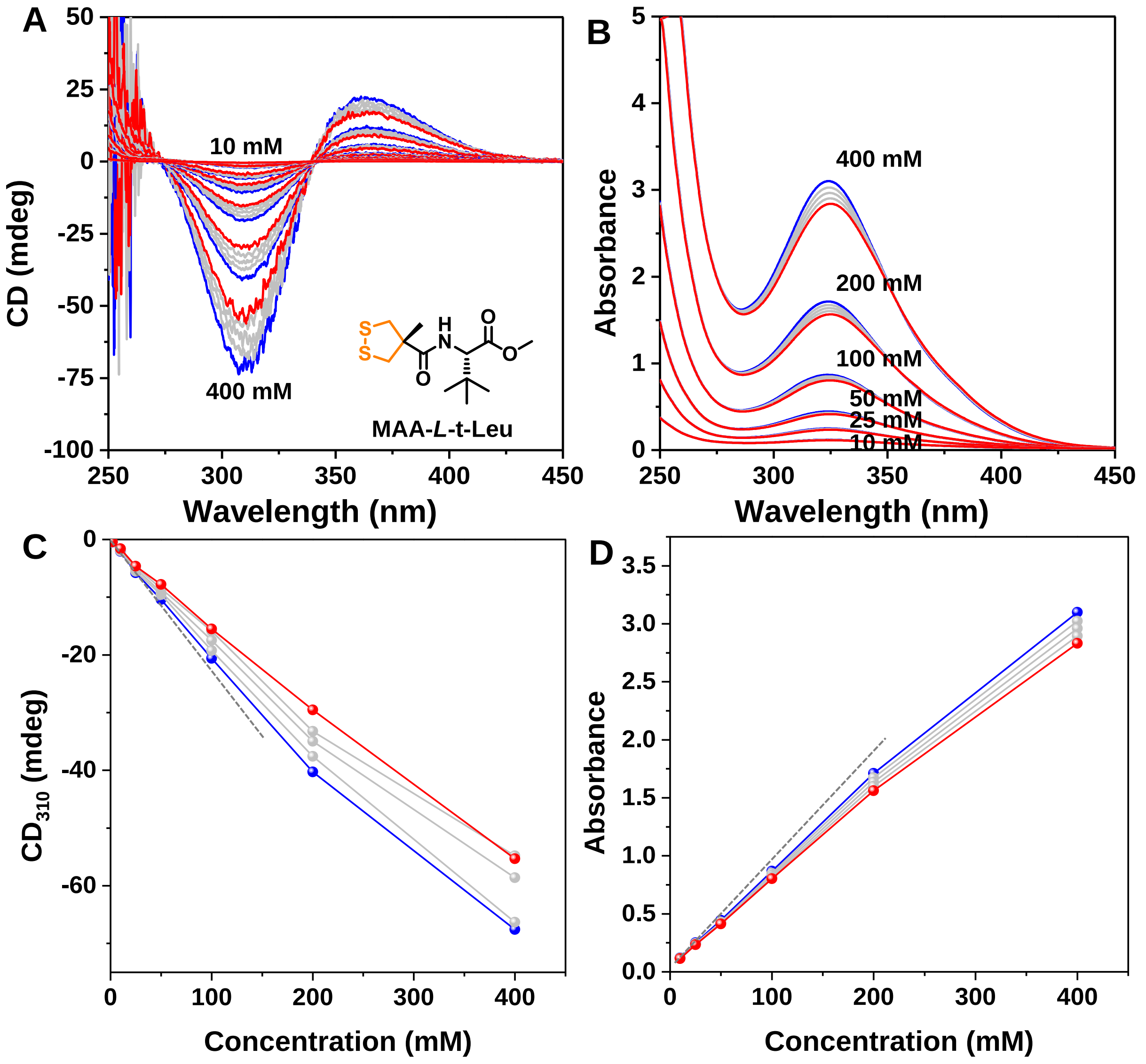

Fig. S28.

Temperature-Varied CD (A, C) and UV-Vis absorption spectra (B, D) of MAA-*L*-t-Leu-OMe in $CHCl_3$ at varied concentrations (10 mM ~ 400 mM). Optical path = 0.5 mm; Temperature region was set up from 273K (blue) to 313K (red) with 10K increments.

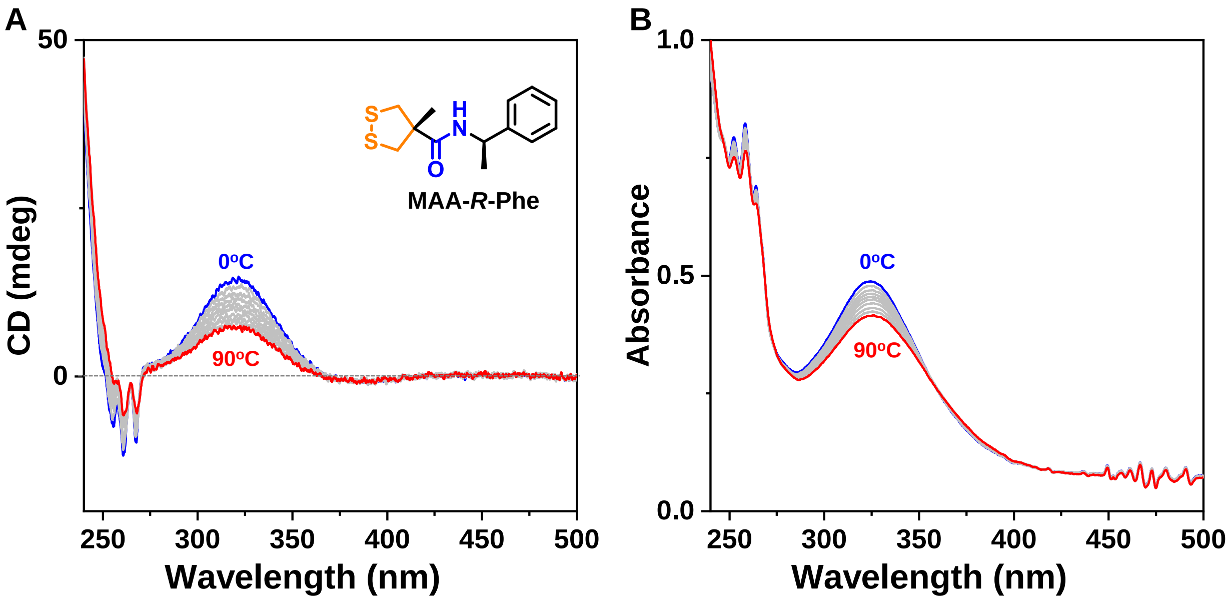

Fig. S29.

Temperature-Varied CD (A) and UV-Vis absorption spectra (B) of MAA-*R*-Phe in MCH (2 mM). Optical path = 10 mm; Temperature region was set up from 273K (blue) to 363K (red) with 10K increments.

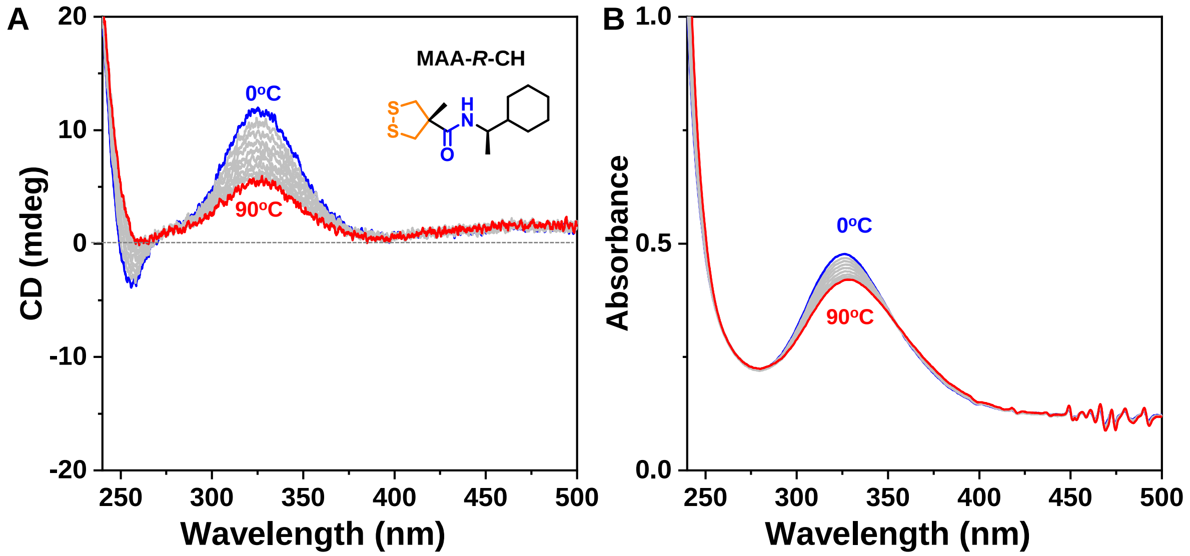

Fig. S30.

Temperature-Varied CD (A) and UV-Vis absorption spectra (B) of MAA-*R*-CH in MCH (2 mM). Optical path = 10 mm; Temperature region was set up from 273K (blue) to 363K (red) with 10K increments.

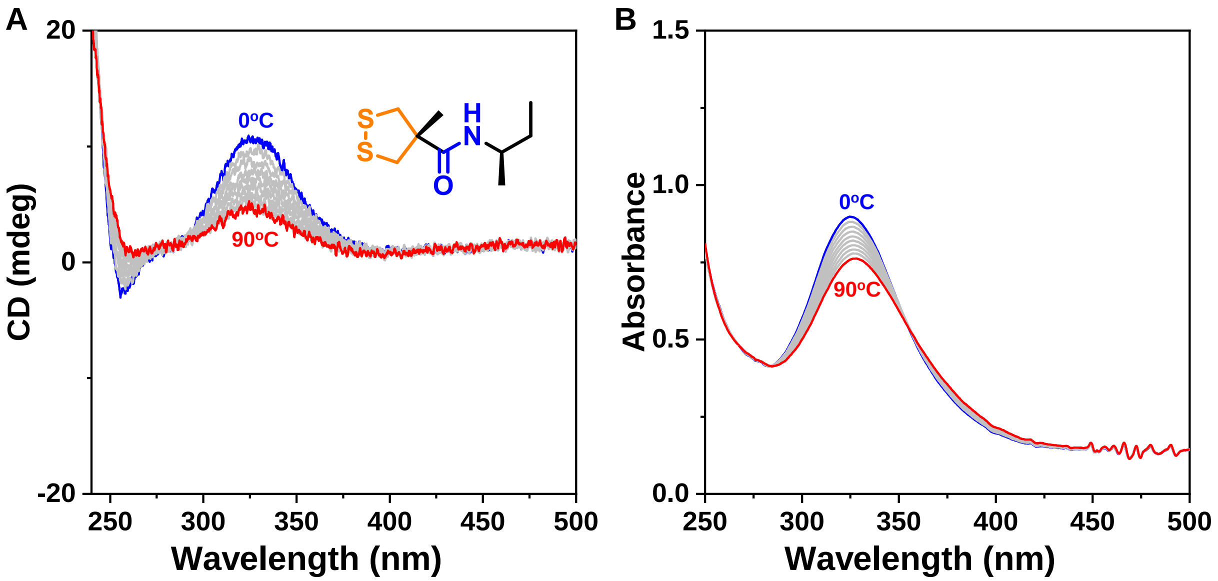

Fig. S31.

Temperature-Varied CD (A) and UV-Vis absorption spectra (B) of MAA-*R*-Butyl in MCH (3.94 mM). Optical path = 10 mm; Temperature region was set up from 273K (blue) to 363K (red) with 10K increments.

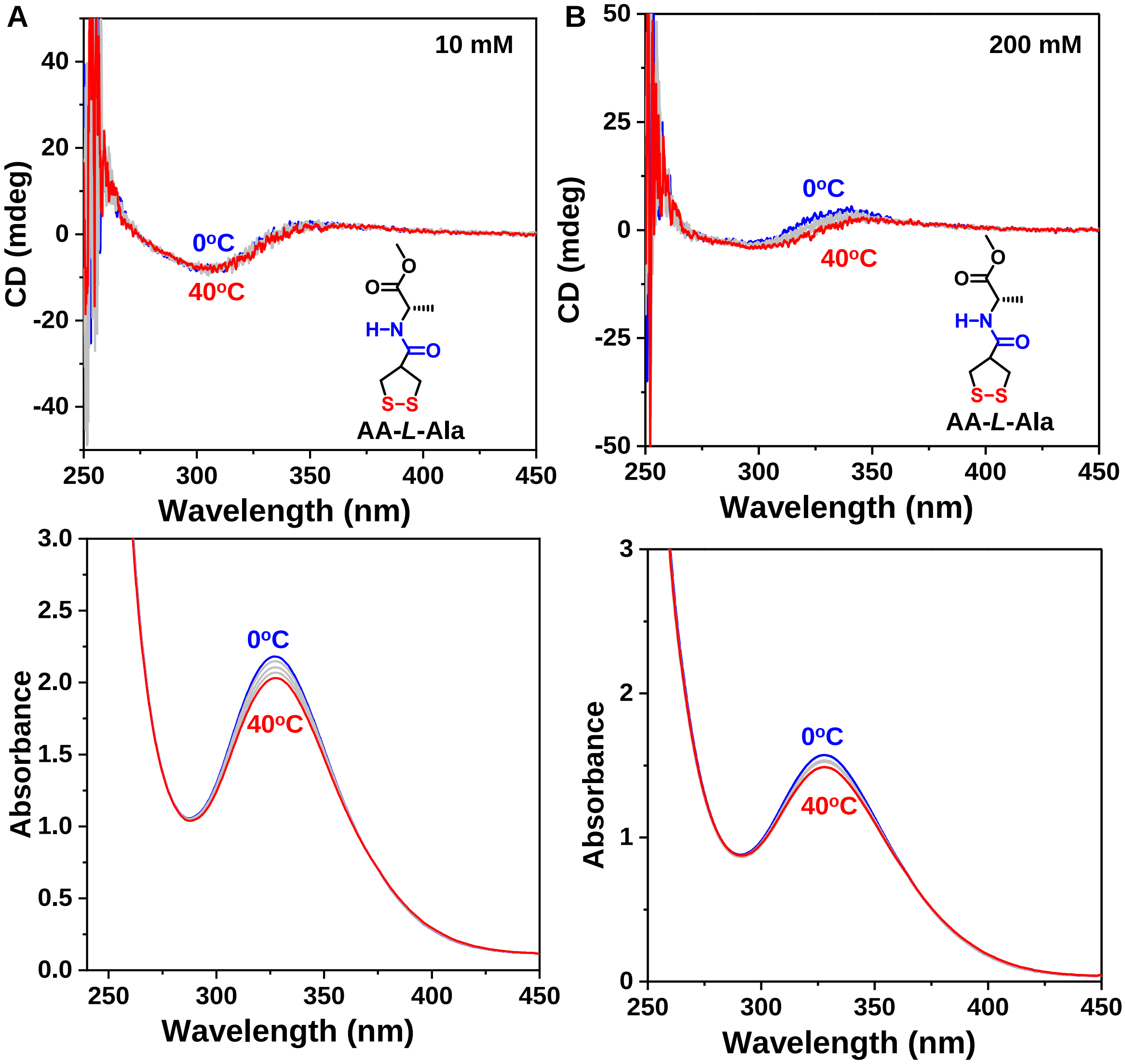

Fig. S32.

Temperature-Varied CD and UV-Vis absorption spectra of AA-*L*-Ala in $CHCl_3$ with different concentration: (A) 10 mM; Optical path = 10 mm; (B) 200 mM; Optical path = 0.5 mm; Temperature region was set up from 273K (blue) to 313K (red) with 10K increments. In diluted solution (A), the CD signal of AA-*L*-Ala exhibited a weak *M* band at around 300 nm, which is attributed to the minor existence of intramolecularly hydrogen-bonded conformers. The remarkable difference of the chirality transfer efficiency between MAA-*L*-Ala and AA-*L*-Ala was due to the less favored formation of S-S···H-N hydrogen bonds caused by the lack of methyl compression effect in AA-*L*-Ala. In concentrated solution (B), the CD signal is almost “silent” in the disulfide region, which is attributed to the more preponderant formation of intermolecular hydrogen bonds in high-concentration solutions.

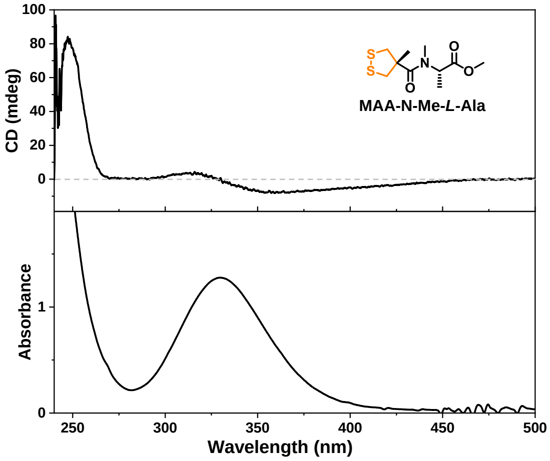

Fig. S33.

CD and UV-Vis spectra of MAA-N-Me-*L*-Ala in MCH (4.2 mM; Optical path = 10 mm; 298K). The CD spectrum of MAA-N-Me-L-Ala in MCH shows much lower CD signal than MAA-*L*-Ala, indicating the poor efficiency of intramolecular chirality transfer in the absence of S-S···H-N hydrogen bond.

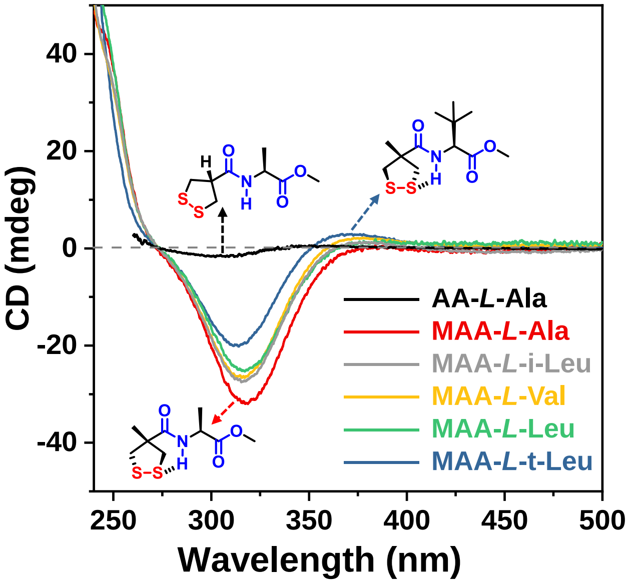

Fig. S34.

Summary of the CD spectra of a series of analogues. The first (~ 370 nm, positive) and the second CD bands (~ 320 nm, negative) visibly shifted with the different bulky residues of amino acids, indicating the structure-encoding stereochemistry and chirality transfer. The remarkable difference between AA-*L*-Ala and MAA-*L*-Ala also presented the methyl effect on the intramolecular chirality transfer process. For fair comparison, all the spectra were normalized as the concentration of 2 mM (collecting temperature: 273K, optical path = 10 mm). Except AA-*L*-Ala sample collected in $CHCl_3$ due to the solubility, all other samples were measured in MCH.

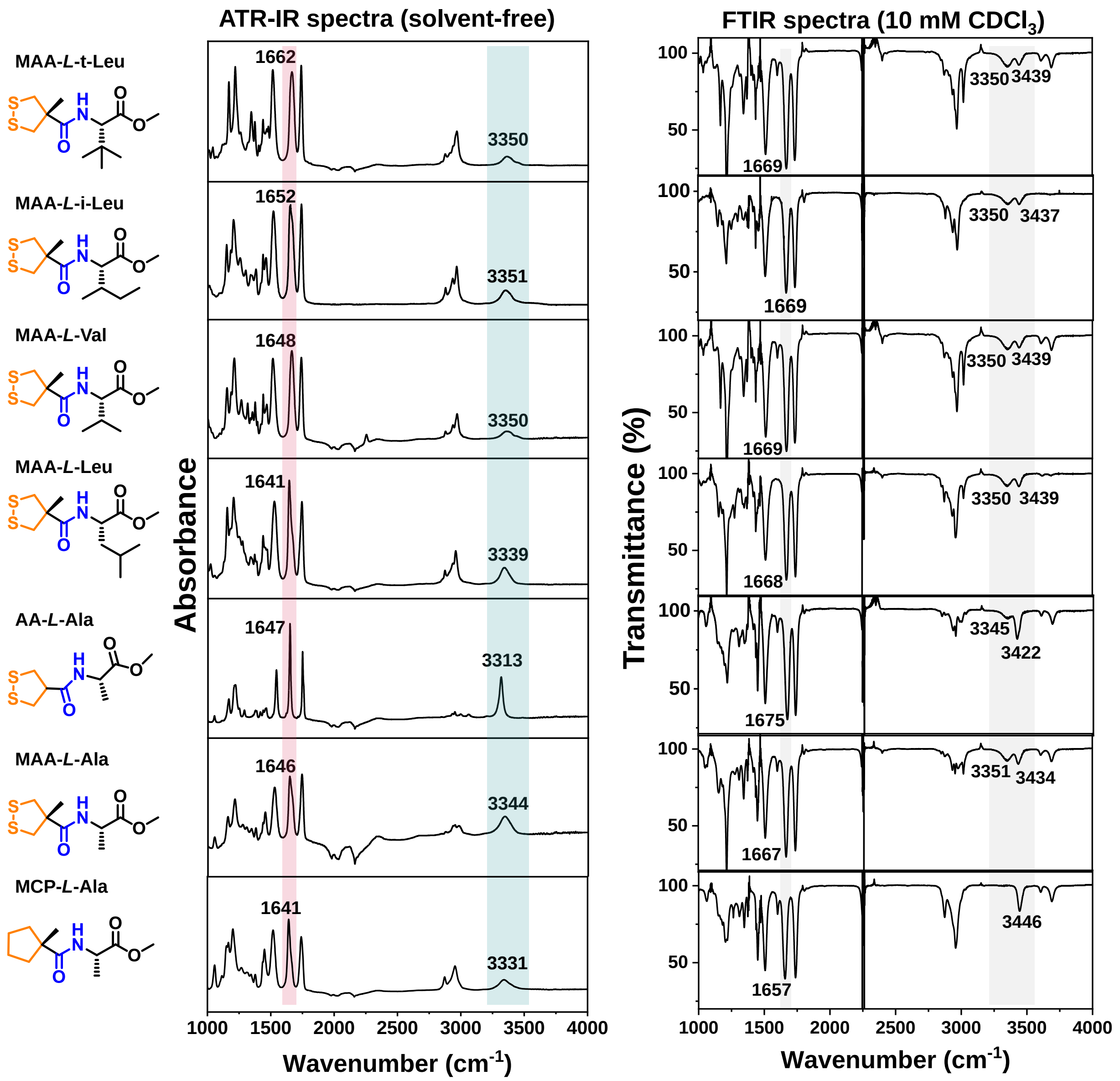

Fig. S35.

IR spectra of the analogues in solvent-free state (ATR) or solution state (IR) in $CDCl_3$ (10 mM).

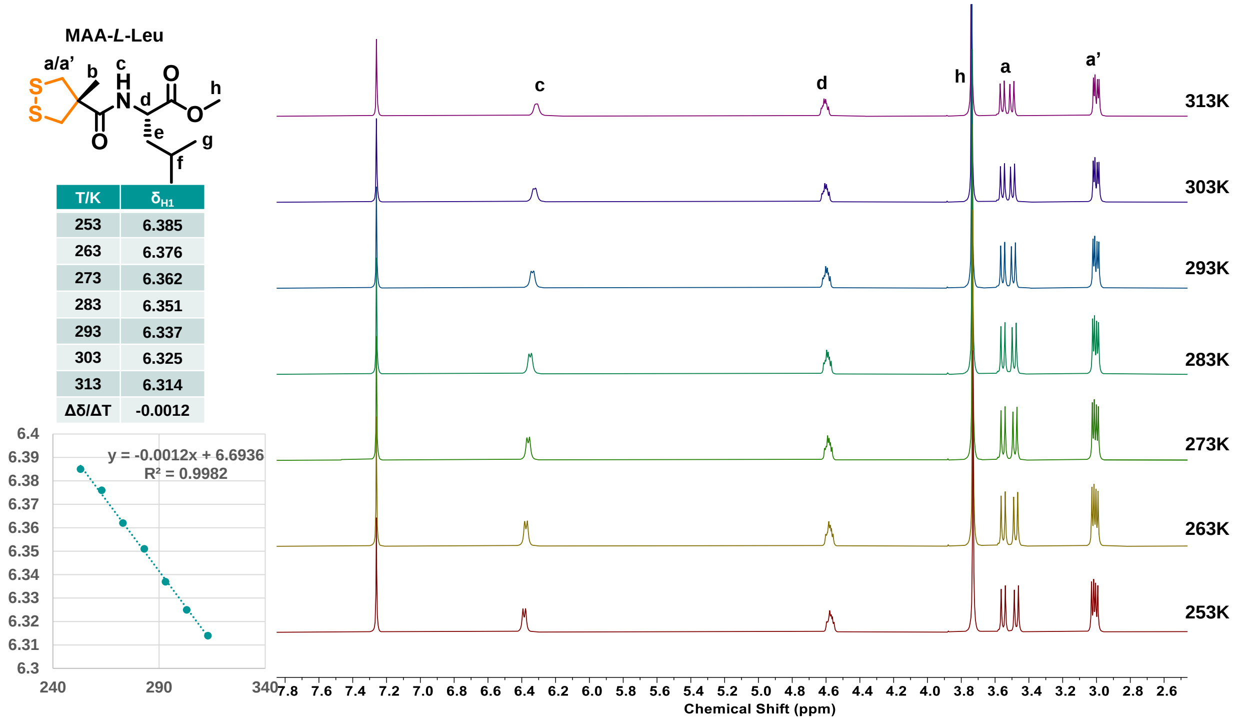

T/K	δ_{H1}
253	6.385
263	6.376
273	6.362
283	6.351
293	6.337
303	6.325
313	6.314
Δδ/ΔT	-0.0012

Fig. S36.

Temperature-Varied ^{1}H NMR spectra of MAA-*L*-Leu in CDCl3 (500 MHz, 10 mM). See detailed structural characterization in Fig. S75-S77.

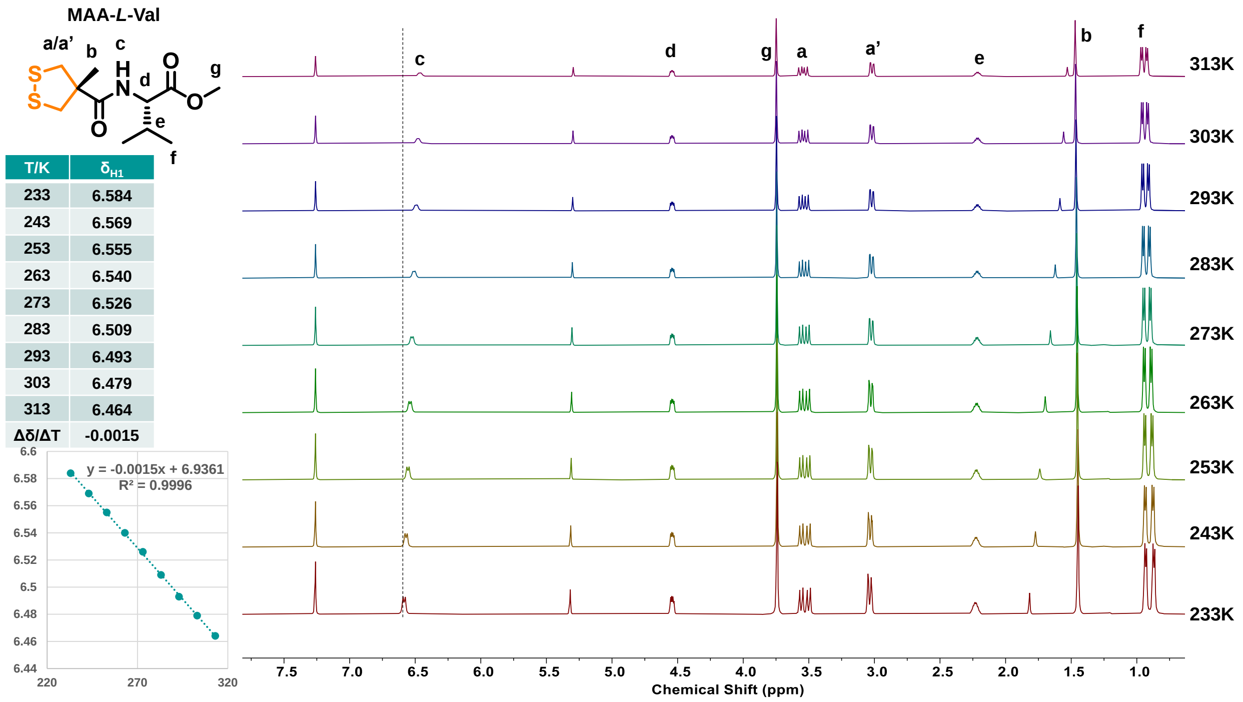

T/K	δ_{H1}
233	6.584
243	6.569
253	6.555
263	6.540
273	6.526
283	6.509
293	6.493
303	6.479
313	6.464
Δδ/ΔT	-0.0015

Fig. S37.

Temperature-varied ^{1}H NMR spectra of MAA-*L*-Val in CDCl3 (500 MHz, 10 mM). See detailed structural characterization in Fig. S78-S80.

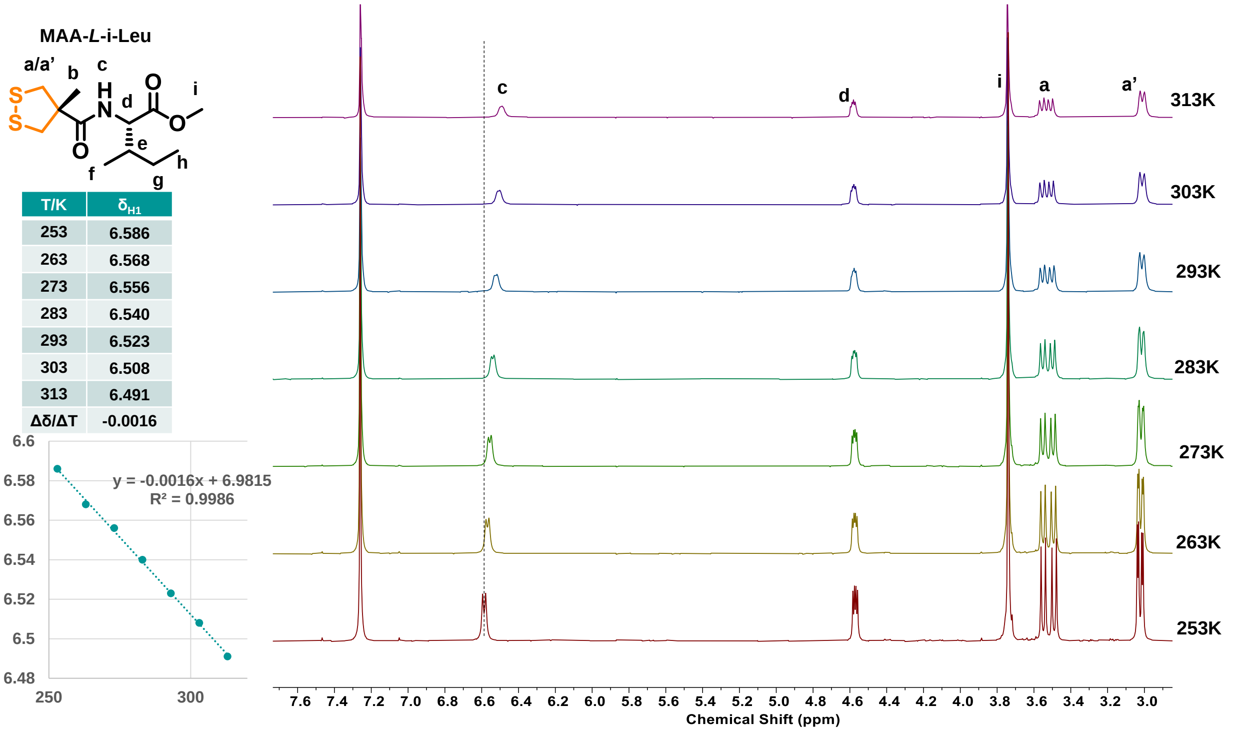

T/K	δ_{H1}
253	6.586
263	6.568
273	6.556
283	6.540
293	6.523
303	6.508
313	6.491
Δδ/ΔT	-0.0016

Fig. S38.

Temperature-varied ^{1}H NMR spectra of MAA-*L*-i-Leu in $CDCl_3$ (500 MHz, 10 mM). See detailed structural characterization in Fig. S81-S83.

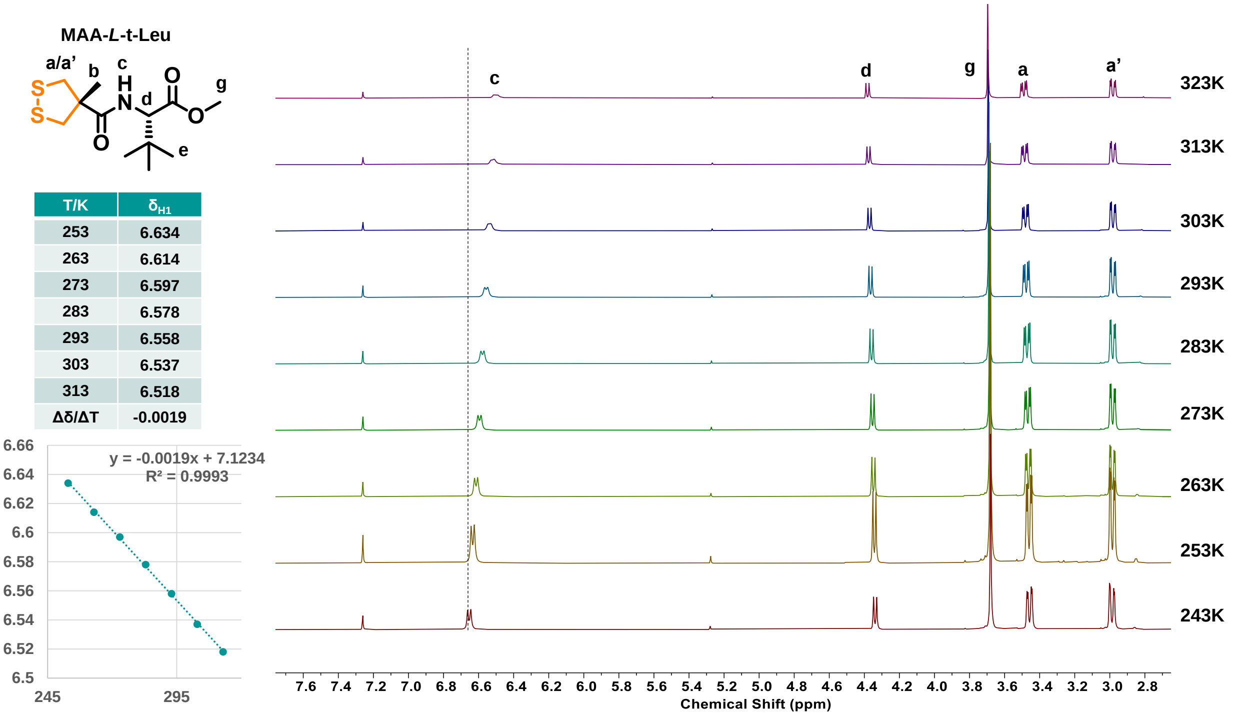

T/K	δ_{H1}
253	6.634
263	6.614
273	6.597
283	6.578
293	6.558
303	6.537
313	6.518
Δδ/ΔT	-0.0019

Fig. S39.

Temperature-varied ^{1}H NMR spectra of MAA-*L*-t-Leu in $CDCl_3$ (500 MHz, 400 mM). See detailed structural characterization in Fig. S84-S86.

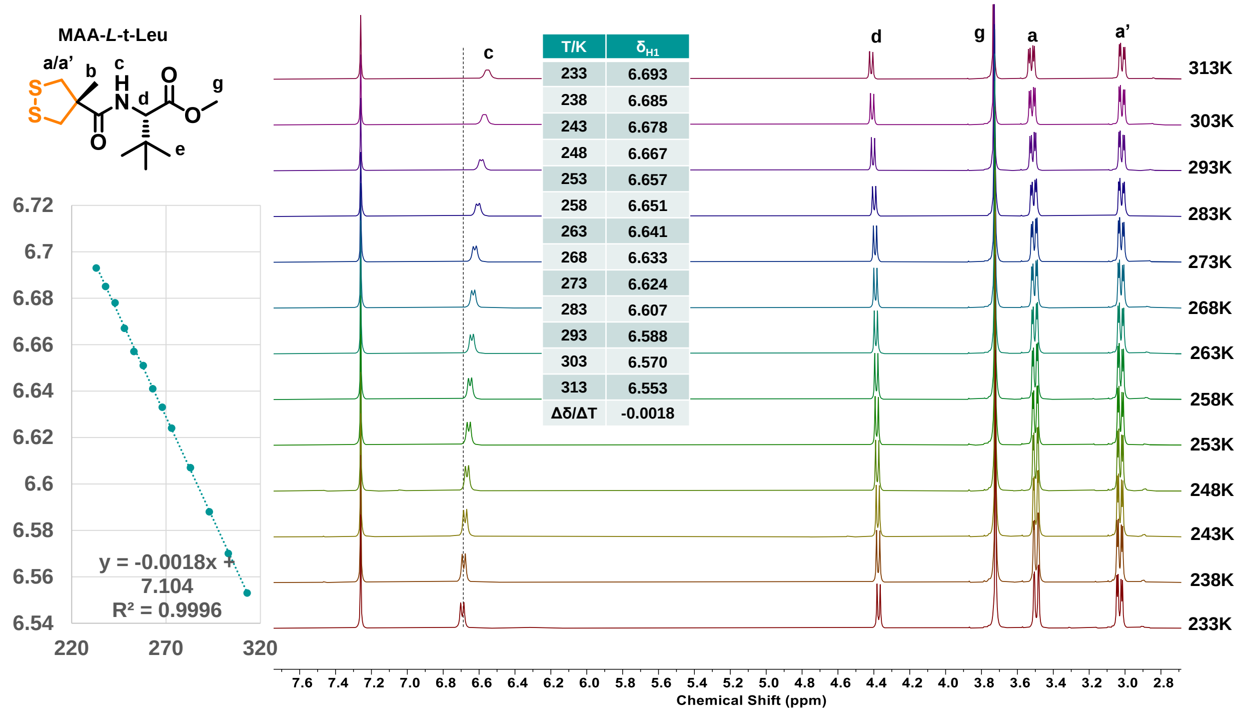

T/K	δ_{H1}
233	6.693
238	6.685
243	6.678
248	6.667
253	6.657
258	6.651
263	6.641
268	6.633
273	6.624
283	6.607
293	6.588
303	6.570
313	6.553
Δδ/ΔT	-0.0018

Fig. S40.

Temperature-varied ^{1}H NMR spectra of MAA-*L*-t-Leu in $CDCl_3$ (500 MHz, 10 mM).

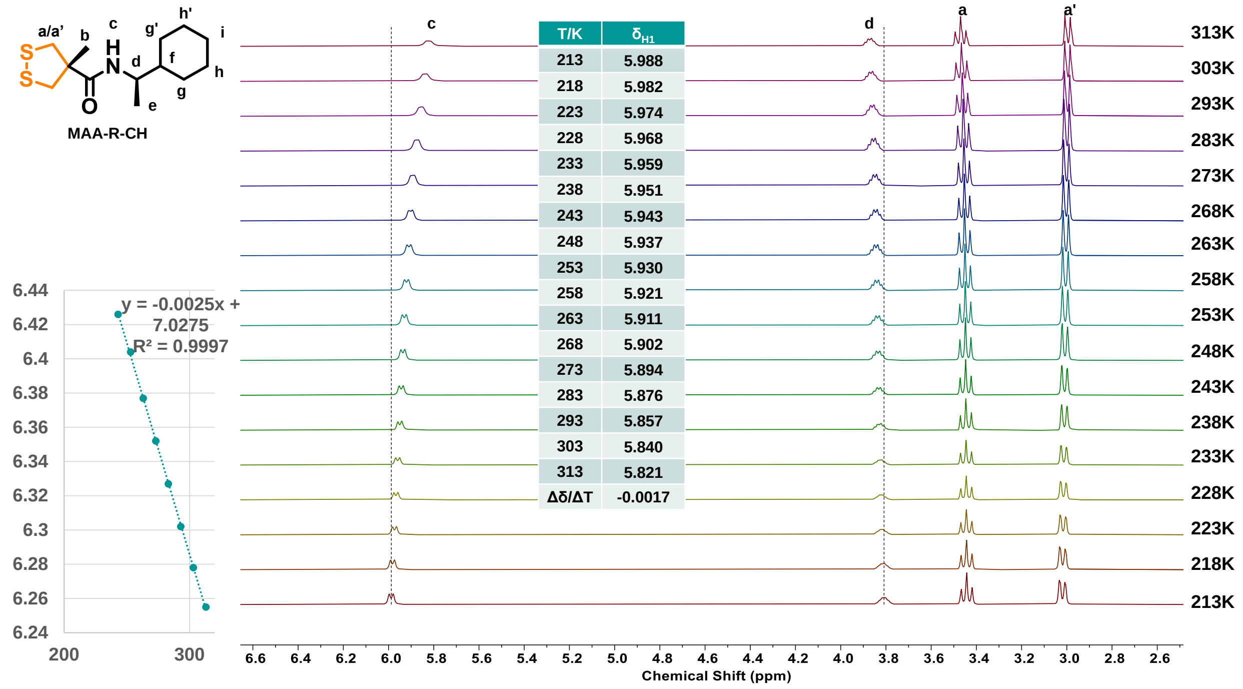

T/K	δ_{H1}
213	5.988
218	5.982
223	5.974
228	5.968
233	5.959
238	5.951
243	5.943
248	5.937
253	5.930
258	5.921
263	5.911
268	5.902
273	5.894
283	5.876
293	5.857
303	5.840
313	5.821
Δδ/ΔT	-0.0017

Fig. S41.

Temperature-Varied ^{1}H NMR spectra of MAA-*R*-CH in $CDCl_3$ (500 MHz, 10 mM). See detailed structural characterization in Fig. S90-S92.

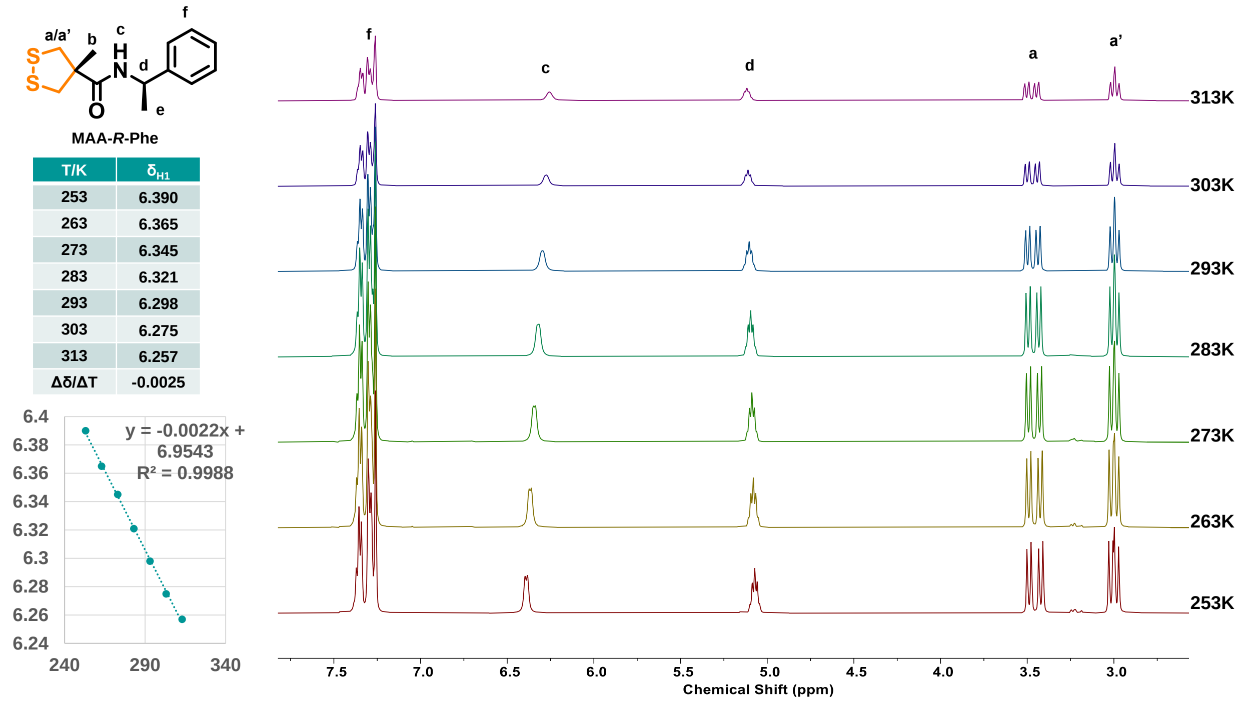

T/K	δ_{H1}
253	6.390
263	6.365
273	6.345
283	6.321
293	6.298
303	6.275
313	6.257
Δδ/ΔT	-0.0025

Fig. S42.

Temperature-varied ^{1}H NMR spectra of MAA-*R*-Phe in $CDCl_3$ (500 MHz, 10 mM). See detailed structural characterization in Fig. S87-S89.

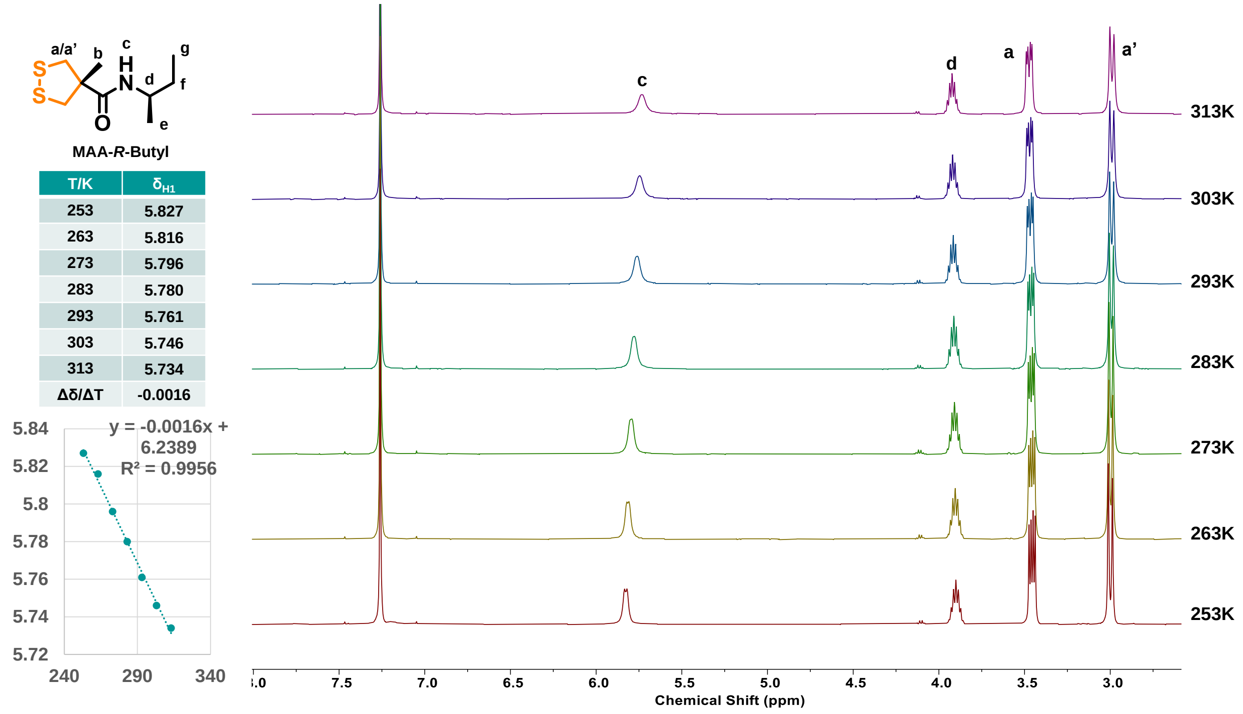

T/K	δ_{H1}
253	5.827
263	5.816
273	5.796
283	5.780
293	5.761
303	5.746
313	5.734
Δδ/ΔT	-0.0016

Fig. S43.

Temperature-varied ^{1}H NMR spectra of MAA-*R*-Butyl in $CDCl_3$ (500 MHz, 10 mM). See detailed structural characterization in Fig. S93-S95.

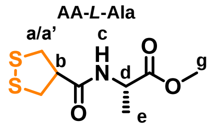

T/K	δ_{H1}
253	6.374
263	6.355
273	6.326
283	6.307
293	6.284
303	6.267
313	6.236
Δδ/ΔT	-0.0023

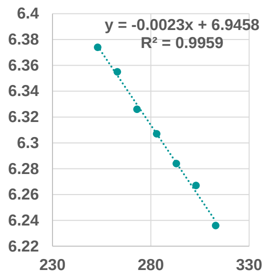

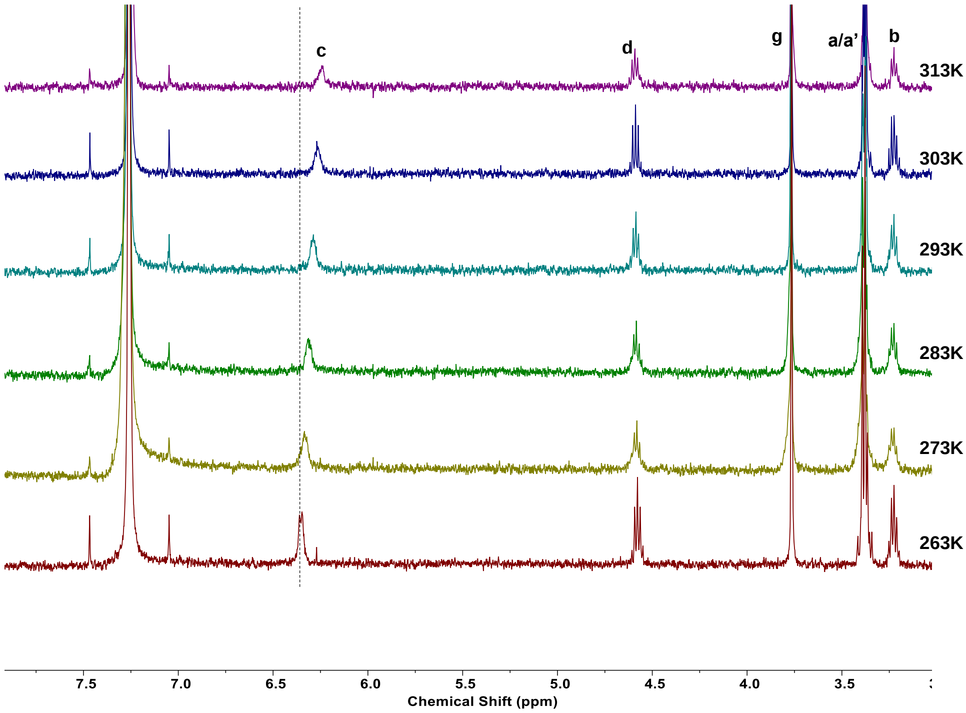

Fig. S44.

Temperature-varied ^{1}H NMR spectra of AA-*L*-Ala in $CDCl_3$ (500 MHz, 2 mM). See detailed structural characterization in Fig. S96-S98.

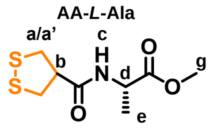

T/K	δ_{H1}
253	6.39
263	6.366
273	6.341
283	6.32
293	6.295
303	6.273
313	6.255
Δδ/ΔT	-0.0023

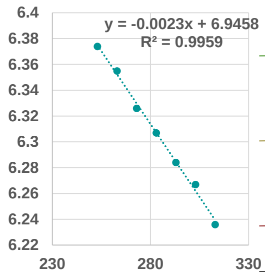

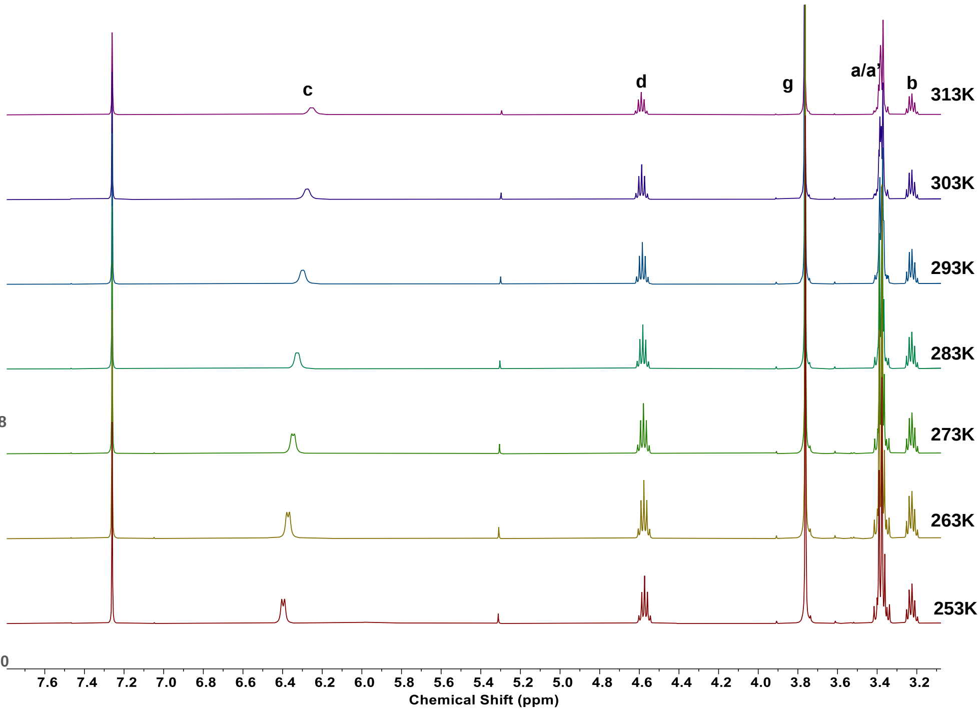

Fig. S45.

Temperature-varied ^{1}H NMR spectra of AA-*L*-Ala in $CDCl_3$ (500 MHz, 20 mM).

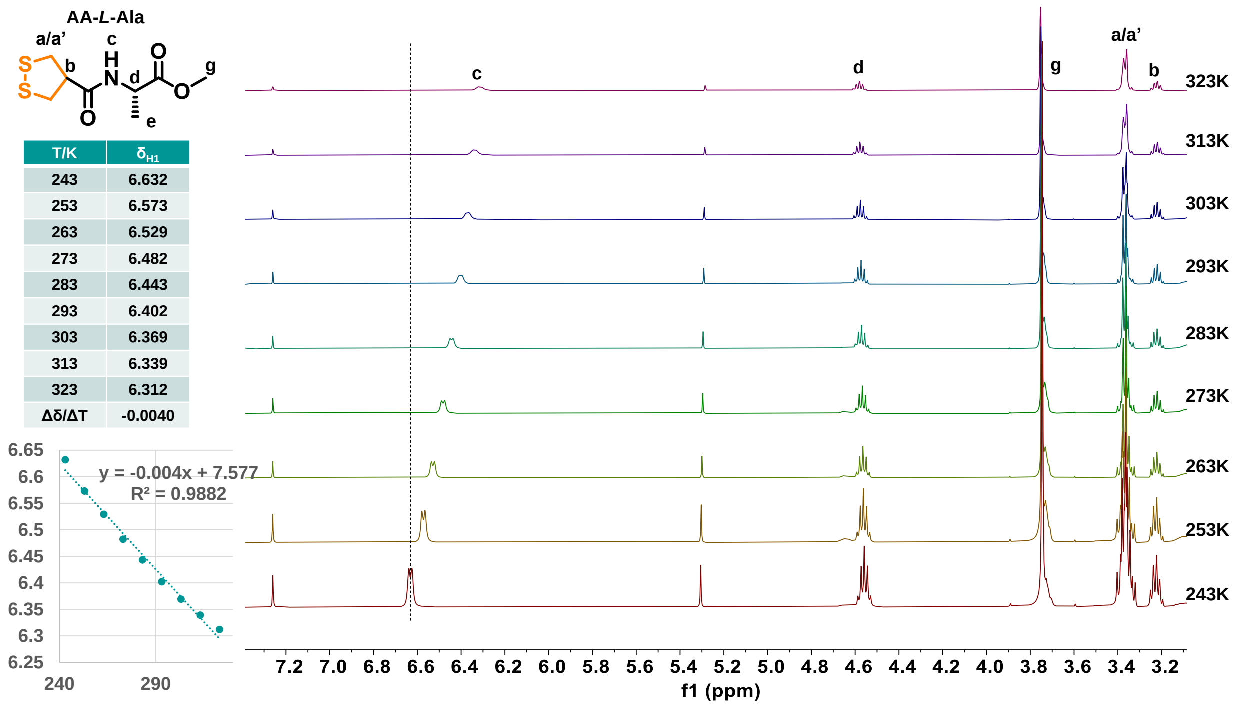

T/K	δ_{H1}
243	6.632
253	6.573
263	6.529
273	6.482
283	6.443
293	6.402
303	6.369
313	6.339
323	6.312
Δδ/ΔT	-0.0040

Fig. S46.

Temperature-varied ^{1}H NMR spectra of AA-*L*-Ala in $CDCl_3$ (500 MHz, 200 mM).

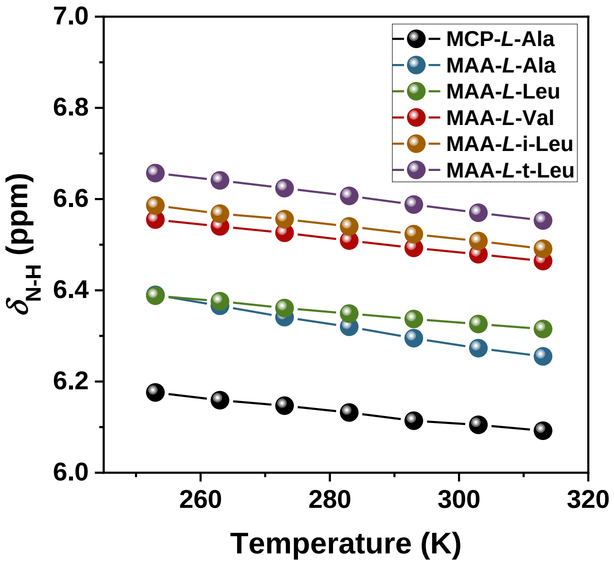

Fig. S47.

Temperature-varied chemical shift ($\delta_{N\text{-}H}$) of the compounds with different substitutes. The data were collected in $CDCl_3$ (10 mM) from 253 K to 313 K.

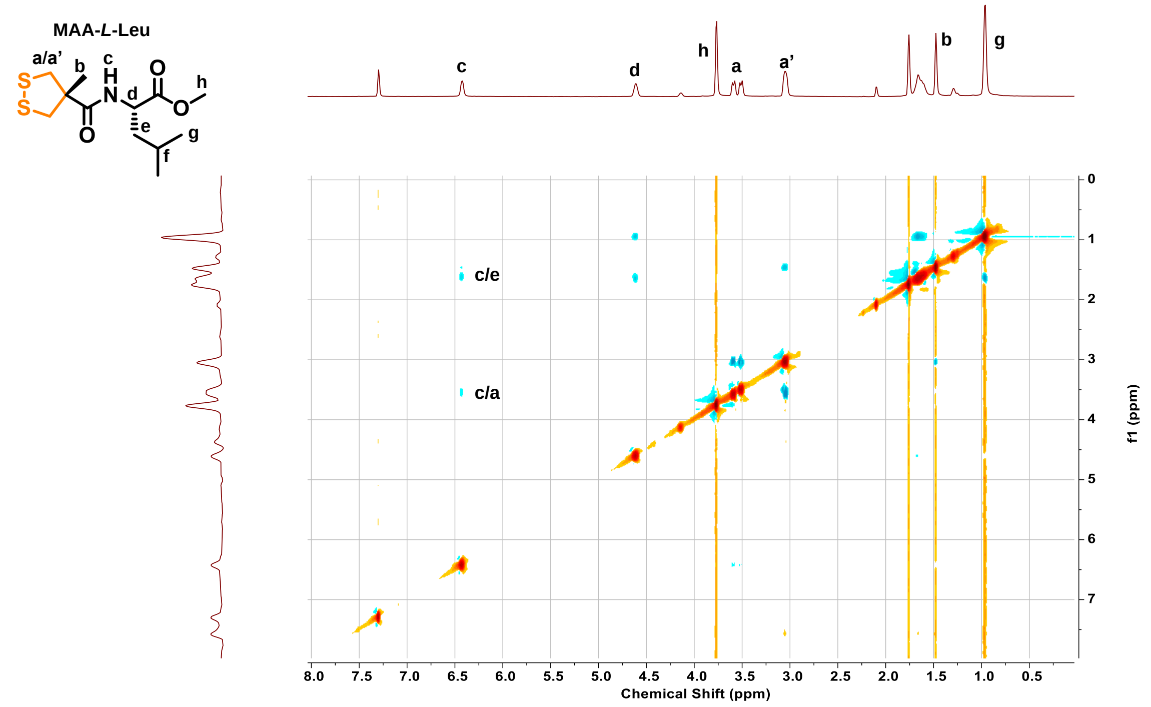

Fig. S48.
2D NOESY analysis of MAA-*L*-Leu (500 MHz, 253K, 10 mM).

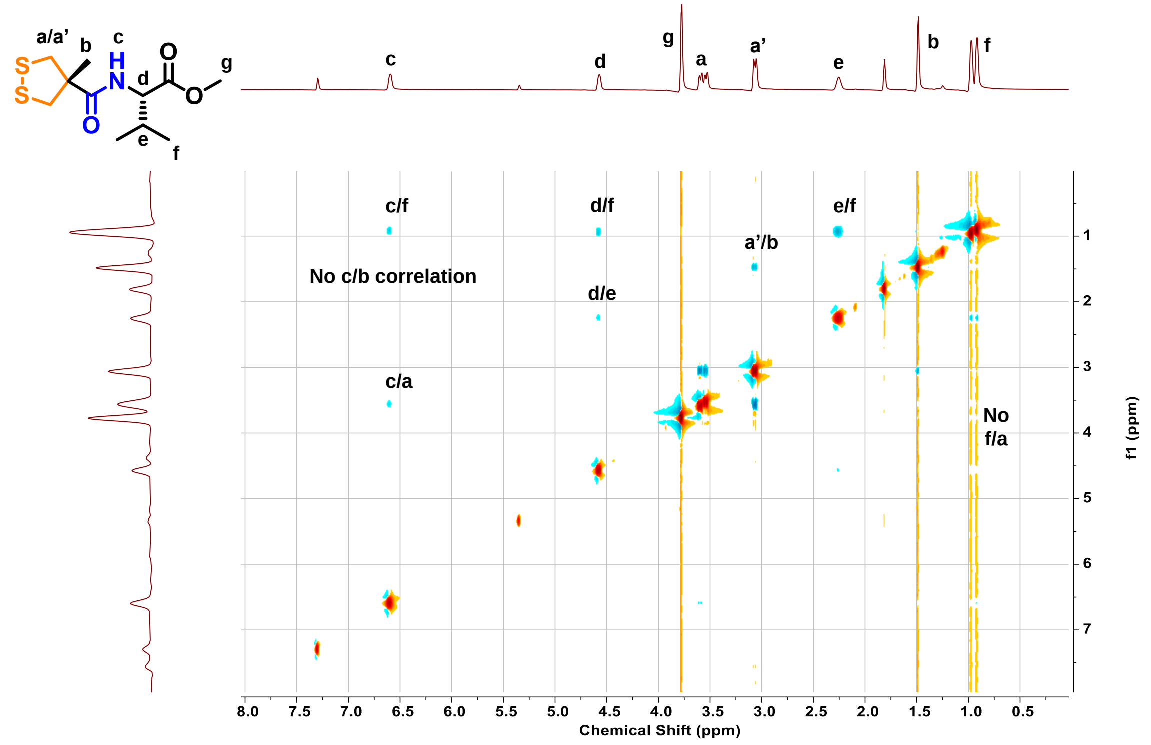

Fig. S49.
2D NOESY analysis of MAA-*L*-Val (500 MHz, 253K, 10 mM).

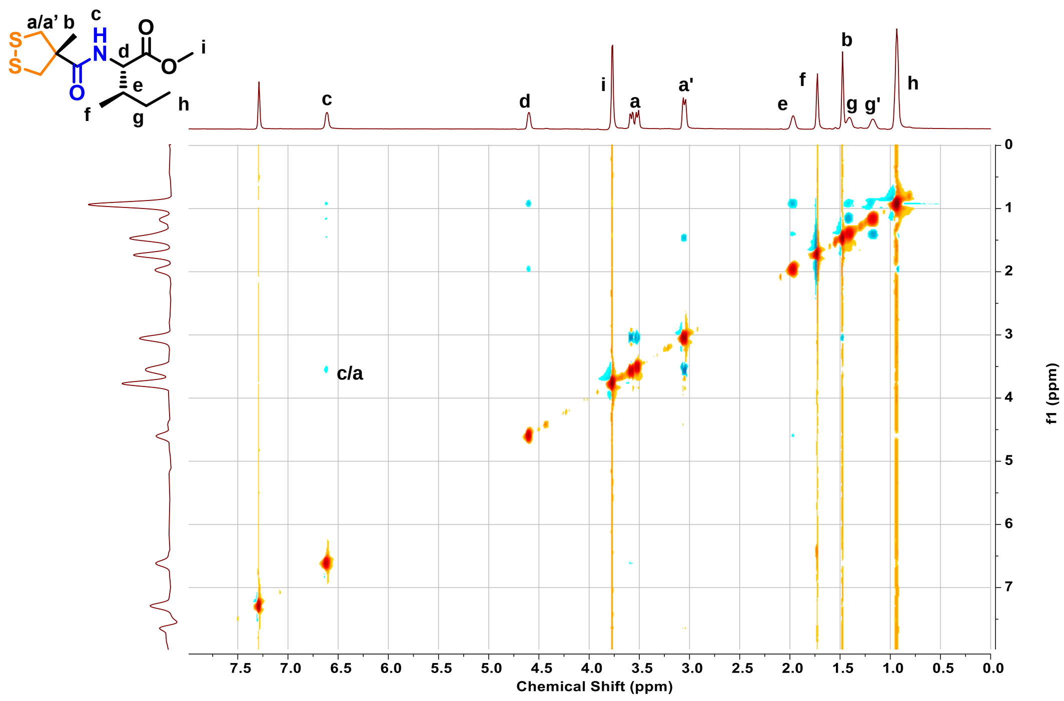

Fig. S50.
2D NOESY analysis of MAA-*L*-i-Leu (500 MHz, 253K, 10 mM).

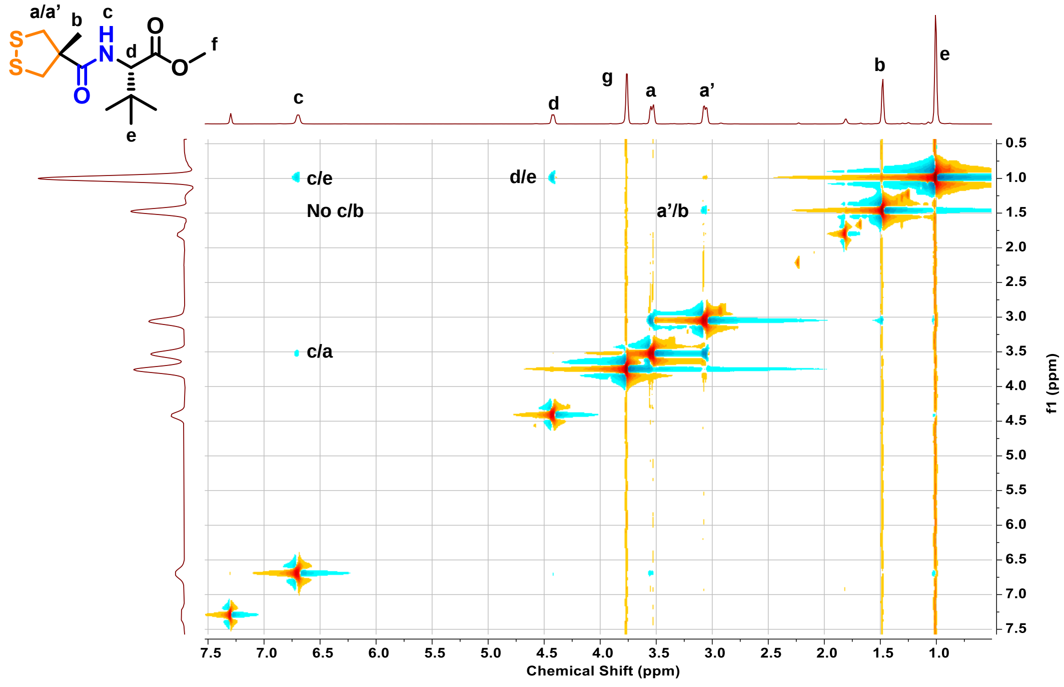

Fig. S51.
2D NOESY analysis of MAA-*L*-t-Leu (500 MHz, 253K, 10 mM).

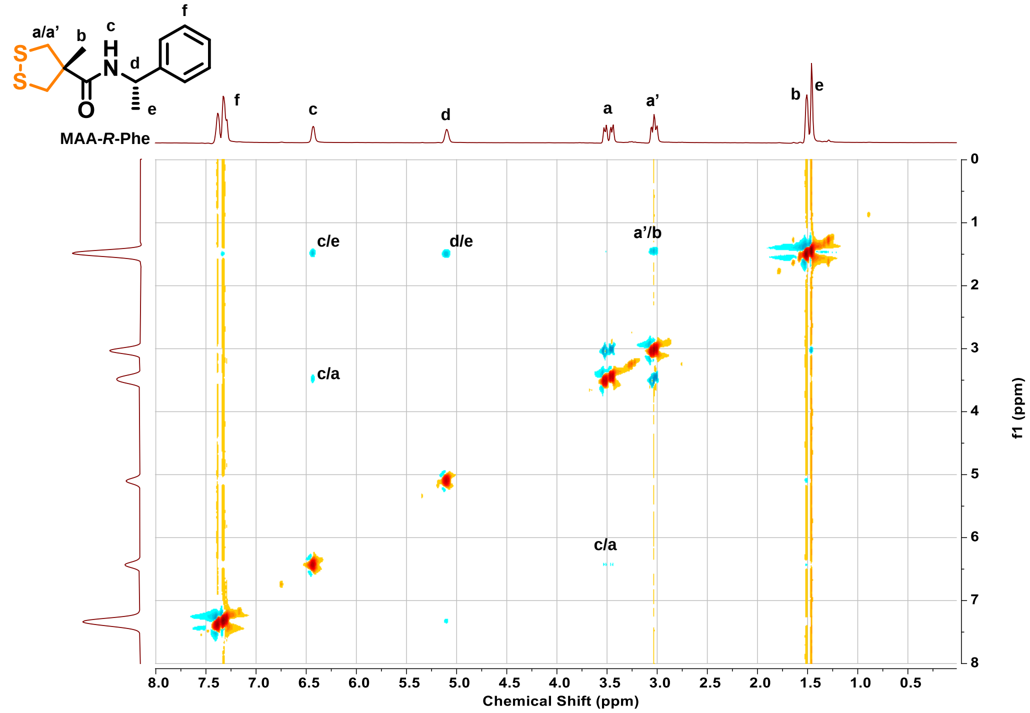

Fig. S52.
2D NOESY analysis of MAA-*R*-Phe (500 MHz, 253K, 10 mM).

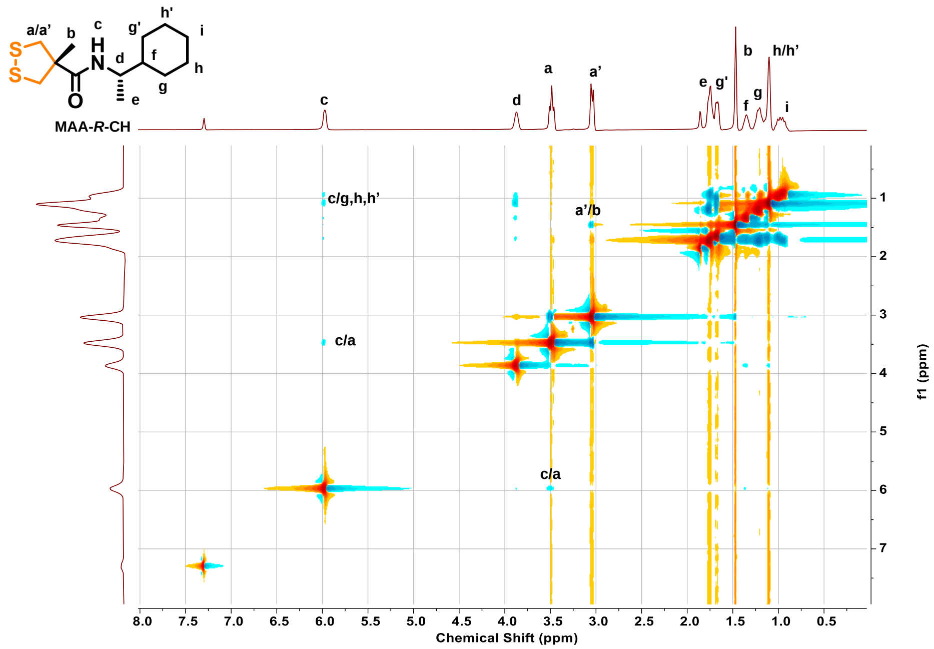

Fig. S53.
2D NOESY analysis of MAA-*R*-CH (500 MHz, 253K, 10 mM).

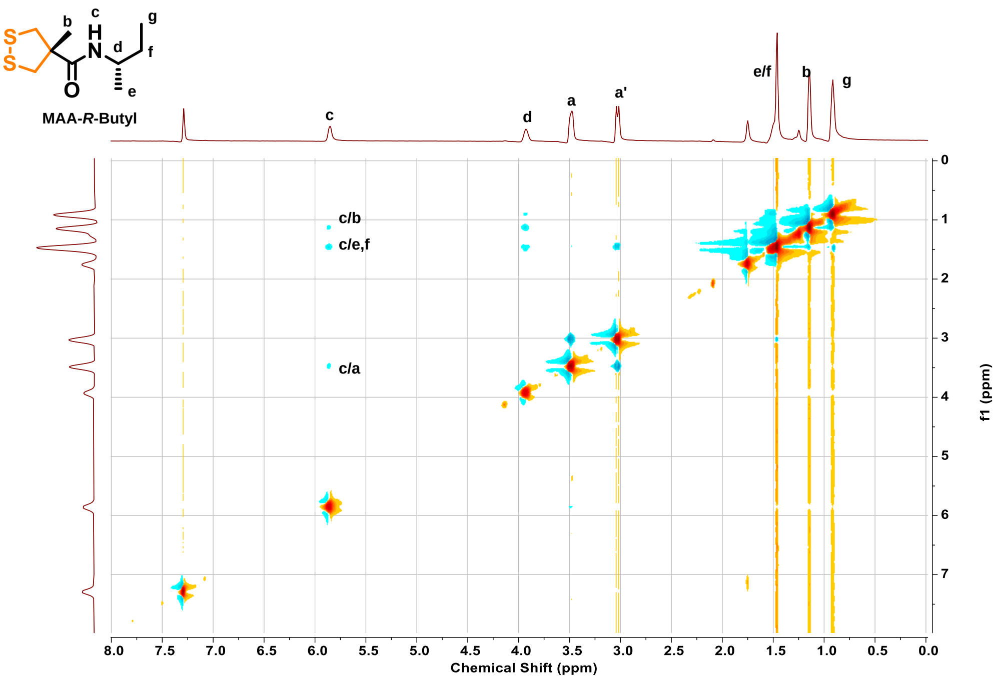

Fig. S54.

2D NOESY analysis of MAA-*R*-Butyl (500 MHz, 253K, 10 mM).

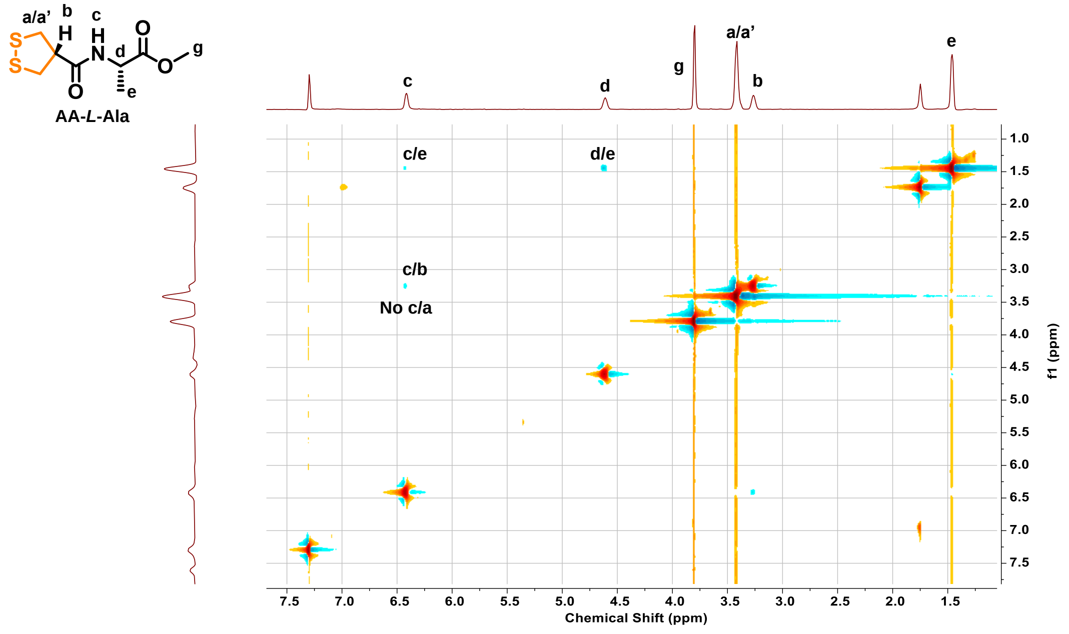

Fig. S55.

2D NOESY spectrum of AA-*L*-Ala in $CDCl_3$ (500 MHz, 253K, 10 mM).

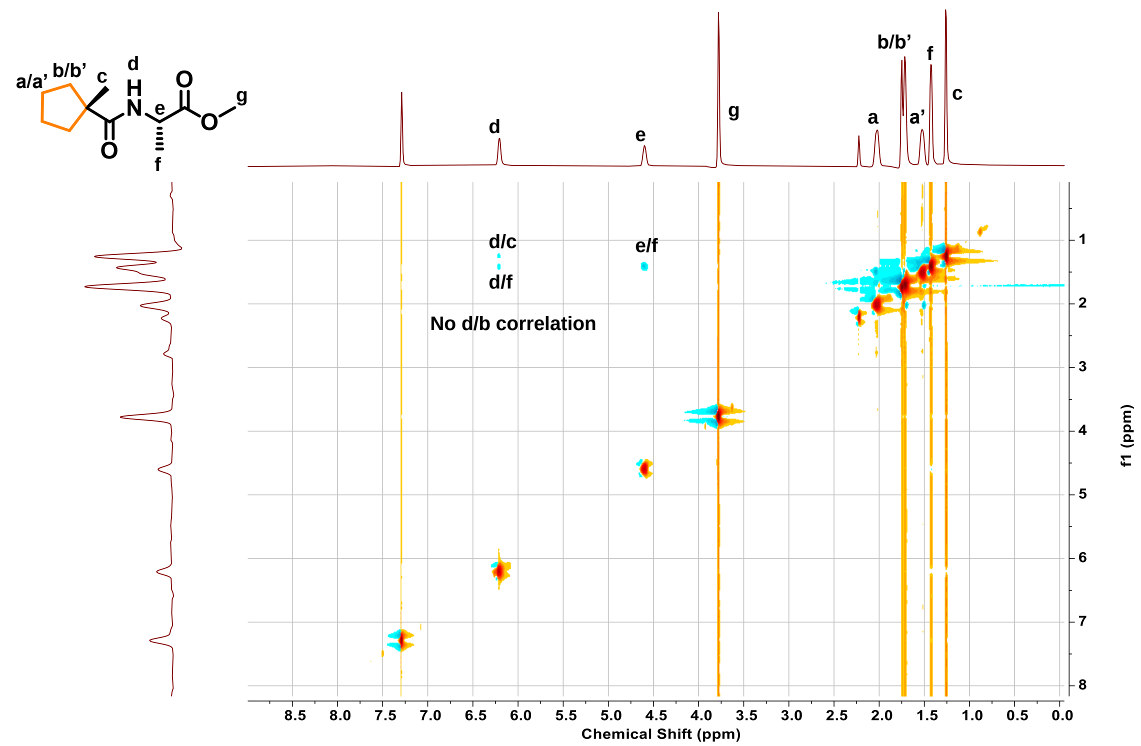

Fig. S56.
2D NOESY spectrum of MCP-*L*-Ala in $CDCl_3$ (500 MHz, 253K, 10 mM).

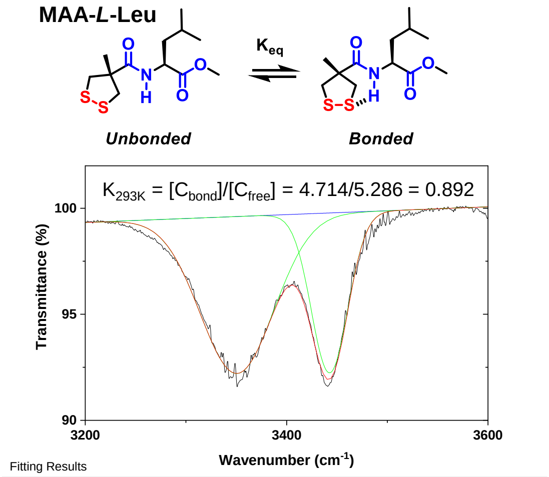

Fitting Results

Peak Index	Peak Type	Area Intg	FWHM	Max Height	Center Grvty	Area IntgP
1	Gaussian	-341.57836	42.58092	-7.53604	3442.69359	-33.44297
2	Gaussian	-679.79734	86.30051	-7.40004	3350.65911	-66.55703

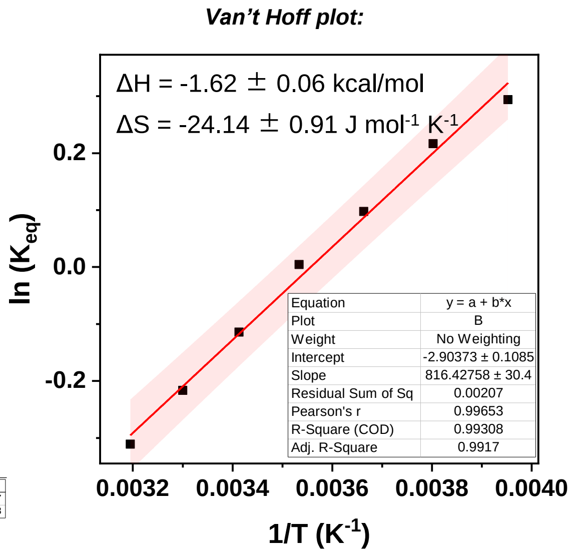

Fig. S57.
IR spectra of the amide vibration band (ν_{NH}) of MAA-*L*-Leu in 10 mM $CDCl_3$ solutions at 293K and the van't Hoff fitting plot according to VT-NMR spectra. Red band indicates 95% confidence intervals.

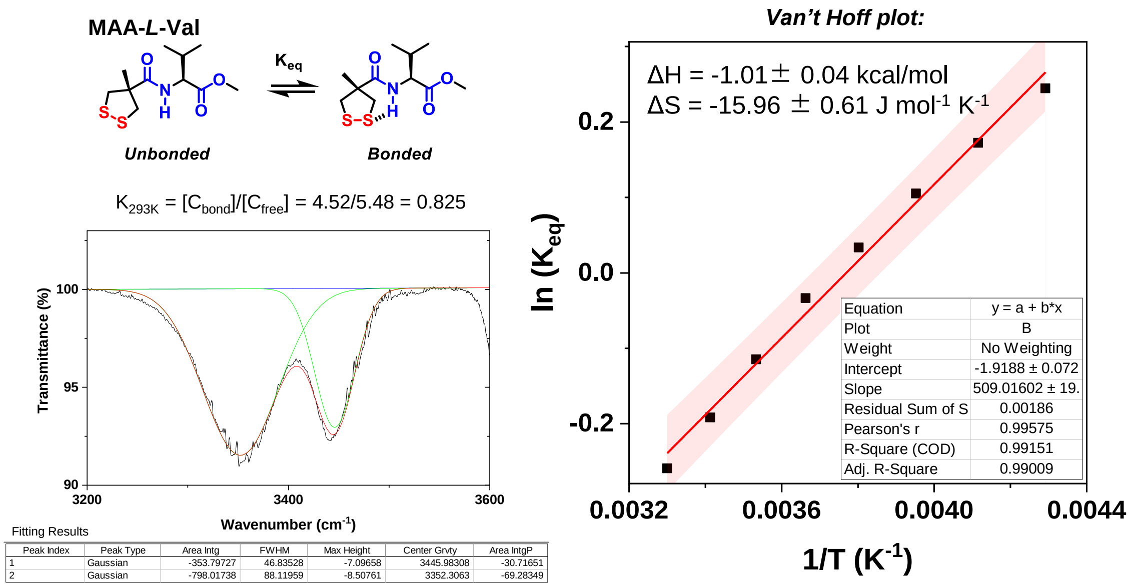

Fitting Results

Peak Index	Peak Type	Area Intg	FWHM	Max Height	Center Grvty	Area IntgP
1	Gaussian	-353.79727	46.83528	-7.09658	3445.98308	-30.71651
2	Gaussian	-798.01738	88.11959	-8.50761	3352.3063	-69.28349

Fig. S58.

IR spectra of the amide vibration band (ν_{NH}) of MAA-*L*-Val in 10 mM $CDCl_3$ solutions at 293K and the van't Hoff fitting plot according to VT-NMR spectra. Red band indicates 95% confidence intervals.

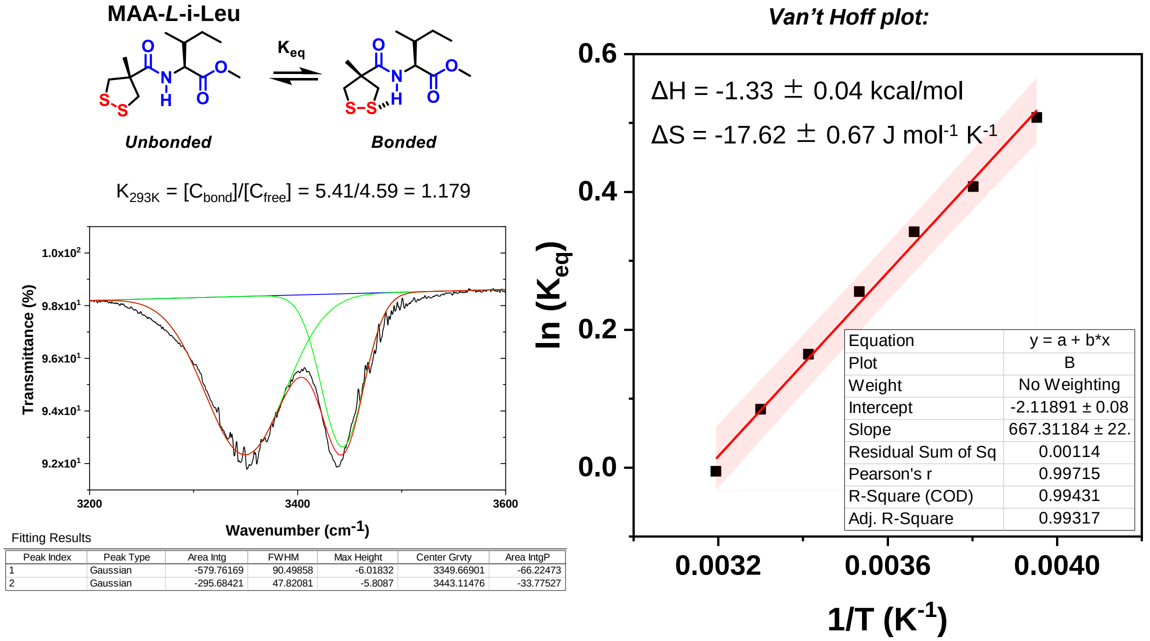

Fitting Results

Peak Index	Peak Type	Area Intg	FWHM	Max Height	Center Grvty	Area IntgP
1	Gaussian	-579.76169	90.49858	-6.01832	3349.66901	-66.22473
2	Gaussian	-295.68421	47.82081	-5.8087	3443.11476	-33.77527

Fig. S59.

IR spectra of the amide vibration band (ν_{NH}) of MAA-*L*-i-Leu in 10 mM $CDCl_3$ solutions at 293K and the van't Hoff fitting plot according to VT-NMR spectra. Red band indicates 95% confidence intervals.

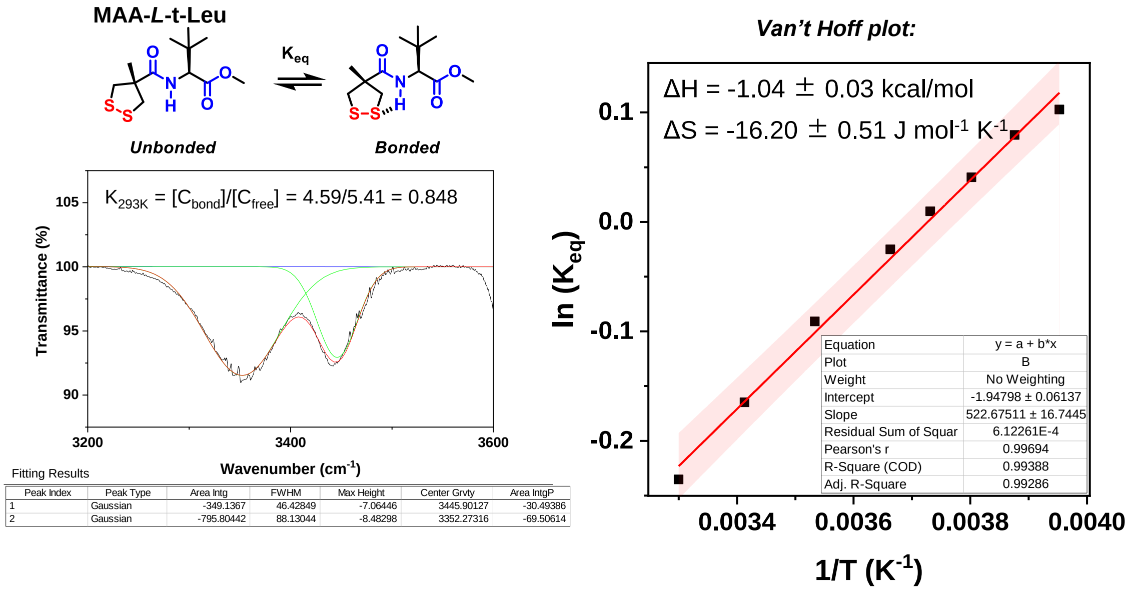

Peak Index	Peak Type	Area Intg	FWHM	Max Height	Center Grvty	Area IntgP
1	Gaussian	-349.1367	46.42849	-7.06446	3445.90127	-30.49386
2	Gaussian	-795.80442	88.13044	-8.48298	3352.27316	-69.50614

Equation	y = a + b*x
Plot	B
Weight	No Weighting
Intercept	-1.94798 ± 0.06137
Slope	522.67511 ± 16.7445
Residual Sum of Squar	6.12261E-4
Pearson's r	0.99694
R-Square (COD)	0.99388
Adj. R-Square	0.99286

Fig. S60.

IR spectra of the amide vibration band (ν_{NH}) of MAA-*L*-t-Leu in 10 mM $CDCl_3$ solutions at 293K and the van't Hoff fitting plot according to VT-NMR spectra. Red band indicates 95% confidence intervals.

	View A	View B	Population Percentage	S-S chirality/φ	S-S···H-N H-bond
1	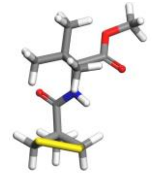	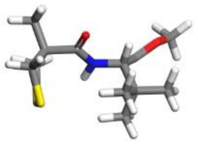	17%	*P* chirality<br>$\varphi_{C\text{-}S\text{-}S\text{-}C}$ = 13°	*l* = 2.61 Å<br>$\theta_{N\text{-}H\text{-}S}$ = 129°
2	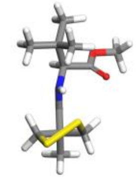	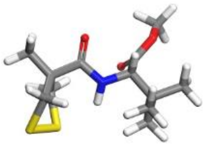	17%	*M* chirality<br>$\varphi_{C\text{-}S\text{-}S\text{-}C}$ = 44°	*l* = 2.53 Å<br>$\theta_{N\text{-}H\text{-}S}$ = 139°
3	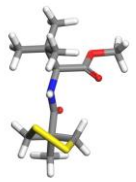	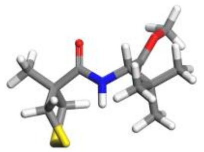	17%	*P* chirality<br>$\varphi_{C\text{-}S\text{-}S\text{-}C}$ = 44°	*l* = 2.53 Å<br>$\theta_{N\text{-}H\text{-}S}$ = 136°
4	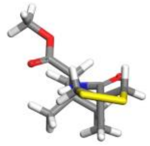	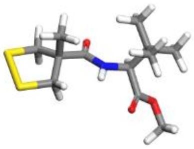	10%	*P* chirality<br>$\varphi_{C\text{-}S\text{-}S\text{-}C}$ = 15°	Not Observed
5	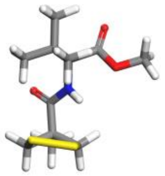	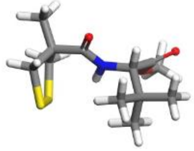	9%	*P* chirality<br>$\varphi_{C\text{-}S\text{-}S\text{-}C}$ = 11°	*l* = 2.52 Å<br>$\theta_{N\text{-}H\text{-}S}$ = 133°
6	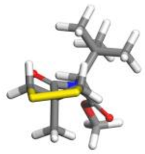	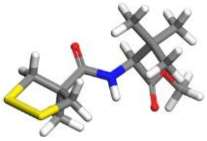	8%	*M* chirality<br>$\varphi_{C\text{-}S\text{-}S\text{-}C}$ = 12°	Not Observed
7	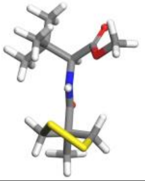	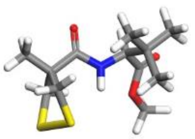	7%	*P* chirality<br>$\varphi_{C\text{-}S\text{-}S\text{-}C}$ = 42°	*l* = 2.56 Å<br>$\theta_{N\text{-}H\text{-}S}$ = 135°

Fig. S61.

DFT-optimized geometries in vacuo of the low-energy conformers of MAA-*L*-t-Ala, with their population percentage derived from Boltzmann averaging.

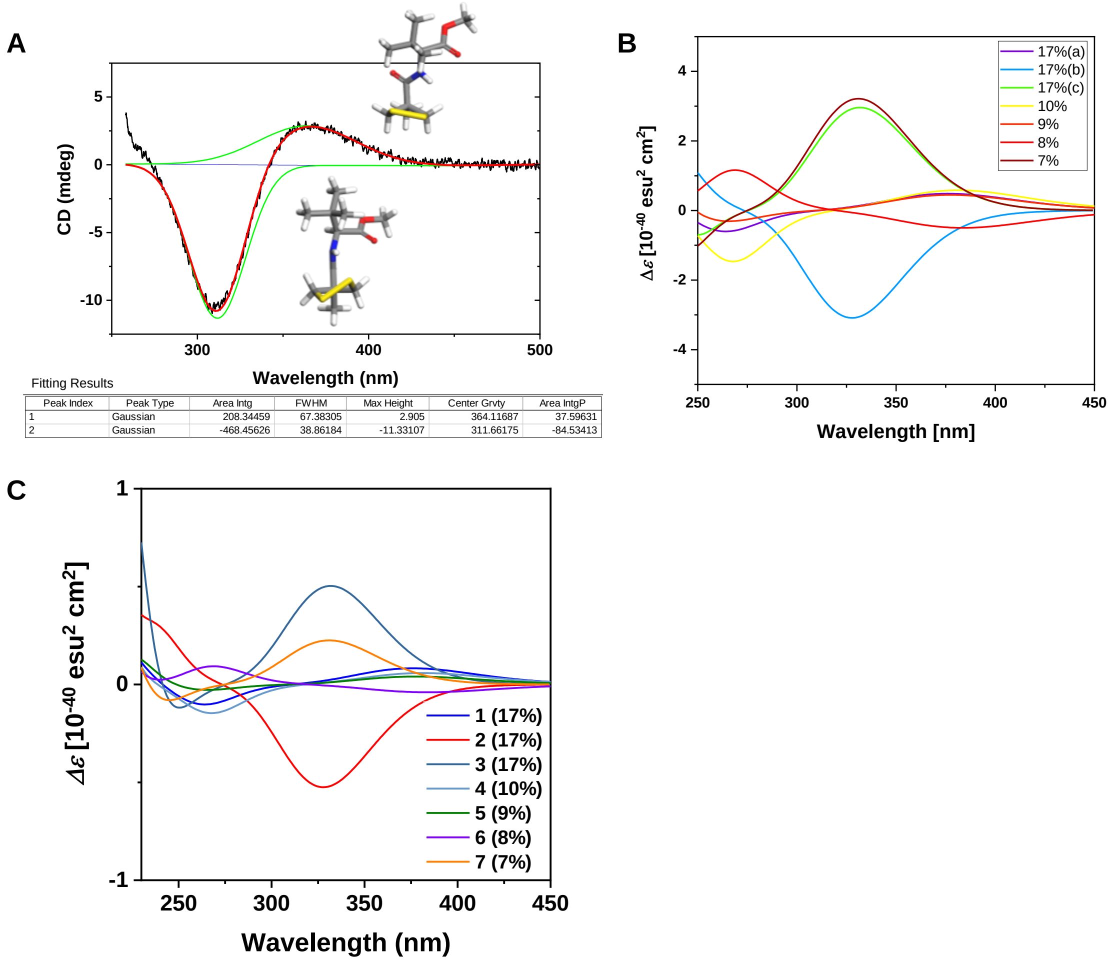

Fitting Results

Peak Index	Peak Type	Area Intg	FWHM	Max Height	Center Grvty	Area IntgP
1	Gaussian	208.34459	67.38305	2.905	364.11687	37.59631
2	Gaussian	-468.45626	38.86184	-11.33107	311.66175	-84.53413

Fig. S62.

(A) The peak analysis of the CD band of MAA-*L*-t-Leu in $CHCl_3$. Based on the calculated molar ellipticity in Fig. S62B, it can be estimated that the species of *P* helicity (CD band I at 364 nm) is the predominant species; (B) Simulated CD spectra of the different conformations of MAA-*L*-t-Leu as shown in Figure S61; (C) Simulated CD spectra of the different conformations of MAA-*L*-t-Leu as shown in Figure S61, with the intensities scaled using the Boltzmann averaged population.

	View A	View B	Population Percentage	S-S chirality/φ	S-S···H-N H-bond
1	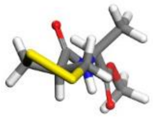	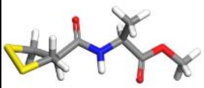	35%	***P*** **chirality** $\varphi_{C\text{-}S\text{-}S\text{-}C}$ = 40°	**Not Observed**
2	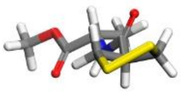	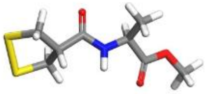	26%	***M*** **chirality** $\varphi_{C\text{-}S\text{-}S\text{-}C}$ = 40°	**Not Observed**
3	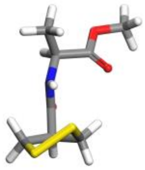	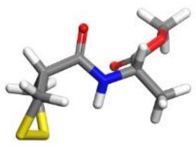	14%	***M*** **chirality** $\varphi_{C\text{-}S\text{-}S\text{-}C}$ = 43°	*l* = 2.59 Å $\theta_{N\text{-}H\text{-}S}$ = 137°
4	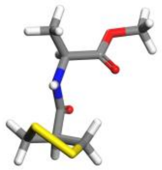	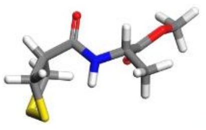	10%	***P*** **chirality** $\varphi_{C\text{-}S\text{-}S\text{-}C}$ = 43°	*l* = 2.60 Å $\theta_{N\text{-}H\text{-}S}$ = 135°
5	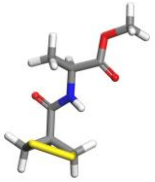	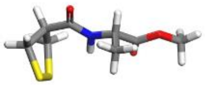	4%	***P*** **chirality** $\varphi_{C\text{-}S\text{-}S\text{-}C}$ = 17°	*l* = 2.70 Å $\theta_{N\text{-}H\text{-}S}$ = 129°
6	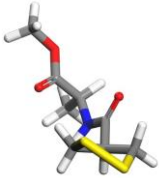	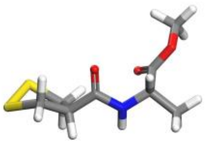	3%	***P*** **chirality** $\varphi_{C\text{-}S\text{-}S\text{-}C}$ = 45°	**Not Observed**
7	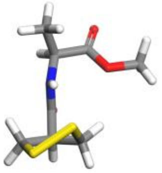	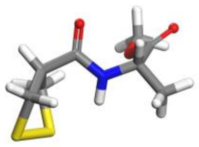	3%	***M*** **chirality** $\varphi_{C\text{-}S\text{-}S\text{-}C}$ = 42°	*l* = 2.60 Å $\theta_{N\text{-}H\text{-}S}$ = 135°

Fig. S63.

DFT-optimized geometries in vacuo of the low-energy conformers of AA-*L*-Ala, with their population percentage derived from Boltzmann averaging.

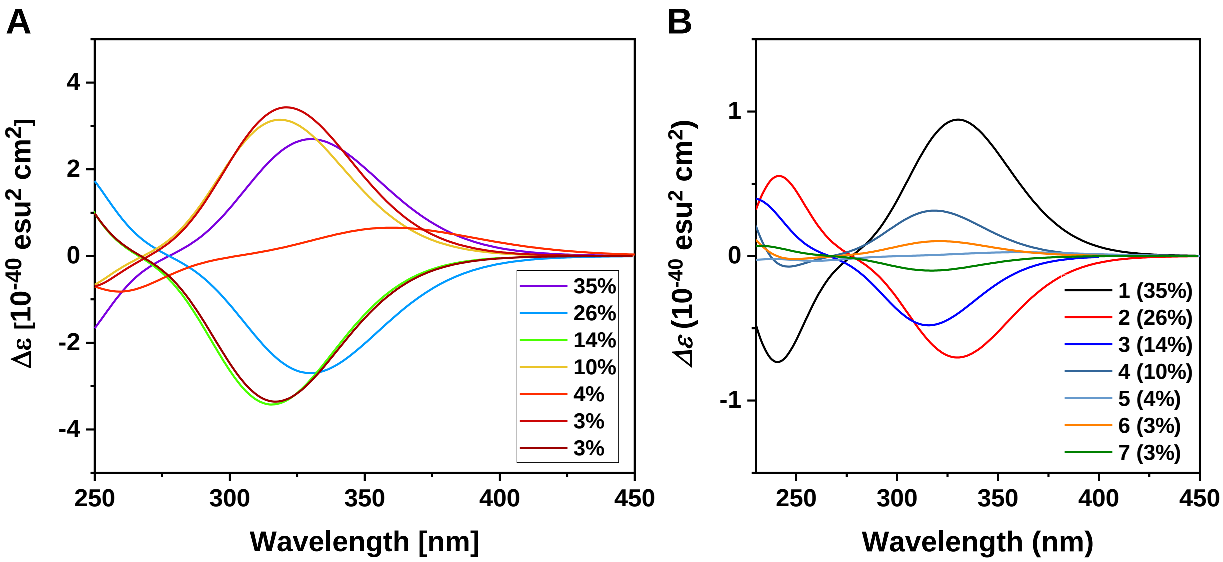

Fig. S64.

A. Simulated CD spectra of the different conformations of AA-*L*-Ala as shown in Figure S63.
B. Simulated CD spectra of the different conformations of AA-*L*-Ala as shown in Figure S63, with the intensities scaled using the Boltzmann averaged population.

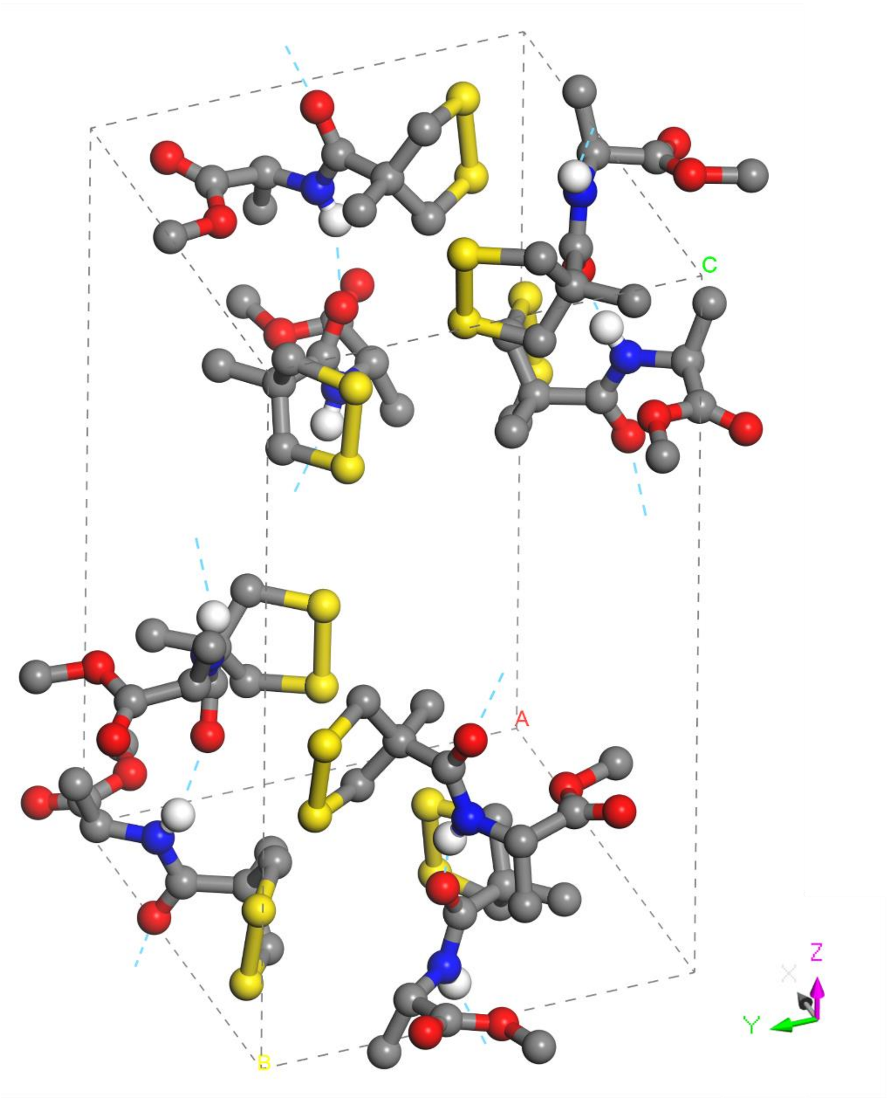

Fig. S65.

Unit cell of the crystal structure of MAA-*L*-Ala (CCDC deposition number: 2099418). Nonpolar hydrogen atoms are hidden for clarification.

Table S1.

Crystallographic data for MAA-*L*-Ala

chem formula	C9 H15 N O3 S2
Mr	249.34
cryst syst	tetragonal
color, habit	yellow, needle
size (mm)	0.363 x 0.117 x 0.030
space group	P 41 21 2
a (Å)	10.9276(5)
b (Å)	10.9276(5)
c (Å)	19.6062(13)
α, deg	90
β, deg	90
γ, deg	90
V (Å3)	2341.2(3)
Z	8
ρ_{calc}, g.cm^{-3}	1.415
μ/mm^{-1}	4.047
F(000)	1056
temp (K)	100(2)
θ range (deg)	4.632 - 68.243
data collected (h,k,l)	-13:13, -13:13, -21:23
no. of rflns collected	35754
no. of indpndt reflns	2135
observed reflns	2110 ($F_o \geq 2\ \sigma(F_o)$)
R(F) (%)	1.99
wR(F^2) (%)	5.3
GooF	1.098
Weighting a,b	0.0261, 0.6210
params refined	139
restraints	0
min, max resid dens	-0.185, 0.171
Flack x	0.015(4)

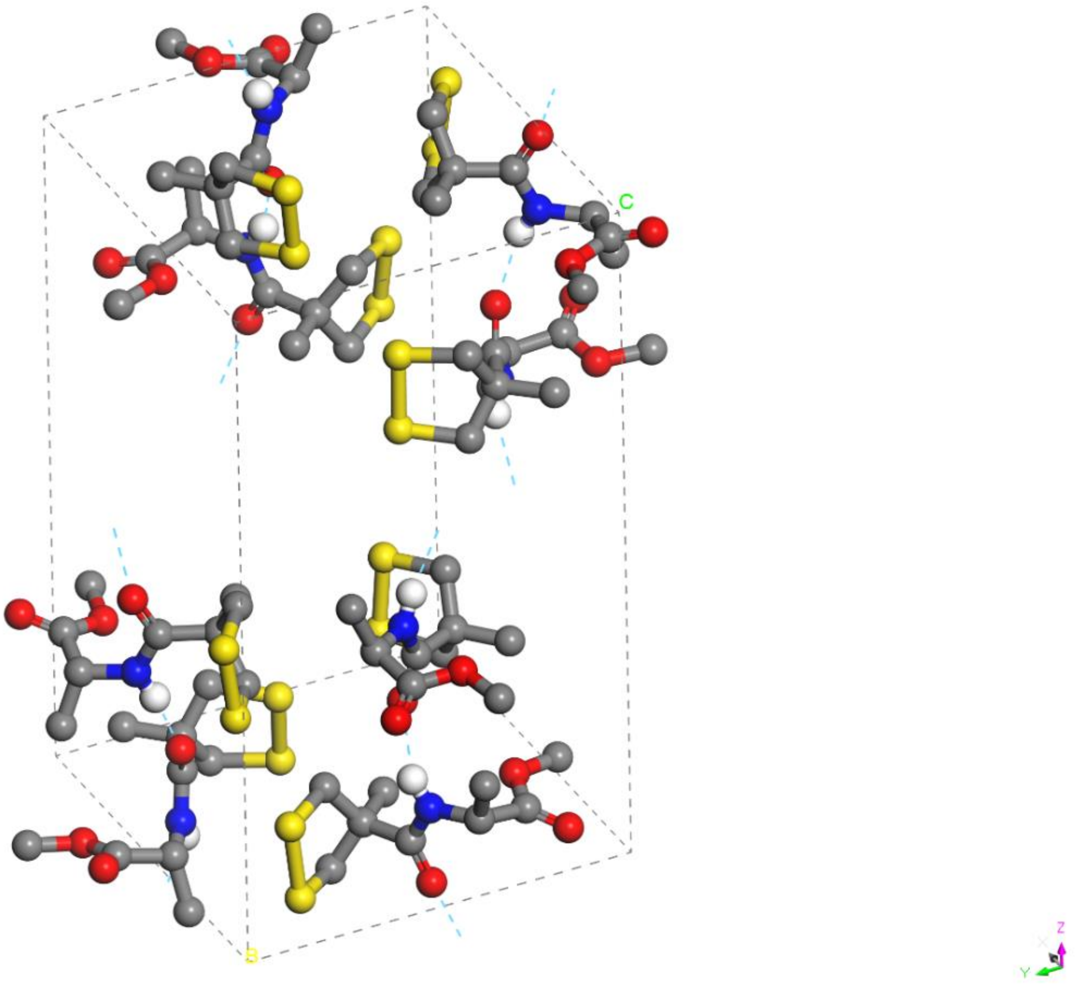

Fig. S66.

Unit cell of the crystal structure of MAA-*D*-Ala (CCDC deposition number: 2099417). Nonpolar hydrogen atoms are hidden for clarification.

Table S2.

Crystallographic data for MAA-*D*-Ala

chem formula	C9 H15 N O3 S2
Mr	249.34
cryst syst	tetragonal
color, habit	yellow, needle
size (mm)	0.224 × 0.041 × 0.04
space group	$P4_32_12$
a (Å)	10.9588(7)
b (Å)	10.9588(7)
c (Å)	19.5173(16)
α, deg	90
β, deg	90
γ, deg	90
V (Å3)	2343.9(4)
Z	8
ρ_{calc}, g.cm^{-3}	1.413
μ/mm^{-1}	4.042
F(000)	1056.0
Radiation	CuKα (λ = 1.54178)
2Θ range for data collection/°	9.254 to 139.644
Index ranges	$-13 \le h \le 13$, $-13 \le k \le 13$, $-22 \le l \le 23$
Reflections collected	30101
Independent reflections	2144 [R_{int} = 0.1410, R_{sigma} = 0.0742]
Data/restraints/parameters	2144/0/140
Goodness-of-fit on F^2	1.091
Final R indexes [I>=2σ (I)]	R_1 = 0.0641, wR_2 = 0.0901
Final R indexes [all data]	R_1 = 0.1037, wR_2 = 0.1054
Largest diff. peak/hole / e Å^{-3}	0.48/-0.34
Flack parameter	0.039(15)

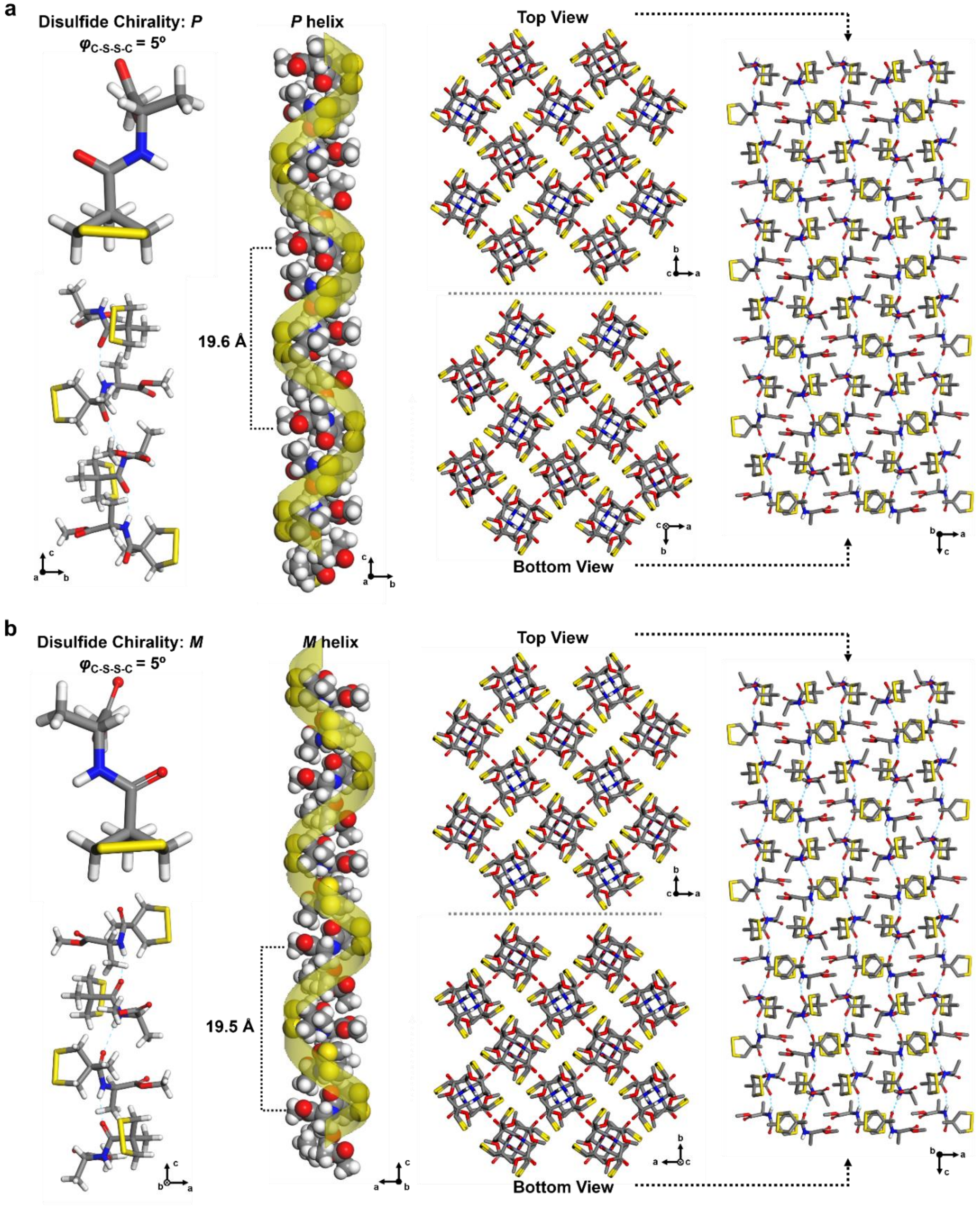

Fig. S67.

Comparison of X-ray single-crystal structures of MAA-*L*-Ala and MAA-*D*-Ala.

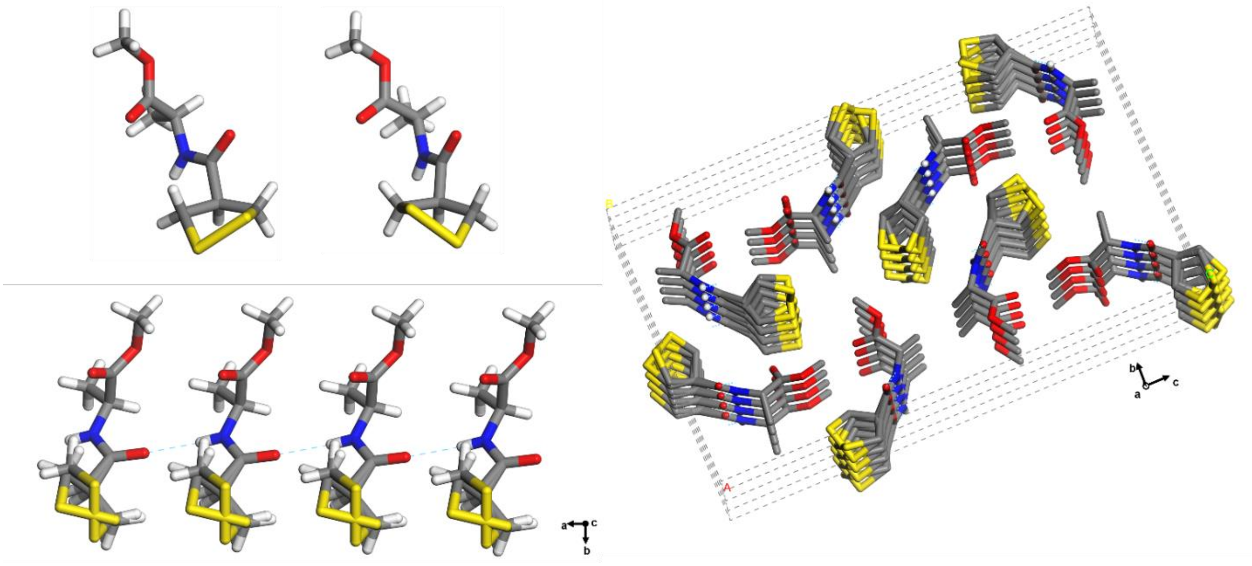

Fig. S68.
X-ray single crystal analysis of AA-*L*-Ala (CCDC deposition number: 2099415).

Table S3.

Crystallographic data for AA-*L*-Ala

chem formula	$C_8H_{13}NO_3S_2$
Mr	235.31
cryst syst	orthorhombic
color, habit	colourless, block
size (mm)	0.360 × 0.082 × 0.009
space group	P 21 21 21
a (Å)	4.87210(10)
b (Å)	15.8579(3)
c (Å)	27.9770(6)
α, deg	90
β, deg	90
γ, deg	90
V (Å3)	2161.54(8)
Z	8
ρ_{calc}, g.cm^{-3}	1.446
μ/mm^{-1}	4.350
F(000)	992
temp (K)	100(2)
θ range (deg)	3.159 - 72.140
data collected (h,k,l)	-5:5, -18:18, -34:31
no. of rflns collected	12824
no. of indpndt reflns	4156
observed reflns	3868 ($F_o \geq 2\ \sigma(F_o)$)
R(F) (%)	2.94
wR(F^2) (%)	7.21
GooF	1.050
Weighting a,b	0.0351 0.2444
params refined	359
restraints	428
min, max resid dens	-0.225, 0.239
Flack x	0.014(7)

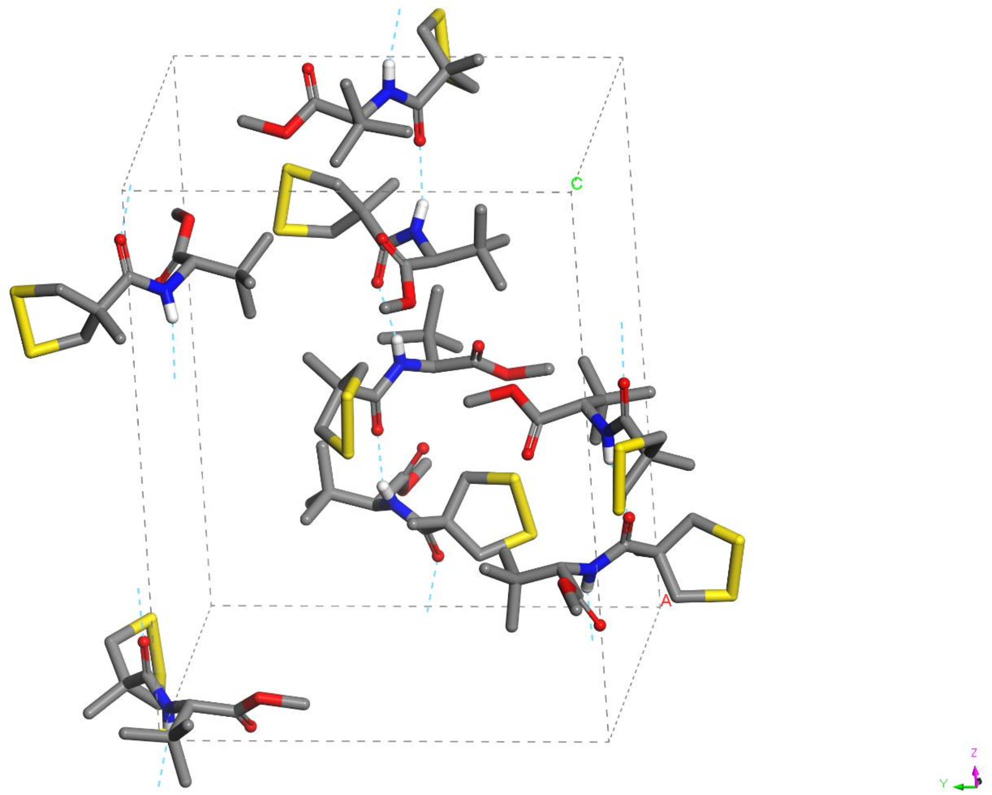

Fig. S69.

Unit cell of the crystal structure of MAA-*L*-t-Leu (CCDC deposition number: 2099416). Nonpolar hydrogen atoms are hidden for clarification.

Table S4.
Crystallographic data for MAA-*L*-t-Leu

chem formula	$C_{12}H_{21}NO_3S_2$
Mr	291.42
cryst syst	orthorhombic
color, habit	yellow, block
size (mm)	0.408 × 0.266 × 0.212
space group	$P2_12_12_1$
a (Å)	10.7966(5)
b (Å)	14.5828(7)
c (Å)	19.2118(9)
α, deg	90
β, deg	90
γ, deg	90
V (Å3)	3024.8(2)
Z	8
ρ_{calc}, g.cm^{-3}	1.280
μ/mm^{-1}	3.205
F(000)	1248.0
Radiation	CuKα (λ = 1.54178)
2Θ range for data collection/°	7.61 to 137.28
Index ranges	-13 ⩽ h ⩽ 13, -17 ⩽ k ⩽ 17, -23 ⩽ l ⩽ 23
Reflections collected	86820
Independent reflections	5533 [R_{int} = 0.0597, R_{sigma} = 0.0226]
Data/restraints/parameters	5533/0/336
Goodness-of-fit on F^2	1.050
Final R indexes [I>=2σ (I)]	R_1 = 0.0232, wR_2 = 0.0578
Final R indexes [all data]	R_1 = 0.0242, wR_2 = 0.0583
Largest diff. peak/hole / e Å^{-3}	0.25/-0.23
Flack parameter	0.018(4)

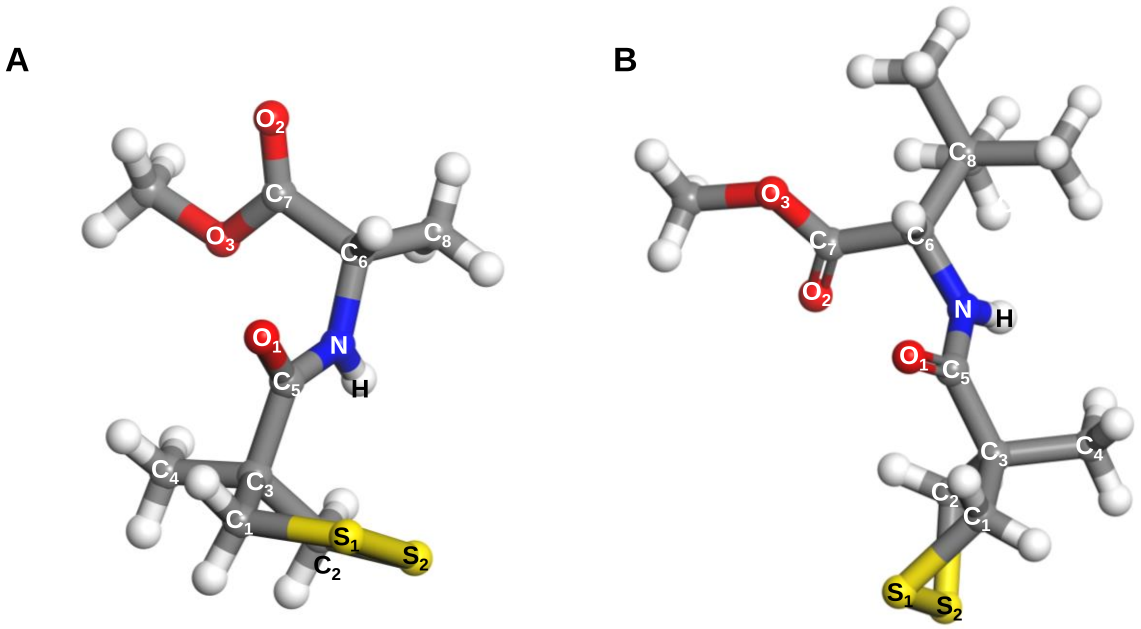

C

	MAA-L-Ala	MAA-L-t-Leu
**θ (N-C_6-C_7)**	**112.3°**	**107.6°**
**θ (C_4-C_3-C_5)**	**107.4°**	**110.9°**
**θ (C_1-C_3-C_2)**	**105.5°**	**109.8°**
**Torsion φ (N-H~C_6-C_8)**	**16.0°**	**28.3°**
**Torsion φ (C_1-S_1~C_2-S_2)**	**4.7°**	**46.7°**
**Inter-H-bond length**	**2.09 Å**	**2.14 Å**

Fig. S70.
Molecular geometry comparison of MAA-L-Ala (A) and MAA-L-t-Leu (B) in single crystals; Summary (C) of the distinctive parameters measured from the crystal structure.

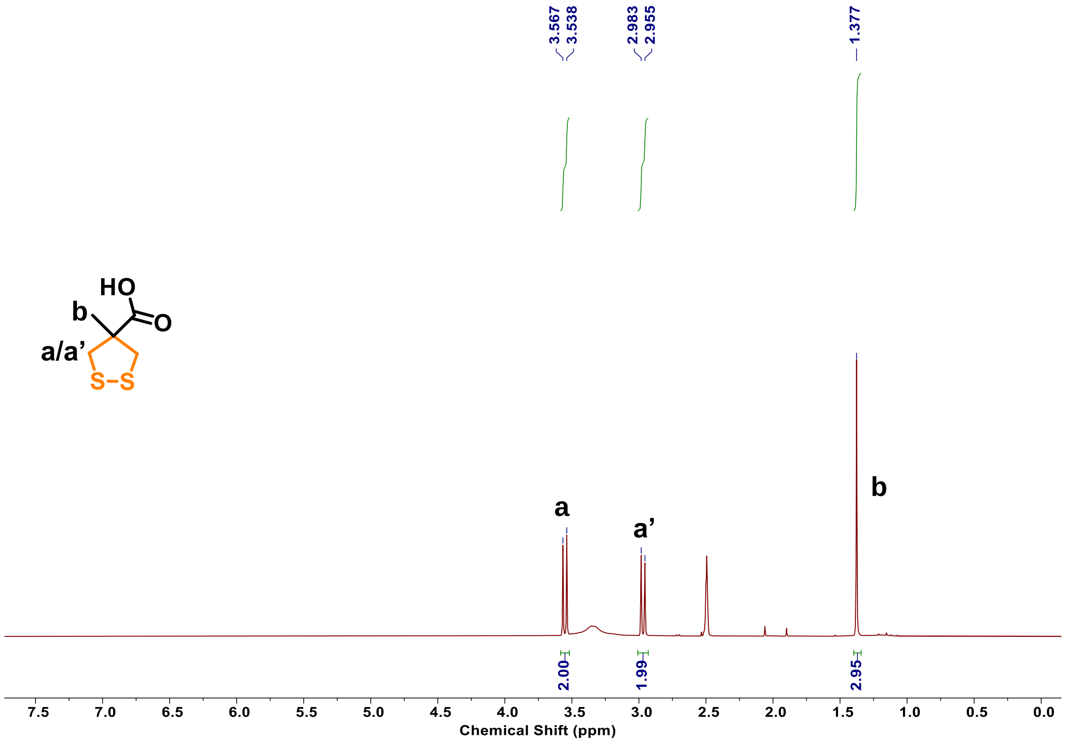

Fig. S71. ^{1}H NMR spectrum of MAA in d_6-DMSO (400 MHz, 298K).

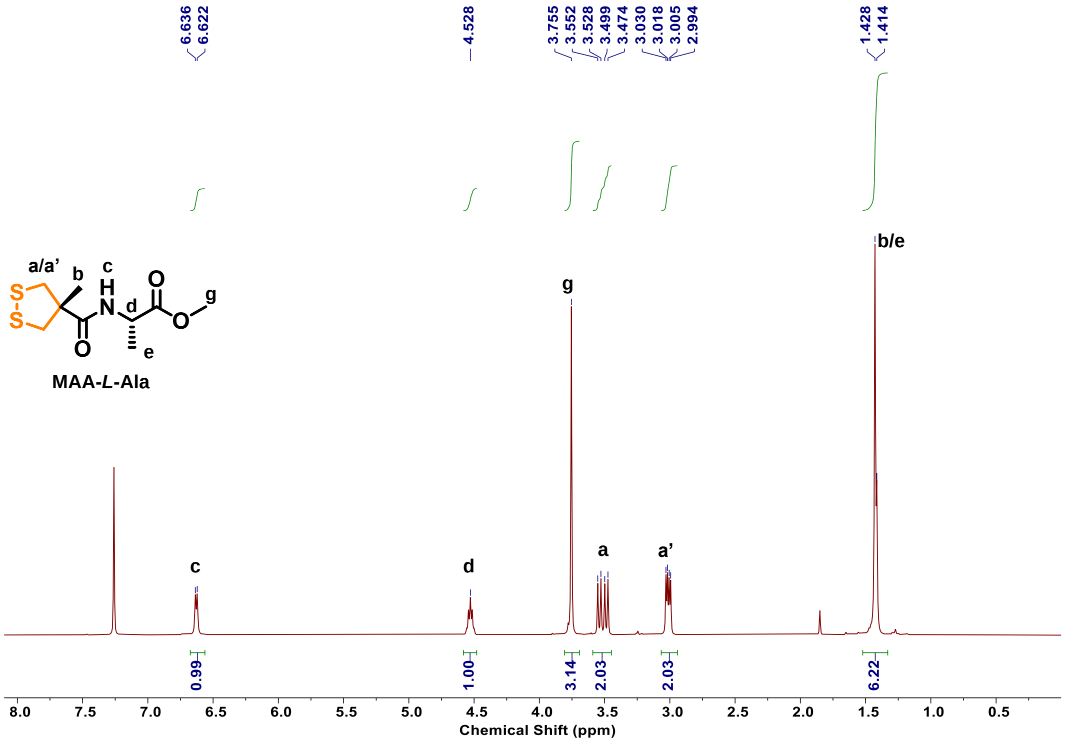

Fig. S72.

^{1}H NMR spectrum of MAA-*L*-Ala in $CDCl_3$ (500 MHz, 233K).

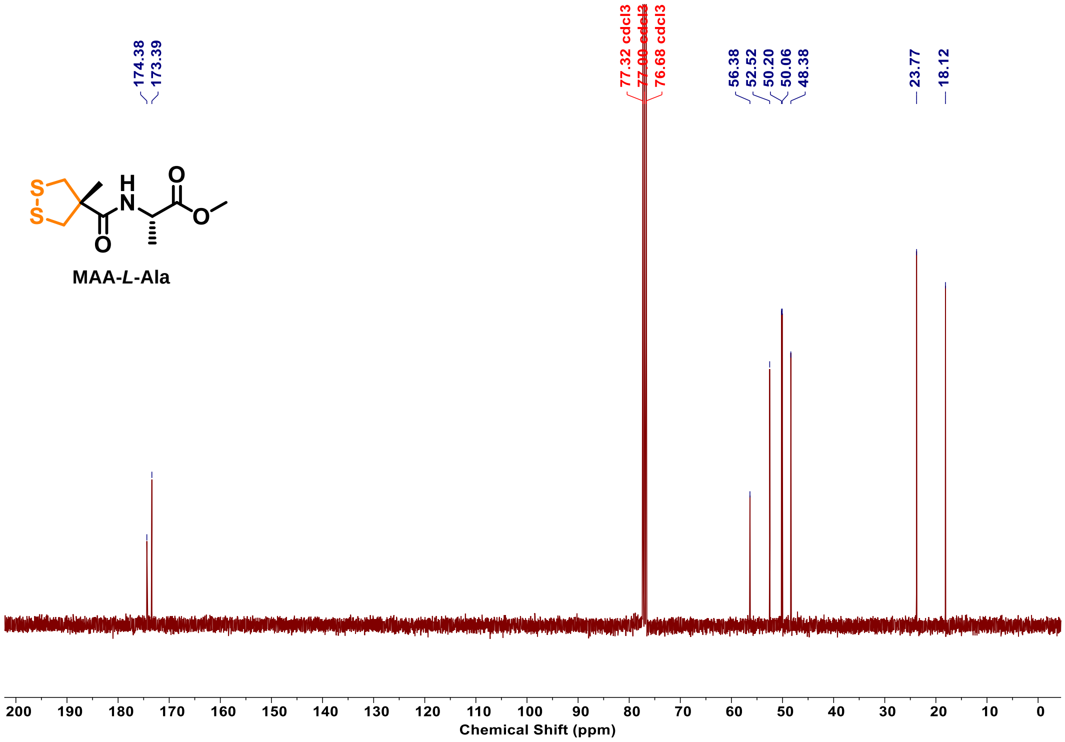

Fig. S73.

^{13}C NMR spectrum of MAA-*L*-Ala in $CDCl_3$ (101 MHz, 298K).

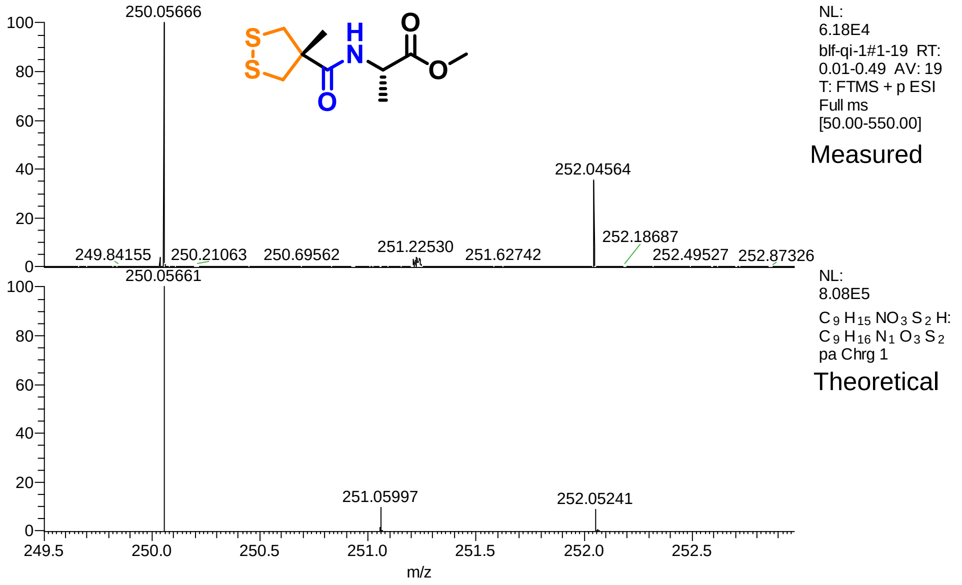

Fig. S74.

HR-MS of MAA-*L*-Ala (Measured: 250.0567; Calculated: 250.0566).

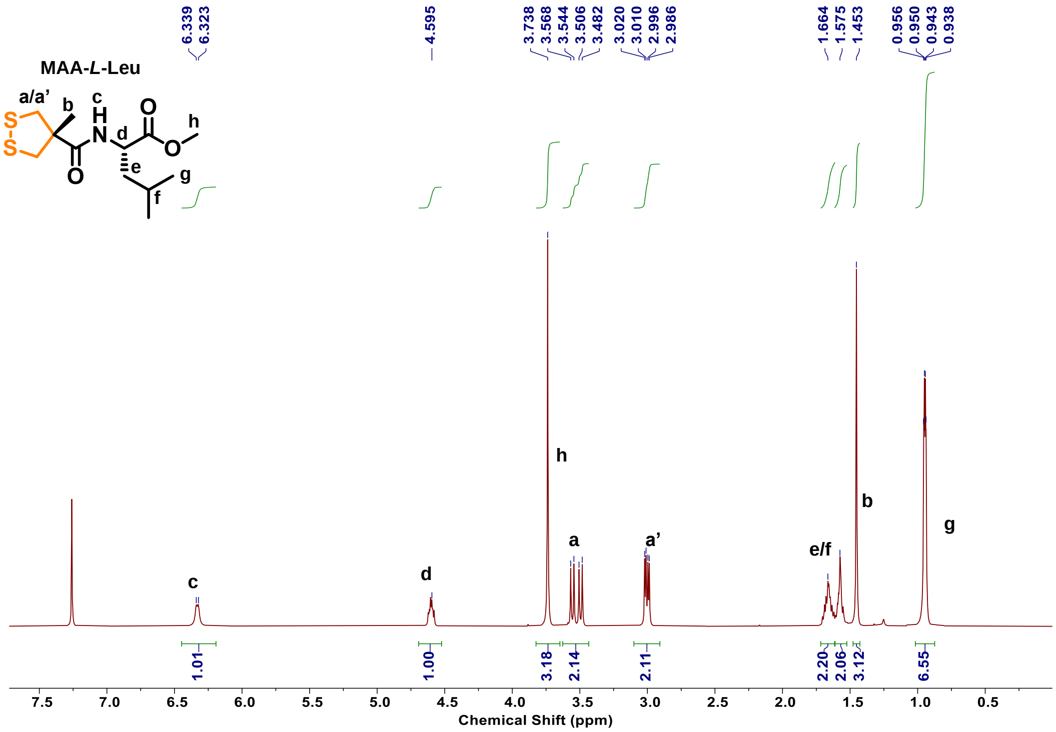

Fig. S75.

^{1}H NMR spectrum of MAA-*L*-Leu in $CDCl_3$ (500 MHz, 298K).

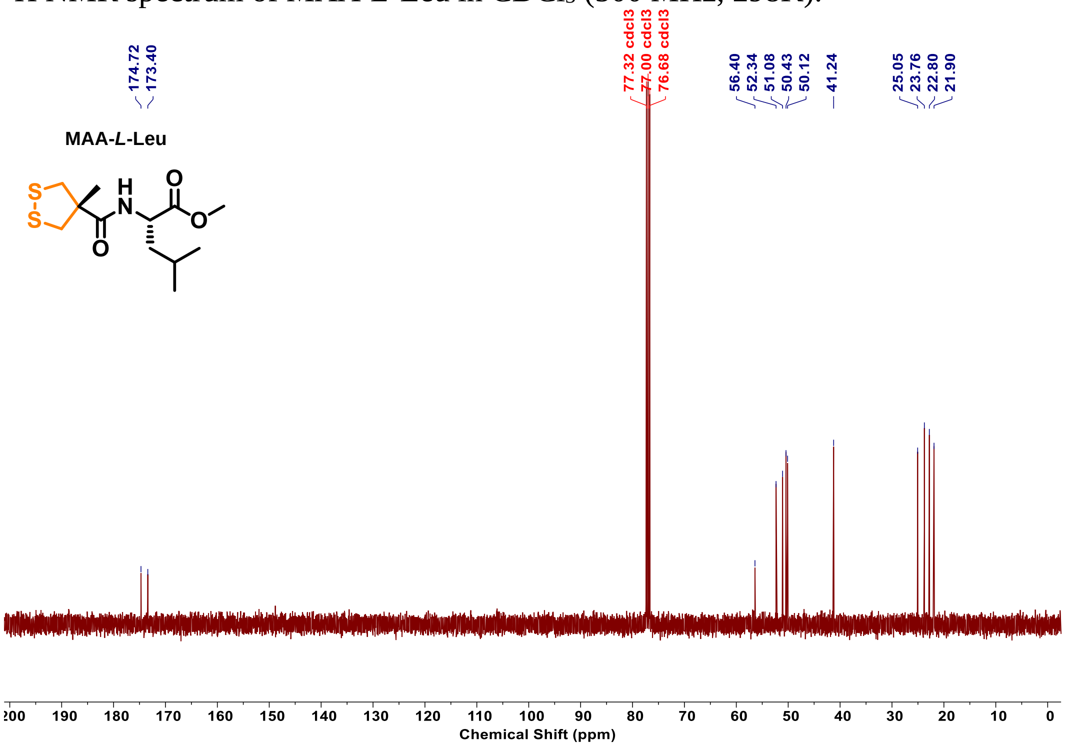

Fig. S76.

^{13}C NMR spectrum of MAA-*L*-Leu in $CDCl_3$ (101 MHz, 298K).

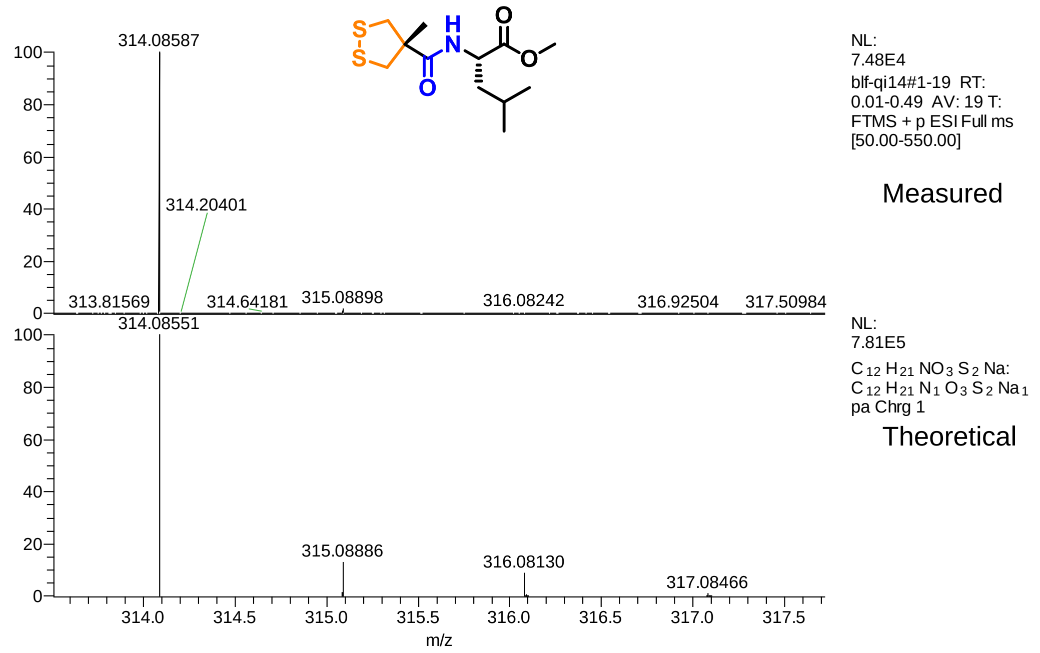

Fig. S77.

HRMS of MAA-*L*-Leu (Measured: 314.0859; Calculated: 314.0855).

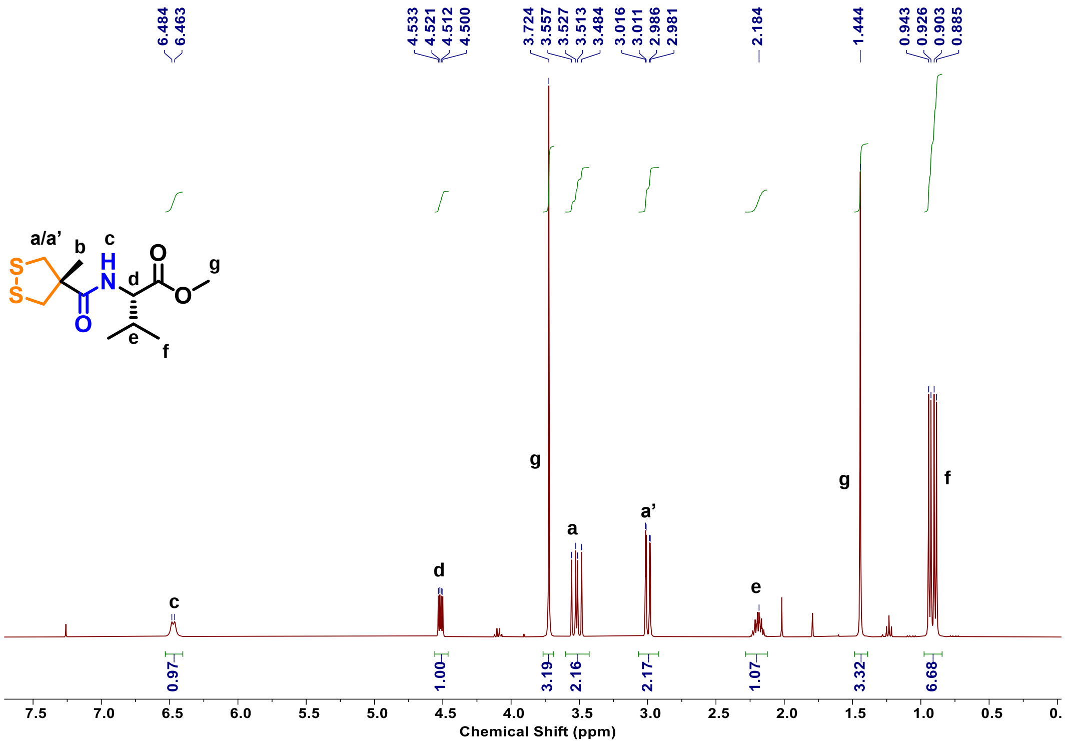

Fig. S78.

^{1}H NMR spectrum of MAA-*L*-Val in $CDCl_3$ (400 MHz, 298K).

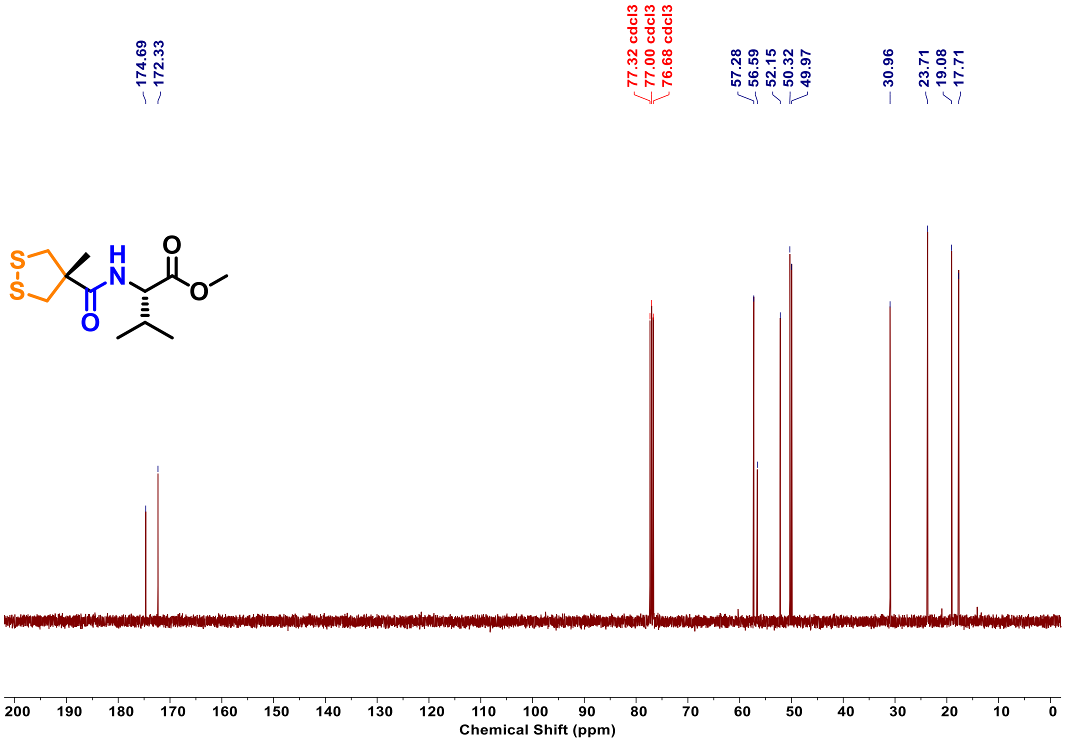

Fig. S79.

^{13}C NMR spectrum of MAA-*L*-Val in $CDCl_3$ (101 MHz, 298K).

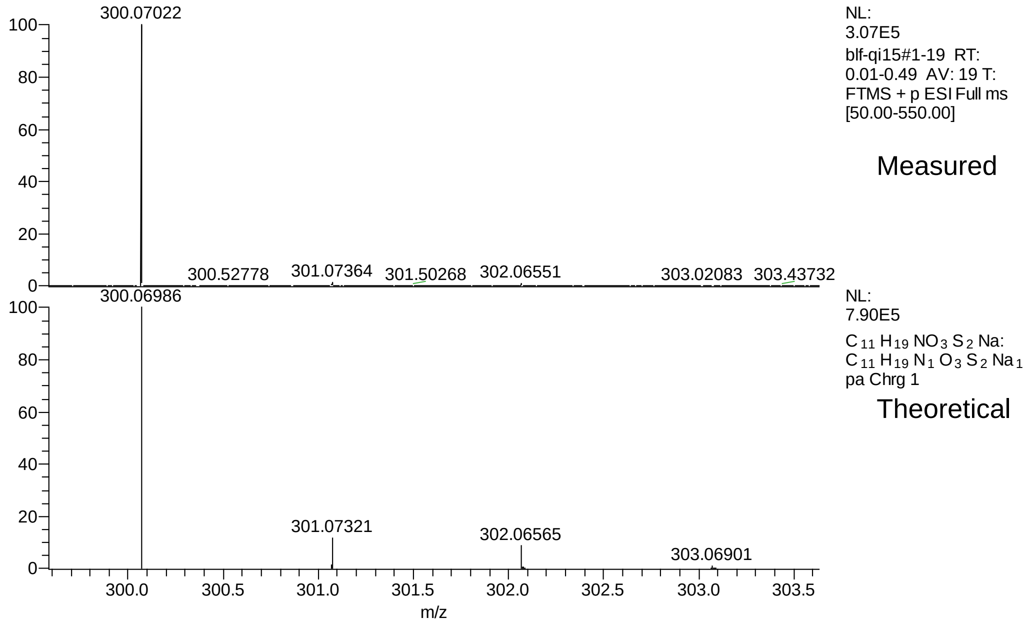

Fig. S80.

HRMS of MAA-*L*-Val (Measured: 314.0859; Calculated: 314.0855).

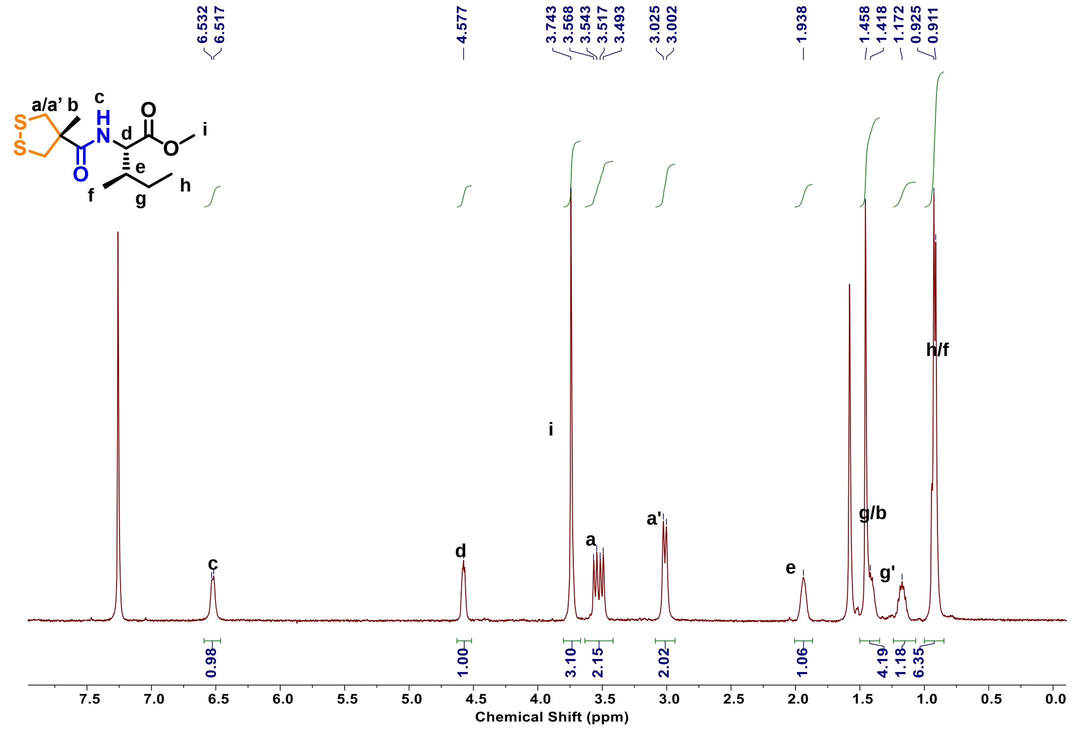

Fig. S81.

^{1}H NMR spectrum of MAA-*L*-i-Leu in $CDCl_3$ (500 MHz, 293K).

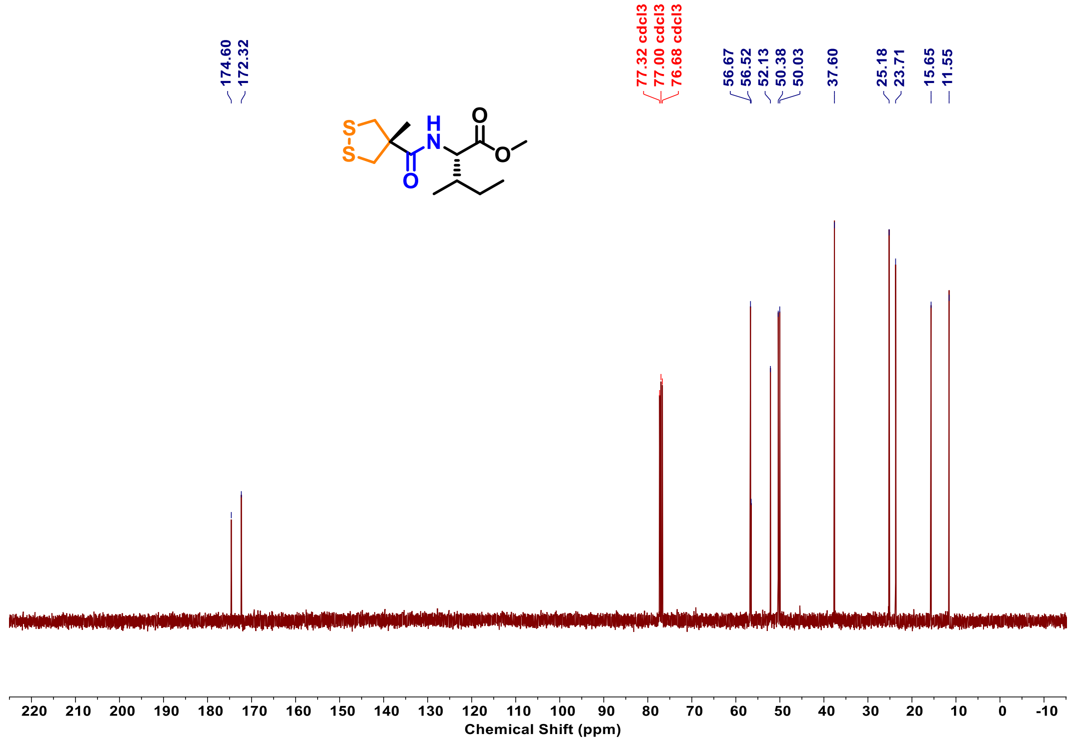

Fig. S82.

^{13}C NMR spectrum of MAA-*L*-i-Leu in $CDCl_3$ (101 MHz, 298K).

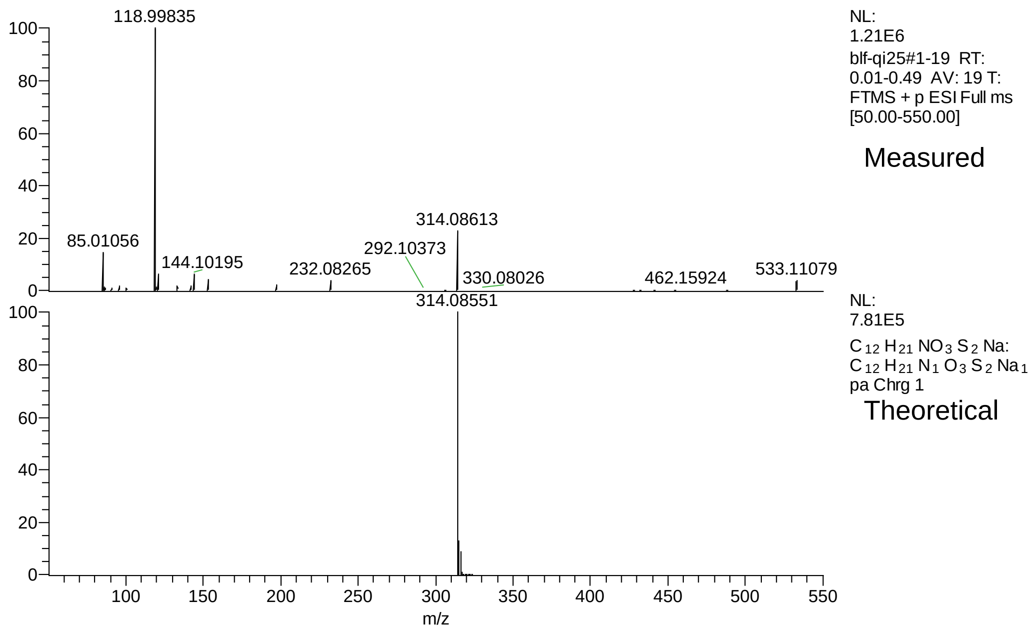

Fig. S83.

HR-MS of MAA-*L*-i-Leu (Measured: 314.0861; Calculated: 314.0855).

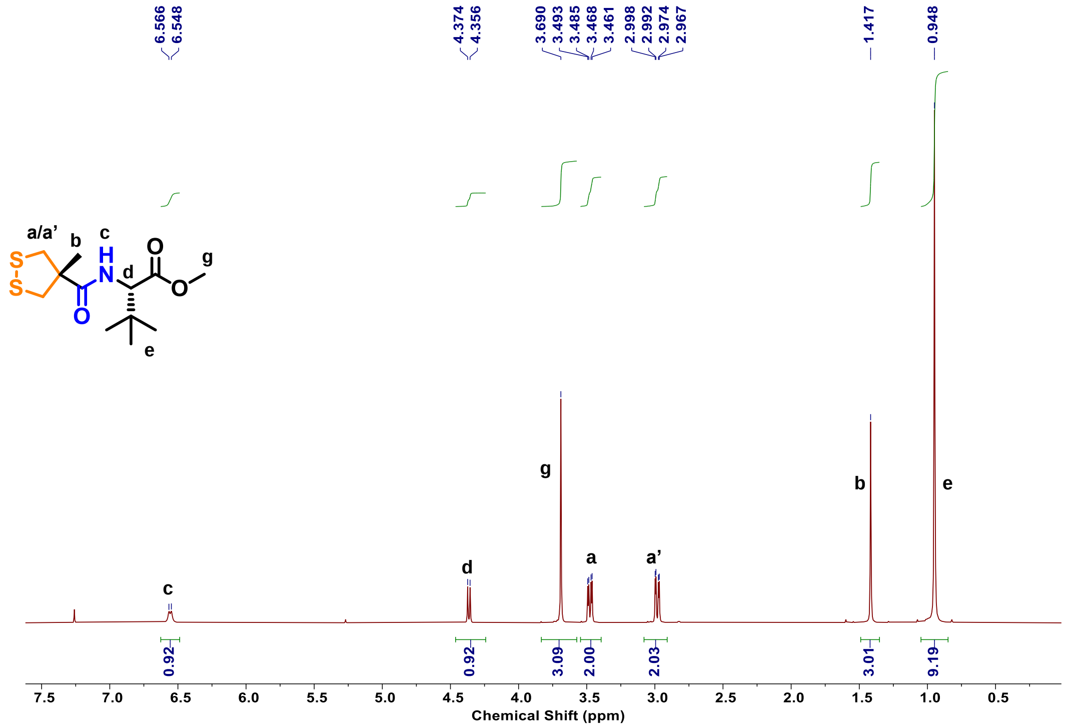

Fig. S84.

^{1}H NMR spectrum of MAA-*L*-t-Leu in $CDCl_3$ (500 MHz, 293K).

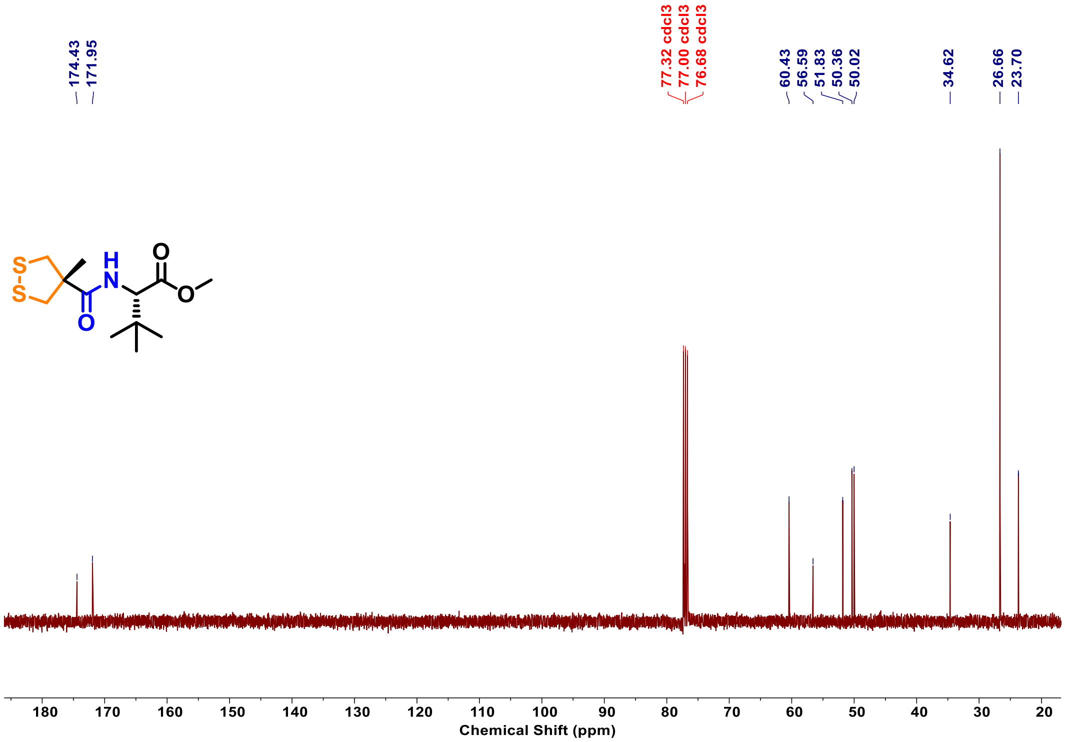

Fig. S85.

^{13}C NMR spectrum of MAA-*L*-t-Leu in $CDCl_3$ (101 MHz, 298K).

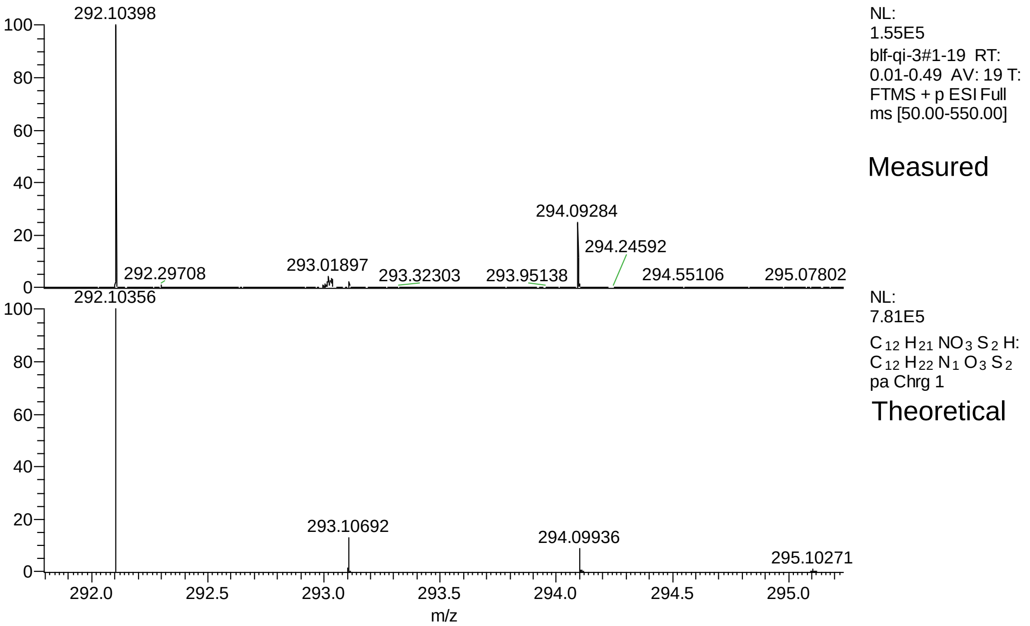

Fig. S86.

HR-MS of MAA-*L*-t-Leu (Measured: 292.1040; Calculated: 292.1036).

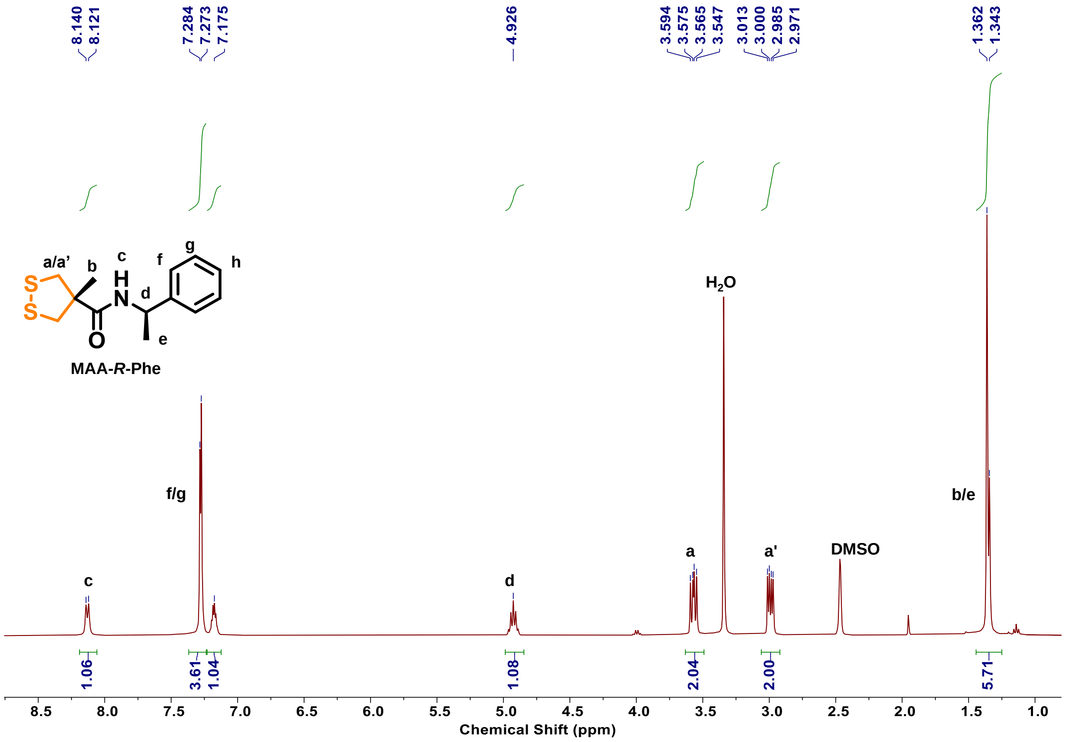

Fig. S87.

^{1}H NMR spectrum of MAA-*R*-Phe in d_6-DMSO (400 MHz, 298K).

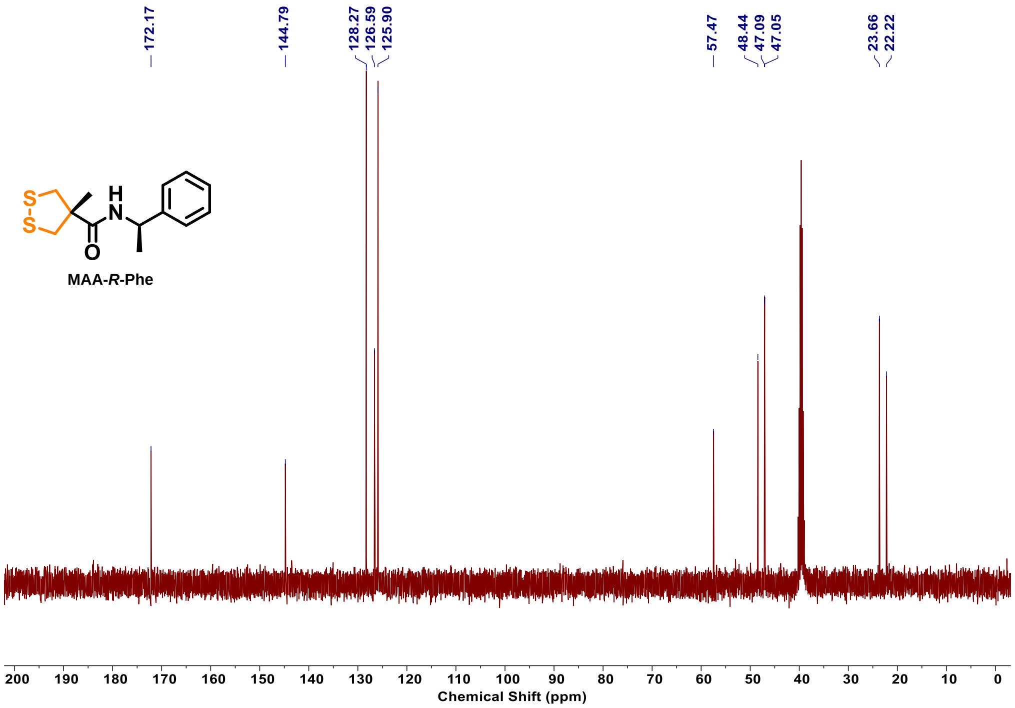

Fig. S88.

^{13}C NMR spectrum of MAA-*R*-Phe in d_6-DMSO (101 MHz, 298K).

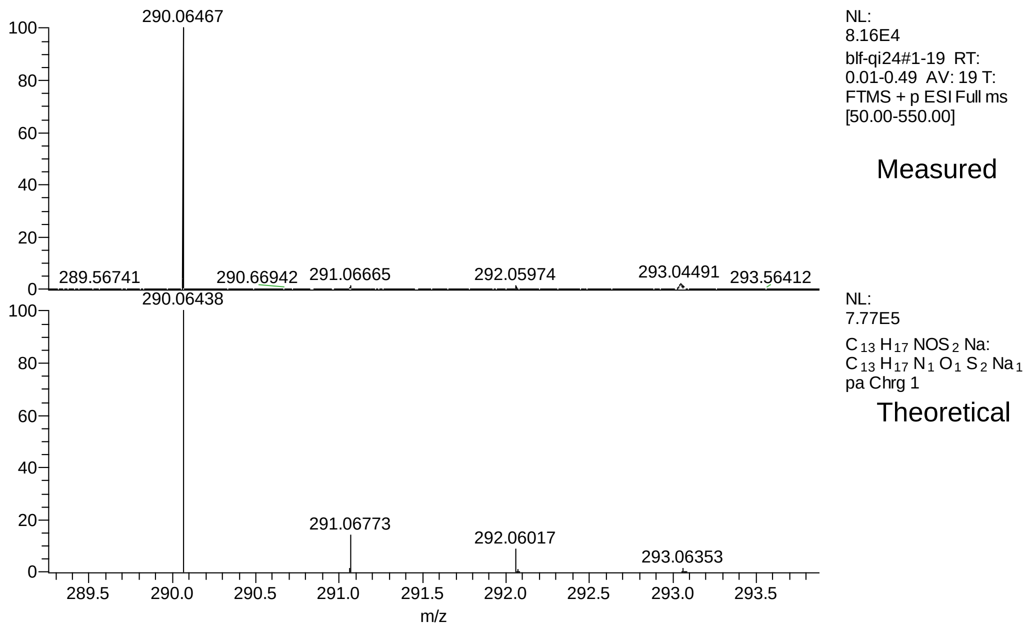

Fig. S89.

HR-MS of MAA-*R*-Phe (Measured: 290.0647; Calculated: 290.0644)

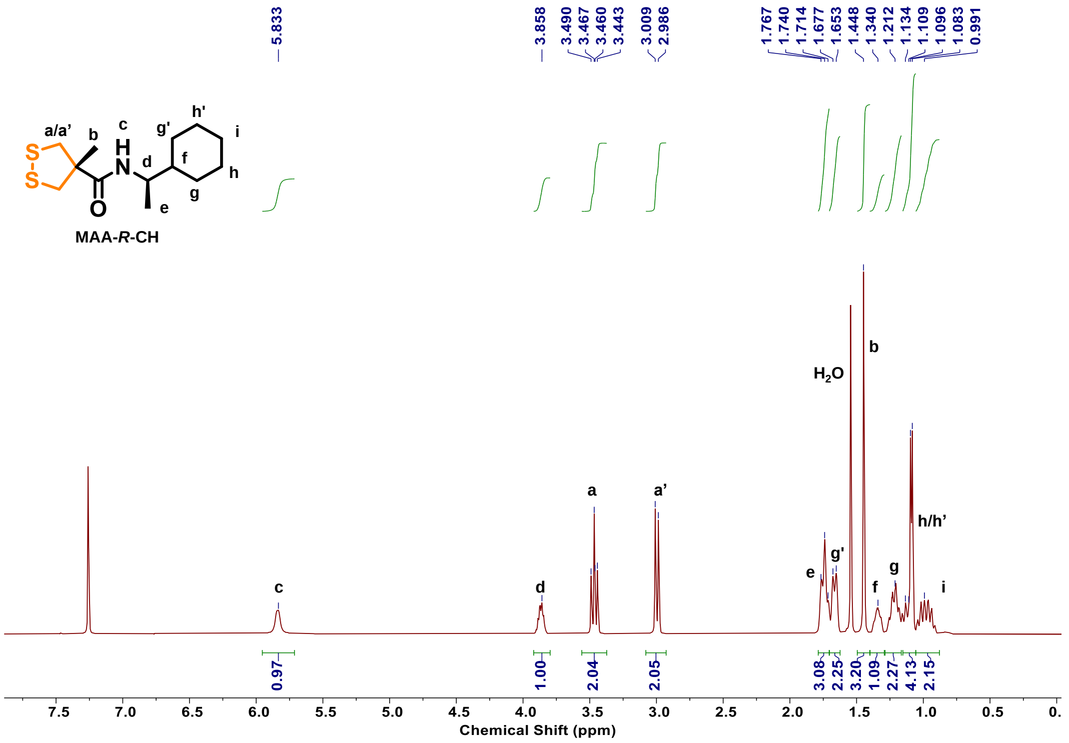

Fig. S90.

^{1}H NMR spectrum of MAA-*R*-CH in $CDCl_3$ (500 MHz, 293K).

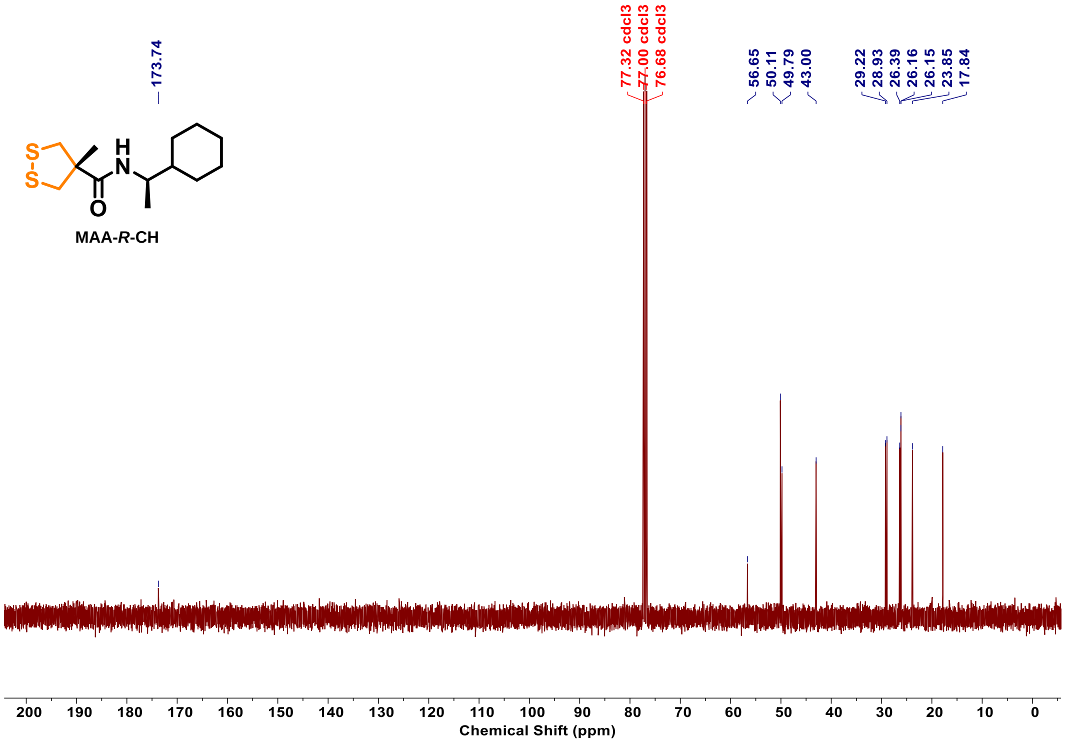

Fig. S91

^{13}C NMR spectrum of MAA-*R*-CH in $CDCl_3$ (101 MHz, 298K).

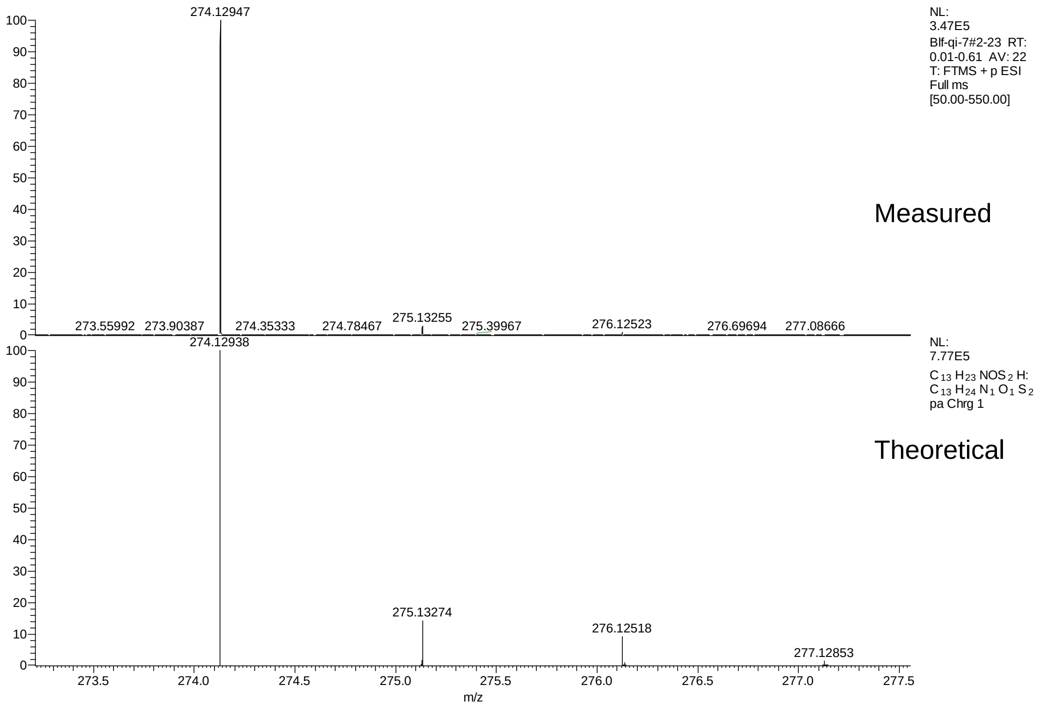

Fig. S92.

HR-MS of MAA-*R*-CH (Measured: 274.1295; Calculated: 274.1294)

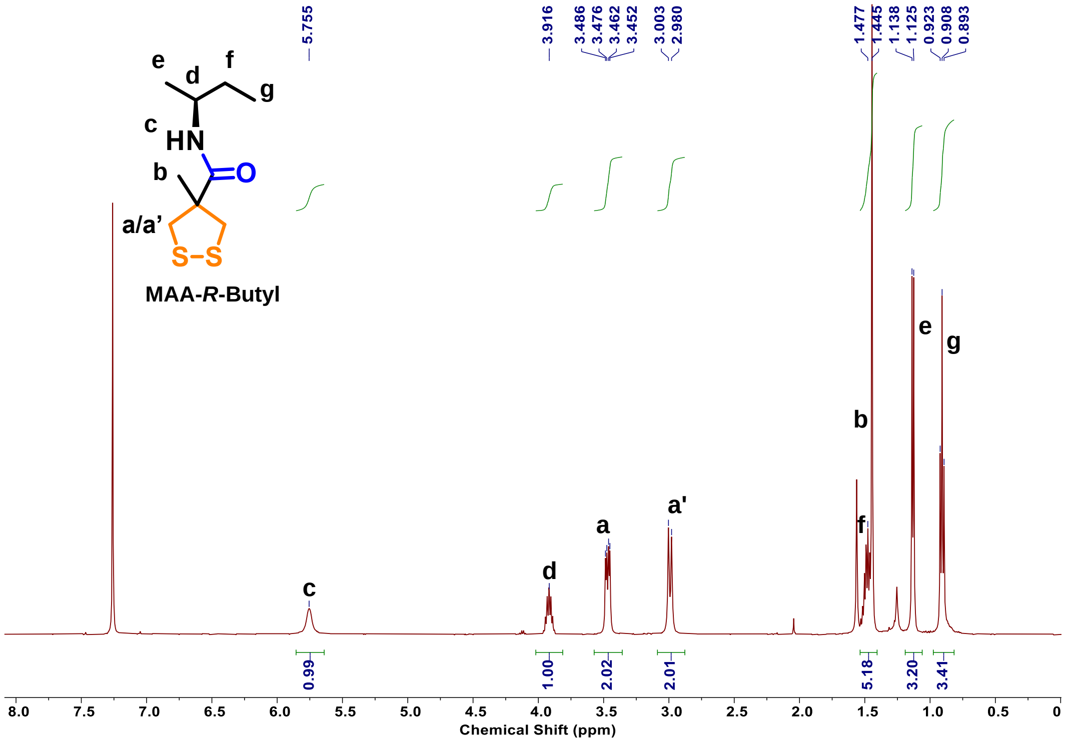

Fig. S93.

^{1}H NMR spectrum of MAA-*R*-Butyl in $CDCl_3$ (500 MHz, 298K).

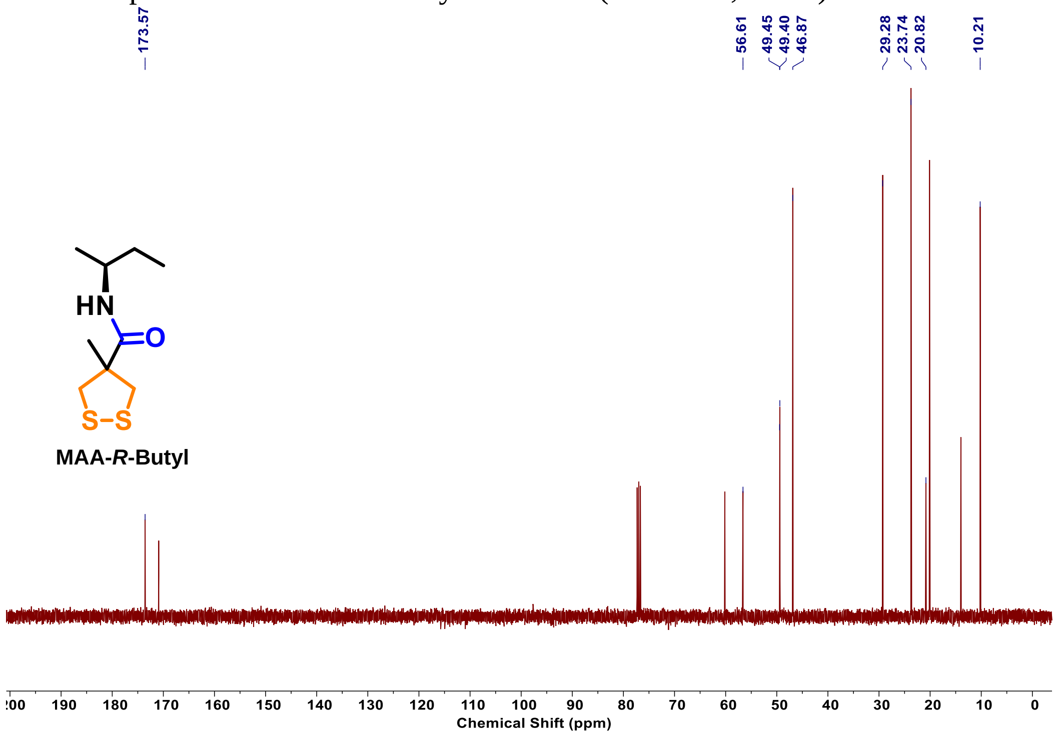

Fig. S94.

^{13}C NMR spectrum of MAA-*R*-Butyl in $CDCl_3$ (101 MHz, 298K).

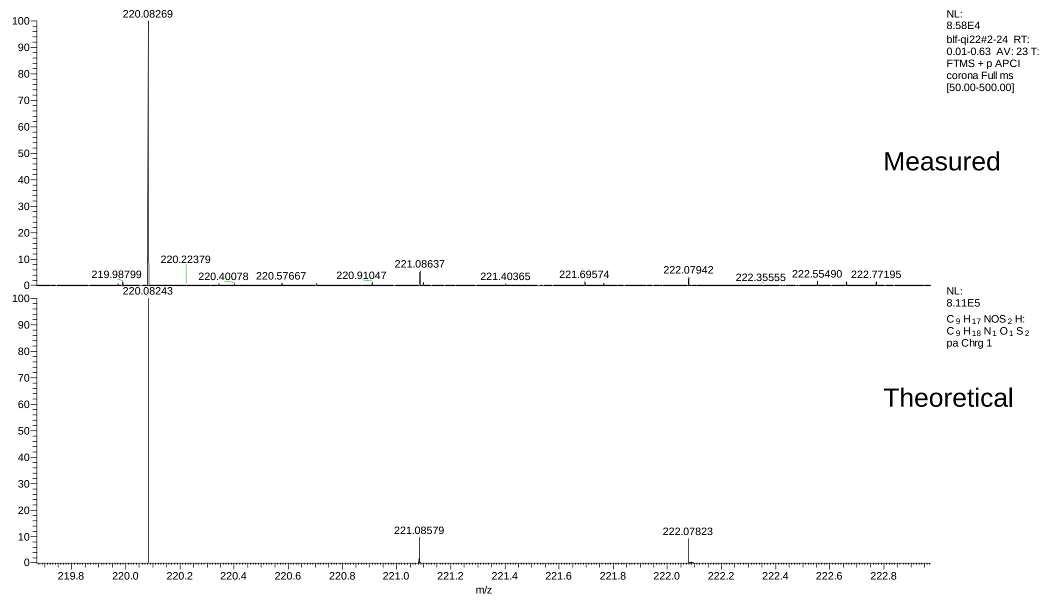

Fig. S95.

HR-MS of MAA-R-Butyl (Measured: 220.0827; Calculated: 220.0824)

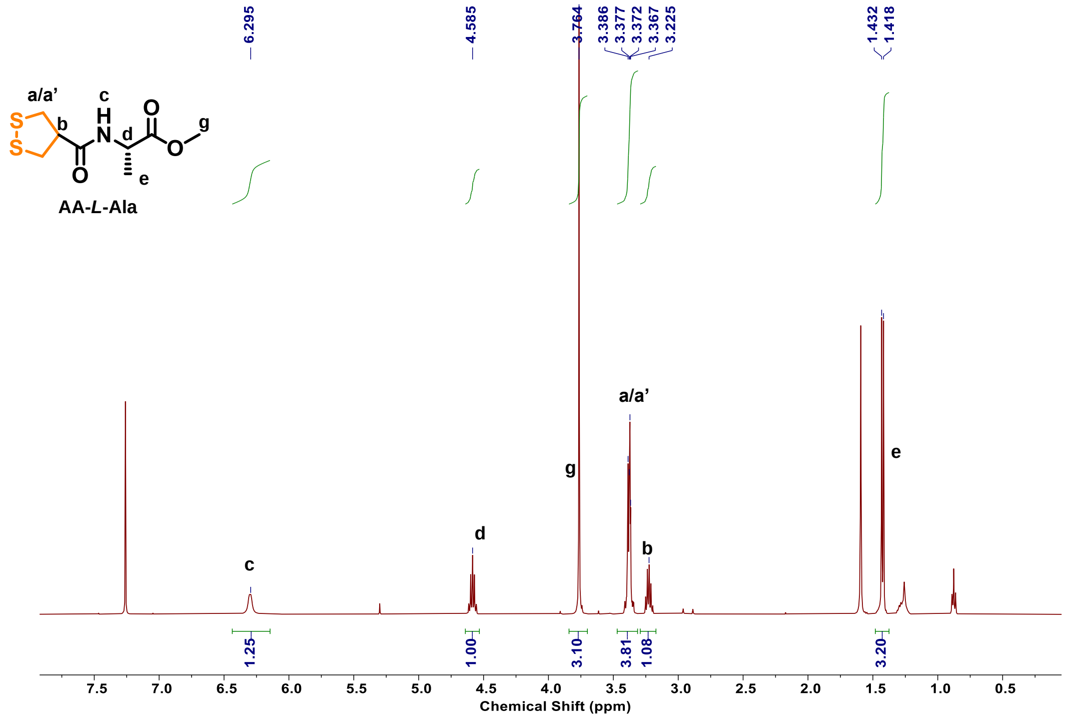

Fig. S96.

^{1}H NMR spectrum of AA-*L*-Ala in $CDCl_3$ (500 MHz, 293K).

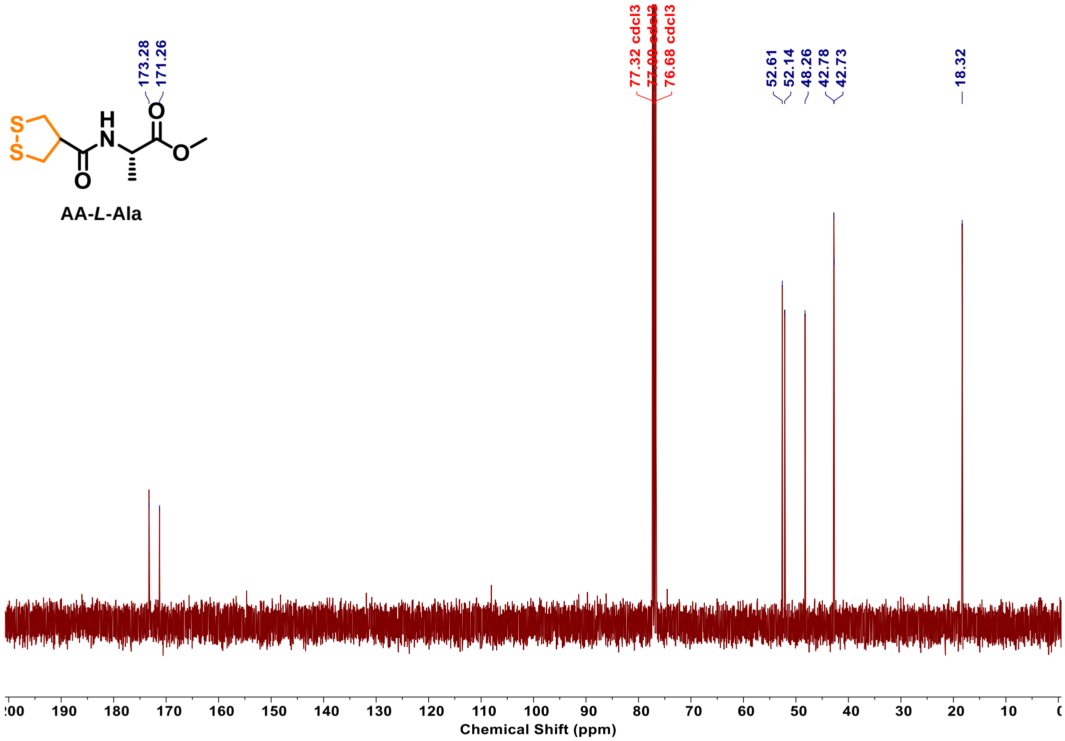

Fig. S97.

^{13}C NMR spectrum of AA-*L*-Ala in $CDCl_3$ (101 MHz, 298K).

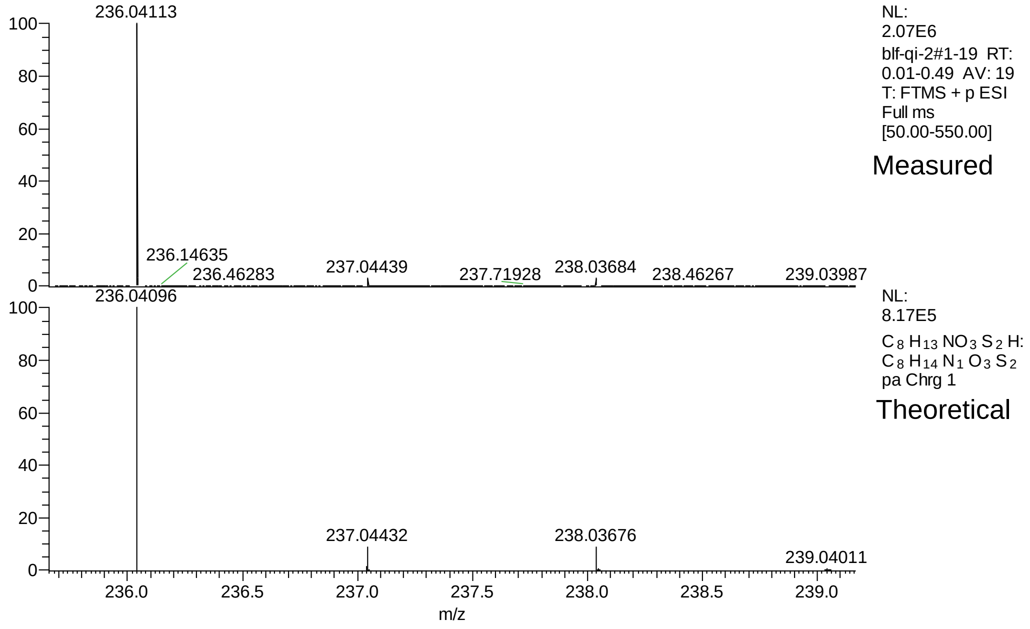

Fig. S98.

HR-MS of AA-L-Ala (Measured: 236.0411; Calculated: 236.0410)

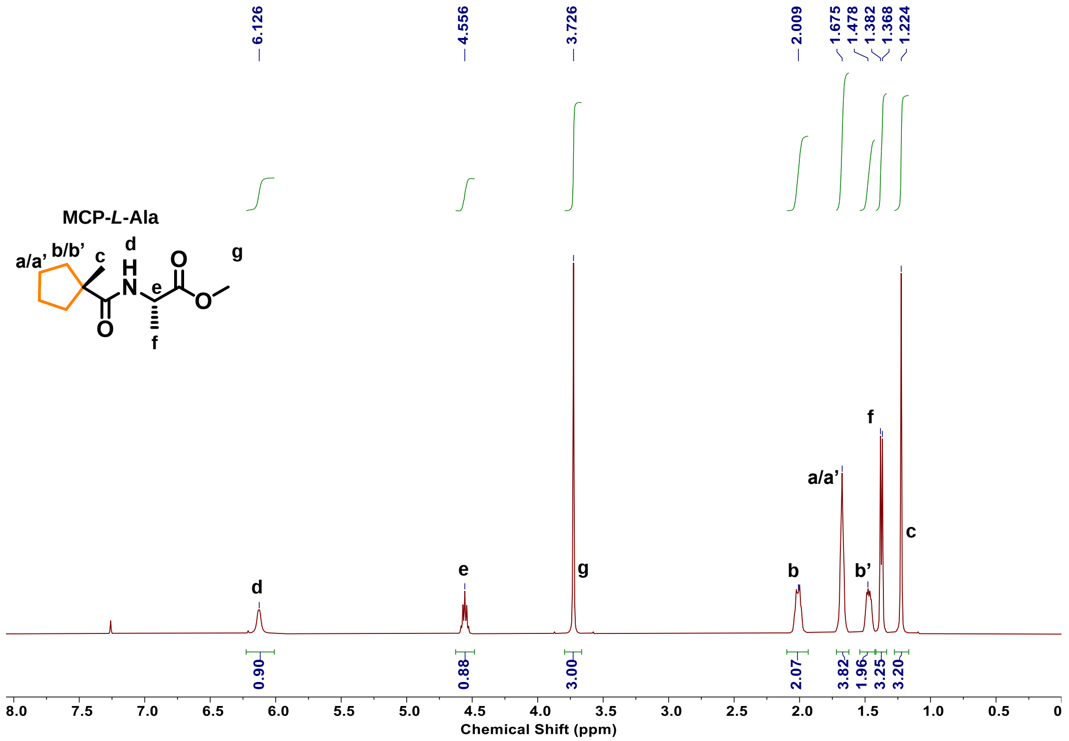

Fig. S99.

^{1}H NMR spectrum of MCP-*L*-Ala in $CDCl_3$ (500 MHz, 293K).

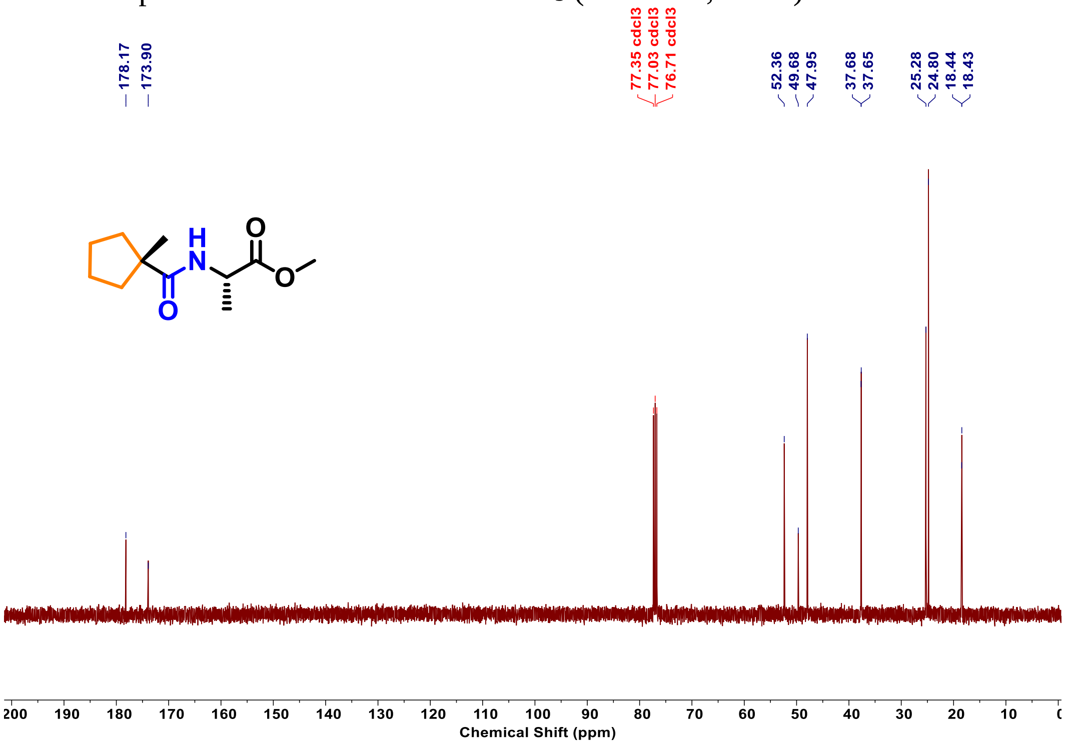

Fig. S100.

^{13}C NMR spectrum of MCP-*L*-Ala in $CDCl_3$ (101 MHz, 298K).

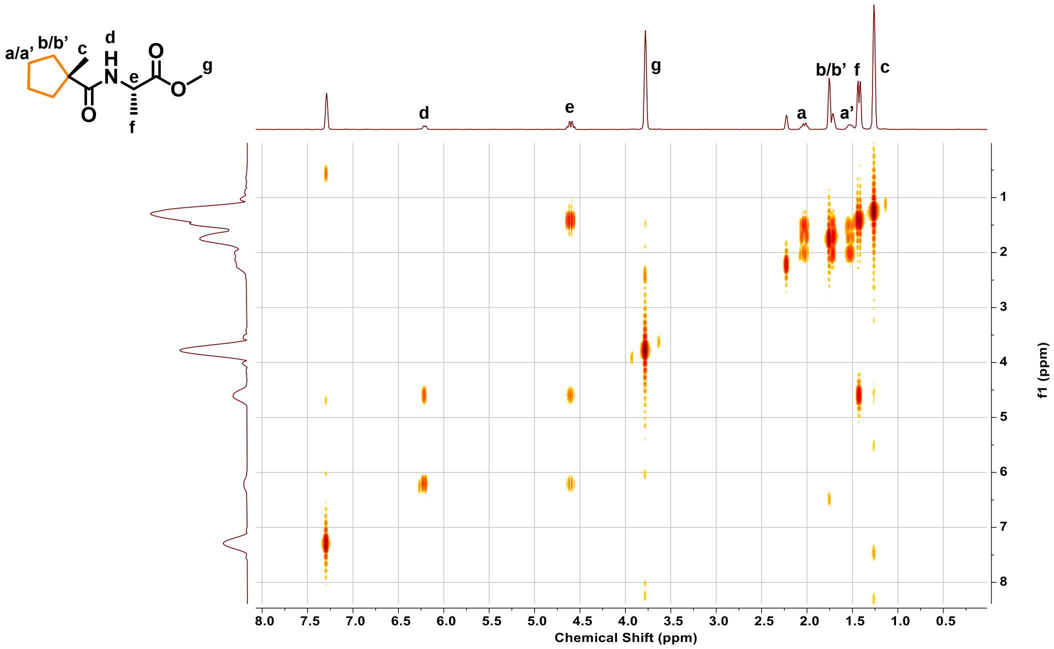

Fig. S101.

2D COSY spectrum of MCP-*L*-Ala in $CDCl_3$ (500 MHz, 253K).

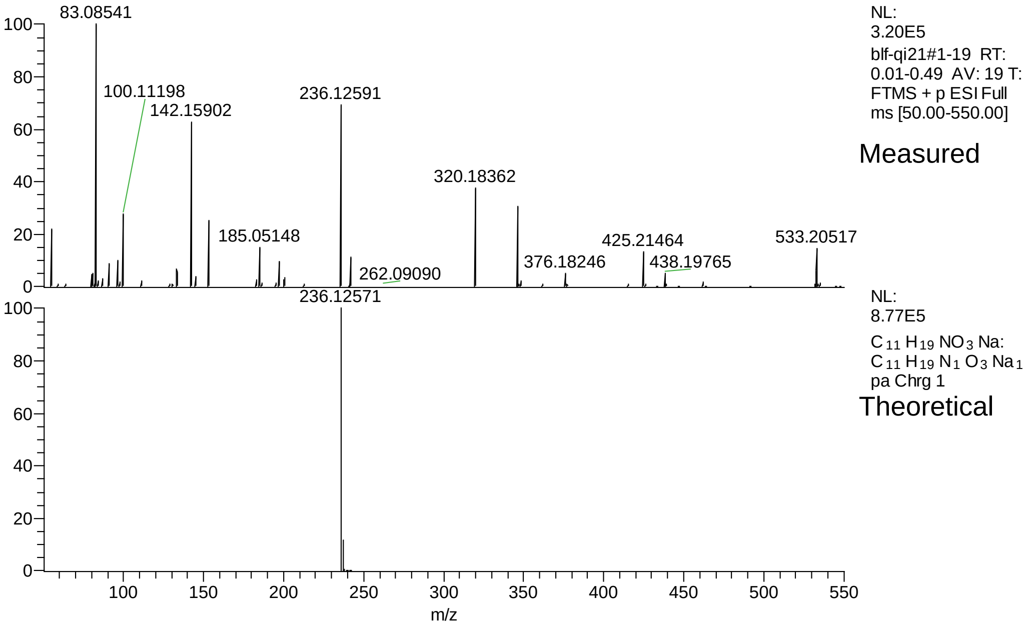

Fig. S102.

HR-MS of MAA-*L*-Ala (Measured: 236.1259; Calculated: 236.1257)

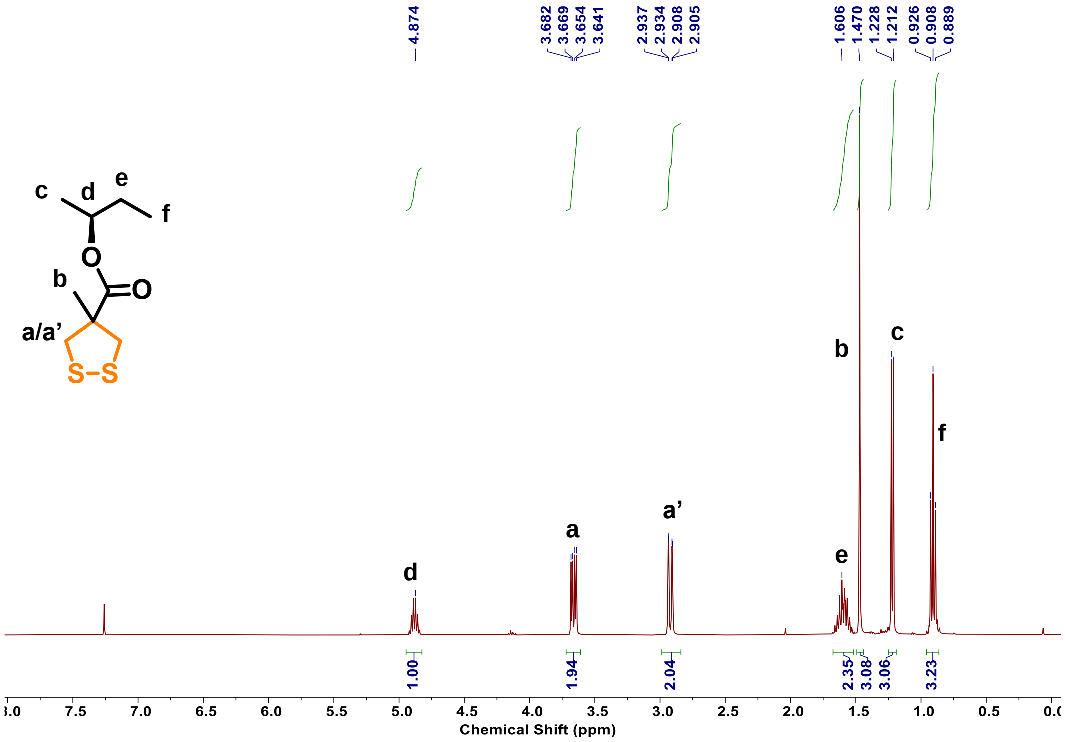

Fig. S103.

^{1}H NMR spectrum of MAA-*R*-Butyl-ester in $CDCl_3$ (400 MHz, 298K).

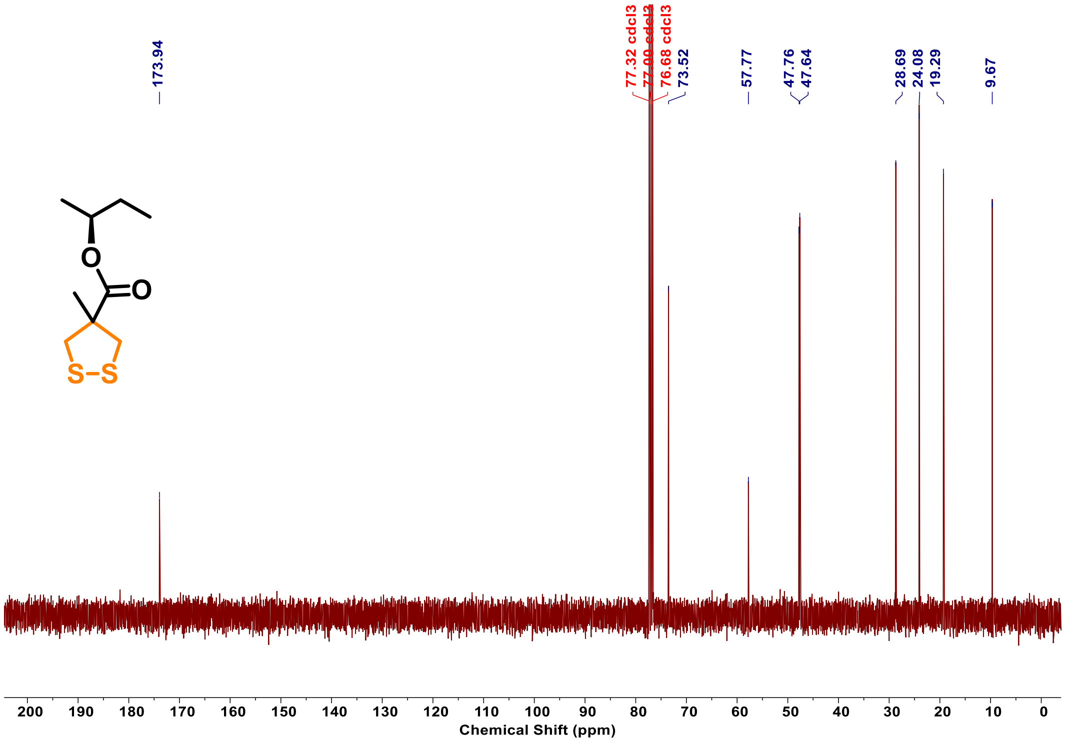

Fig. S104.

^{13}C NMR spectrum of MAA-*R*-Butyl-ester in $CDCl_3$ (101 MHz, 298K).

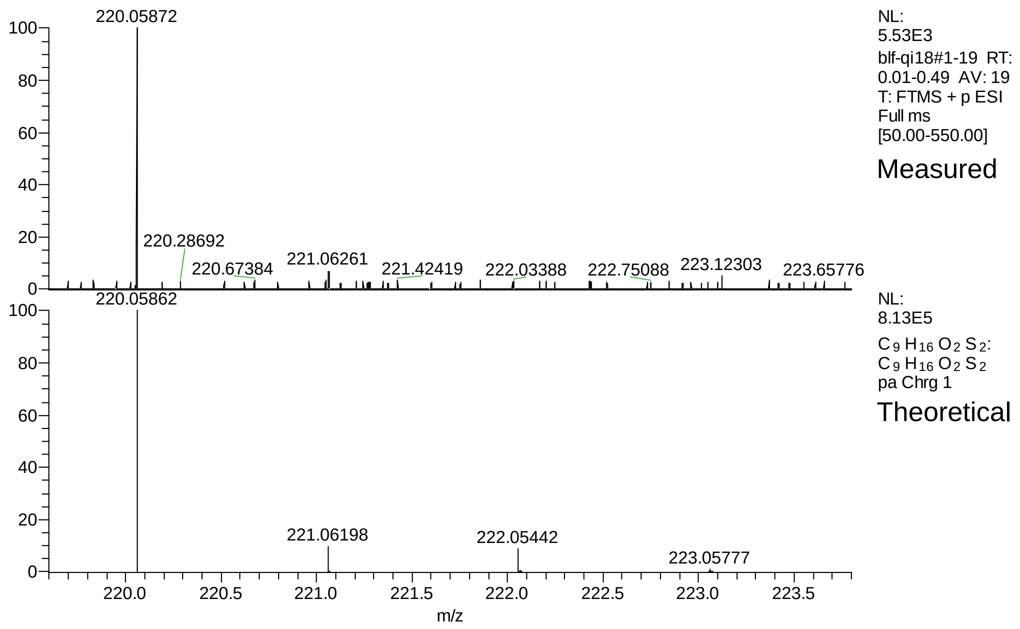

Fig. S105.

HR-MS of MAA-R-Butyl-ester (Measured: 220.0587; Calculated: 220.0586).

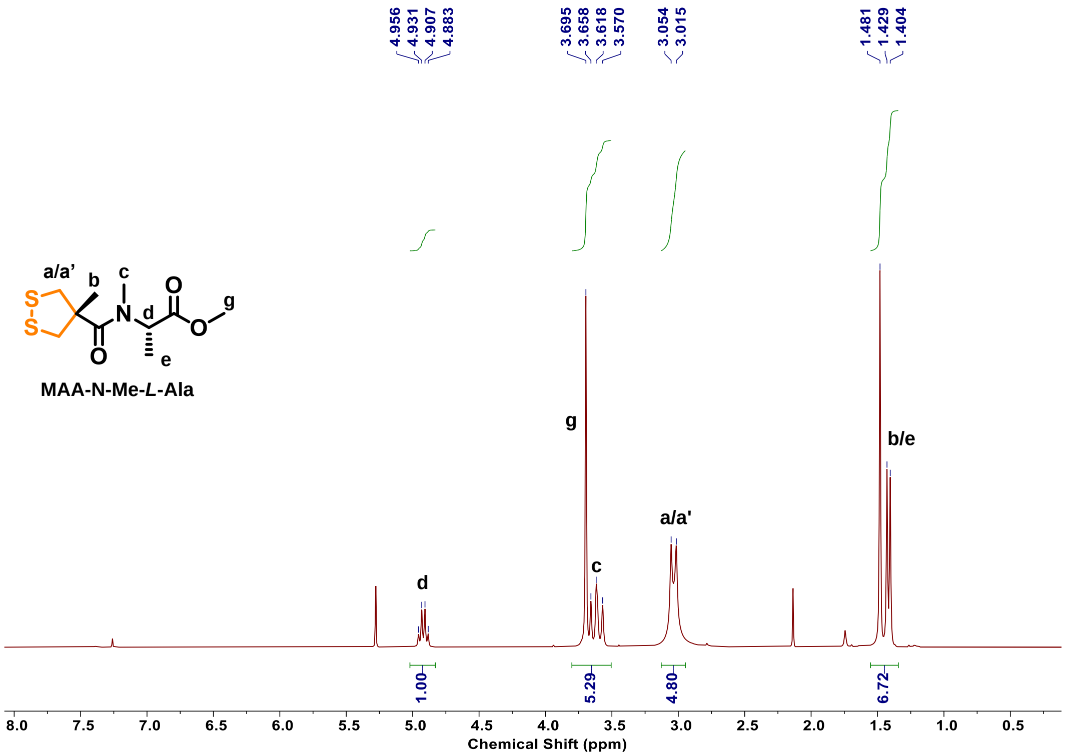

Fig. S106.

^{1}H NMR spectrum of MAA-N-Me-*L*-Ala in $CDCl_3$ (300 MHz, 298K).

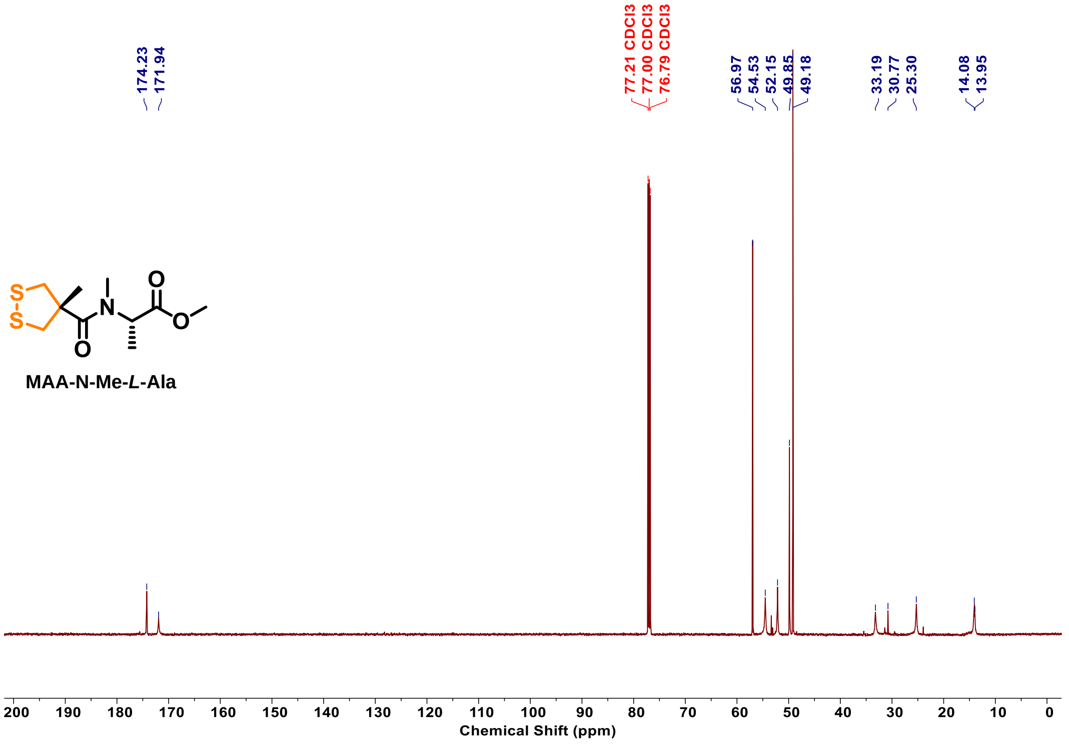

Fig. S107.

^{13}C NMR spectrum of MAA-N-Me-*L*-Ala in $CDCl_3$ (151 MHz, 298K).

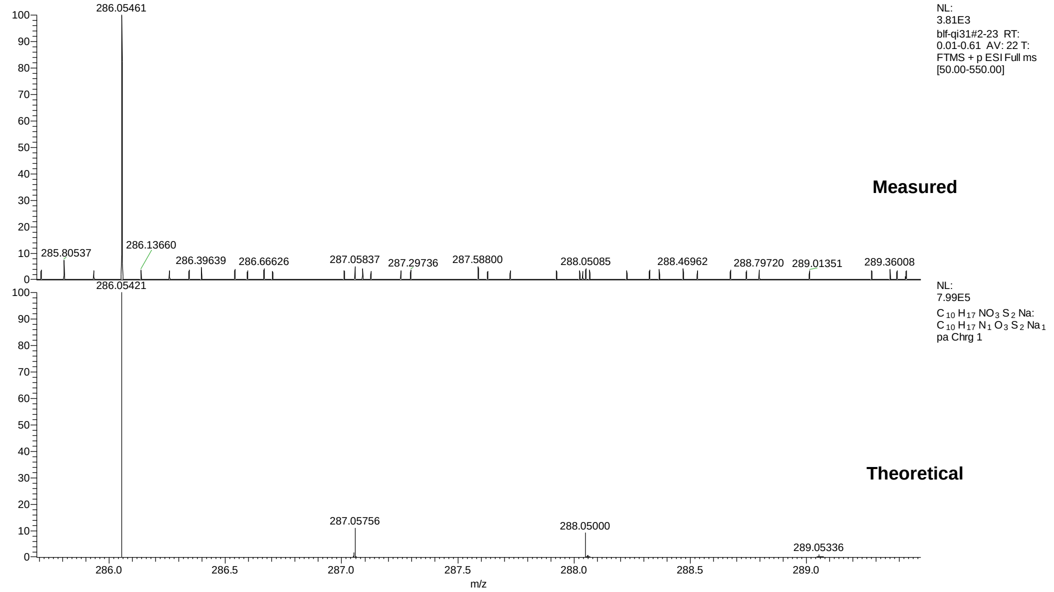

Fig. S108.

HR-MS of MAA-N-Me-*L*-Ala (Measured: 286.0546; Calculated: 286.0542).

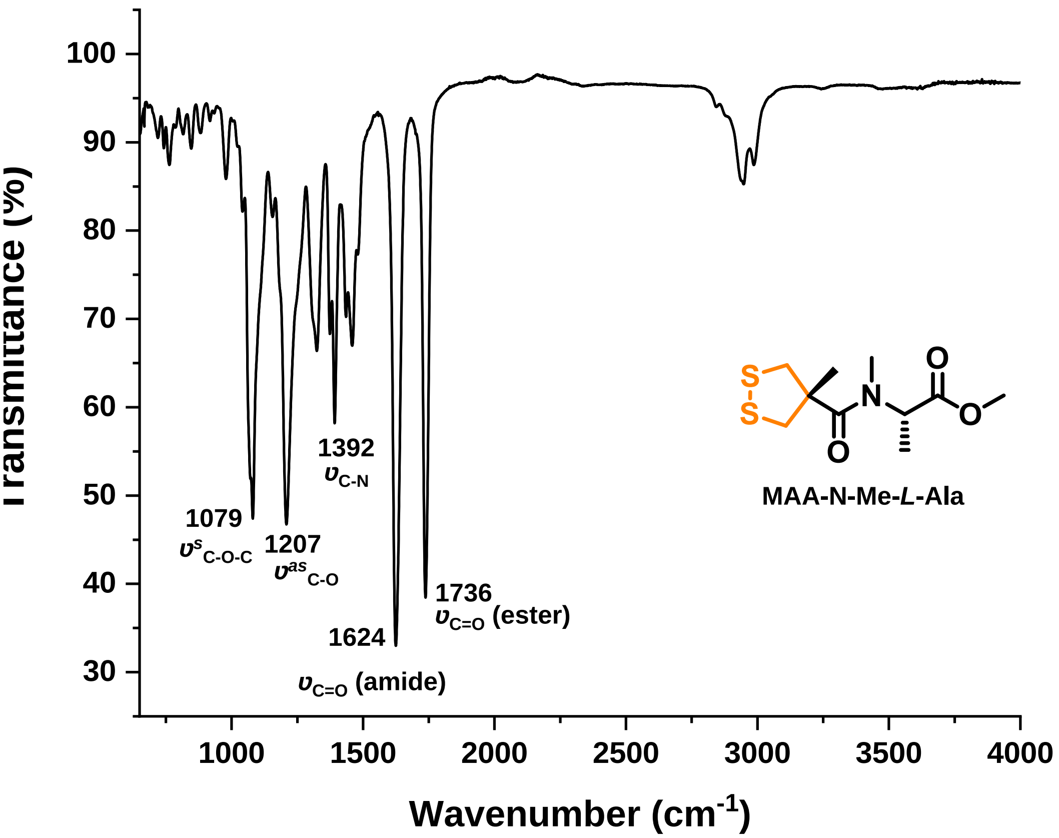

Fig. S109.

FT-IR (ATR) spectrum of MAA-N-Me-*L*-Ala. The distinctive vibration bands of tertiary amides and esters indicated the successful synthesis and high purity of the compound.

Supplementary References:

[1] Pracht, P., Bohle, F., Grimme, S. (2020). Automated exploration of the low-energy chemical space with fast quantum chemical methods. Phys. Chem. Chem. Phys. *22*, 7169-7192.

[2] Gaussian 16, Revision B.01, M. J. Frisch, G. W. Trucks, H. B. Schlegel, G. E. Scuseria, M. A. Robb, J. R. Cheeseman, G. Scalmani, V. Barone, G. A. Petersson, H. Nakatsuji, X. Li, M. Caricato, A. V. Marenich, J. Bloino, B. G. Janesko, R. Gomperts, B. Mennucci, H. P. Hratchian, J. V. Ortiz, A. F. Izmaylov, J. L. Sonnenberg, D. Williams-Young, F. Ding, F. Lipparini, F. Egidi, J. Goings, B. Peng, A. Petrone, T. Henderson, D. Ranasinghe, V. G. Zakrzewski, J. Gao, N. Rega, G. Zheng, W. Liang, M. Hada, M. Ehara, K. Toyota, R. Fukuda, J. Hasegawa, M. Ishida, T. Nakajima, Y. Honda, O. Kitao, H. Nakai, T. Vreven, K. Throssell, J. A. Montgomery, Jr., J. E. Peralta, F. Ogliaro, M. J. Bearpark, J. J. Heyd, E. N. Brothers, K. N. Kudin, V. N. Staroverov, T. A. Keith, R. Kobayashi, J. Normand, K. Raghavachari, A. P. Rendell, J. C. Burant, S. S. Iyengar, J. Tomasi, M. Cossi, J. M. Millam, M. Klene, C. Adamo, R. Cammi, J. W. Ochterski, R. L. Martin, K. Morokuma, O. Farkas, J. B. Foresman, and D. J. Fox, Gaussian, Inc., Wallingford CT, 2016.

[3] Bruker, (2016). *APEX3* (v2016.1-0), *SAINT* (Version 8.18C) and *SADABS* (Version 2012/1). Bruker AXS Inc., Madison, Wisconsin, USA.
[4] Sheldrick, G. M. (2015) *Acta Cryst.* **A71**, 3-8.
[5] Sheldrick, G. M. (2008). *Acta Cryst.* A**64**, 112-122.
